# Purified zymogens reveal mechanisms of snake venom metalloproteinase auto-activation

**DOI:** 10.7554/eLife.109112

**Published:** 2026-06-10

**Authors:** Sophie Hall, Iara Aime Cardoso, Mark C Wilkinson, Maria Molina Carretero, Srikanth Lingappa, Bronwyn Rand, Dakang Shen, Johara Boldrini-França, Richard Stenner, Stefanie Kate Menzies, Georgia Balchin, Konrad Kamil Hus, Renaud Vincentelli, Andrew Mumford, Alastair Poole, Nicholas R Casewell, Imre Berger, Christiane Schaffitzel

**Affiliations:** 1 School of Biochemistry, University of Bristol Bristol United Kingdom; 2 Centre for Snakebite Research and Interventions, Liverpool School of Tropical Medicine Liverpool United Kingdom; 3 School of Cellular and Molecular Medicine, University of Bristol Bristol United Kingdom; 4 Lancaster University Lancaster United Kingdom; 5 Department of Biotechnology and Bioinformatics, Faculty of Chemistry, Rzeszow University of Technology Rzeszow Poland; 6 Architecture et Fonction des Macromolécules Biologiques (AFMB), UMR 7257 CNRS-Aix-Marseille Université Marseille France; 7 Bristol Medical School, University of Bristol Bristol United Kingdom; 8 Max Planck Bristol Centre for Minimal Biology, School of Chemistry, University of Bristol Bristol United Kingdom; Department of Life Sciences, School of Natural Sciences, Shiv Nadar University India; University of Cambridge United Kingdom

**Keywords:** snake venom metalloproteinases, echis species, baculovirus insect cell expression, metalloprotease zymogens, SVMP auto--activation mechanisms, venom toxin biochemistry, Other

## Abstract

Snake venoms contain diverse mixtures of toxins that evolved to incapacitate prey, but in humans, they cause extensive pathology following snakebite envenomation. In viper venom, some of the most potent toxins are the haemorrhagic and coagulopathic snake venom metalloproteinases (SVMPs). Because venoms contain an SVMP cocktail and due to their cytotoxicity, SVMP characterisations have been hampered by the lack of purified enzymes. By incorporating their prodomain, which blocks the active SVMP site, we overcame their cytotoxicity and enabled recombinant production of zymogens from all three structurally variable SVMP classes (PI, PII, and PIII) using our baculovirus/insect cell expression system. Zymogens were auto-activated by incubation with Zn^2+^ ions, resulting in prodomain cleavage, PII disintegrin cleavage and PIII prodomain proteolysis. Auto-activated SVMPs were characterised using protein substrate degradation, platelet aggregation and blood coagulation assays, benchmarked to native venom-purified SVMP. Our recombinant zymogen production protocol is generically applicable for the expression of SVMPs, unlocking biomedical use in haematology and discovery of novel snakebite therapeutics.

## Introduction

**Figure 1.**
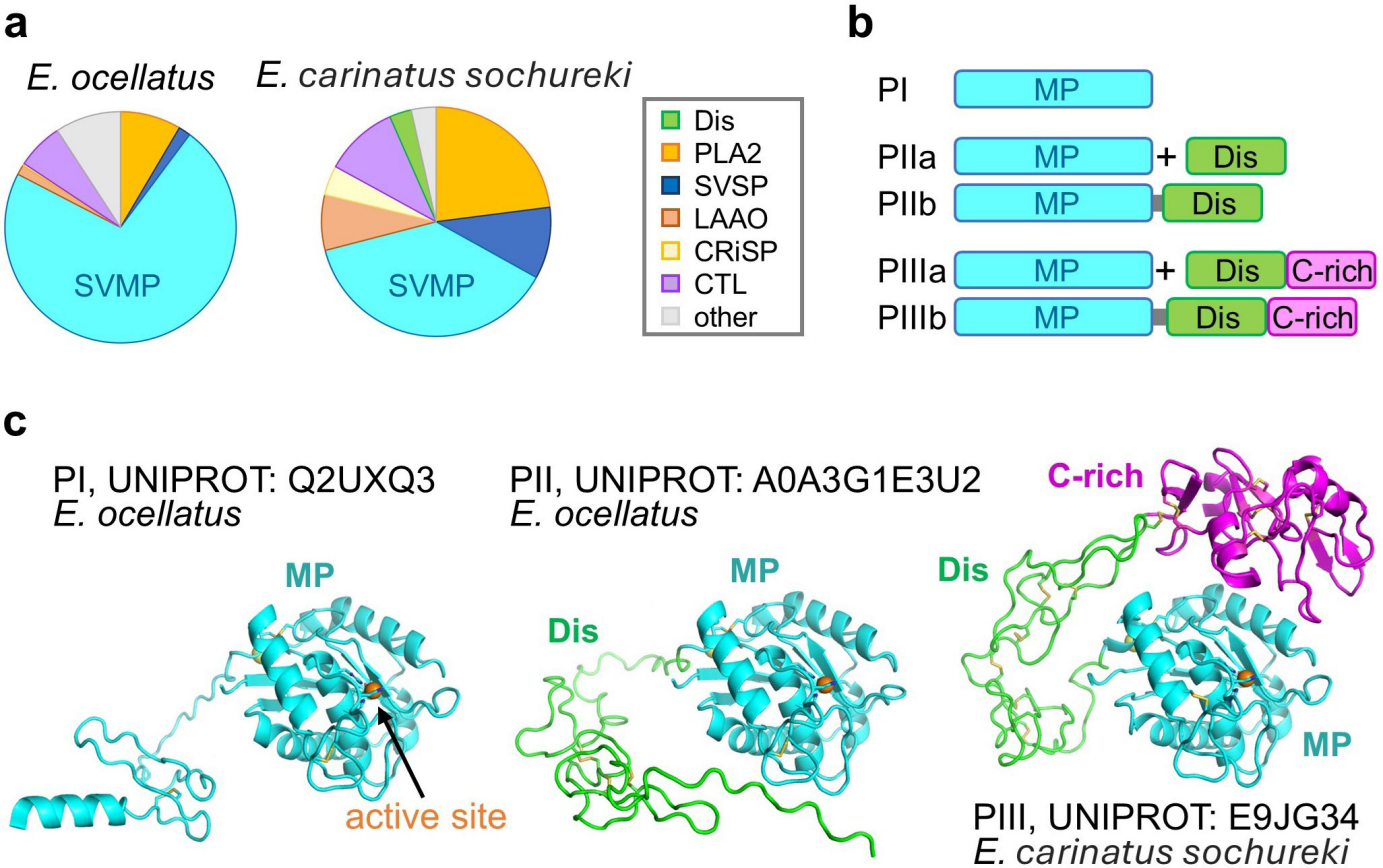
Snake venom metalloproteinase (SVMP) classification and abundance in *Echis* venom. (**a**) Pie charts displaying *Echis ocellatus* and *Echis carinatus sochureki* venom composition, with SVMP coloured in cyan (Oliveira et al., 2022). (**b**) Simplified schematic of mature PI, PII, and PIII SVMP architecture, showing metalloproteinase domain (MP, cyan), disintegrin domain (Dis, green), and cysteine-rich domain (C-rich, magenta). (**c**) AlphaFold 3 predicted structural models of PI and PII SVMPs from *E. ocellatus*, and PIII SVMP from *E. carinatus sochureki*. Domain names and colour coding as in panel b. Zn^2+^ ions (orange) in the active site are shown as spheres.

Snake venom is a complex cocktail of more than 100 proteins and peptides belonging to various toxin families (Oliveira et al., 2022). Although the number and abundance of isoforms within these families vary extensively across different venomous snakes, the snake venom metalloproteinases (SVMPs) are a pathologically important toxin family present in almost all snake venoms. SVMPs play a significant role in the physiological effects resulting from both local and systemic envenoming, such as haemorrhage, coagulopathy, and tissue necrosis (Bittenbinder et al., 2024). In many medically important viperid snake genera such as *Echis* (saw-scaled vipers), SVMP toxins are the most abundant toxin constituents, on average accounting for 33% of all toxins, although this can be as high as 72% in *Echis ocellatus* (Wagstaff et al., 2009; Casewell et al., 2014; Tasoulis and Isbister, 2023; Figure 1a).

**Figure 1—figure supplement 1.**
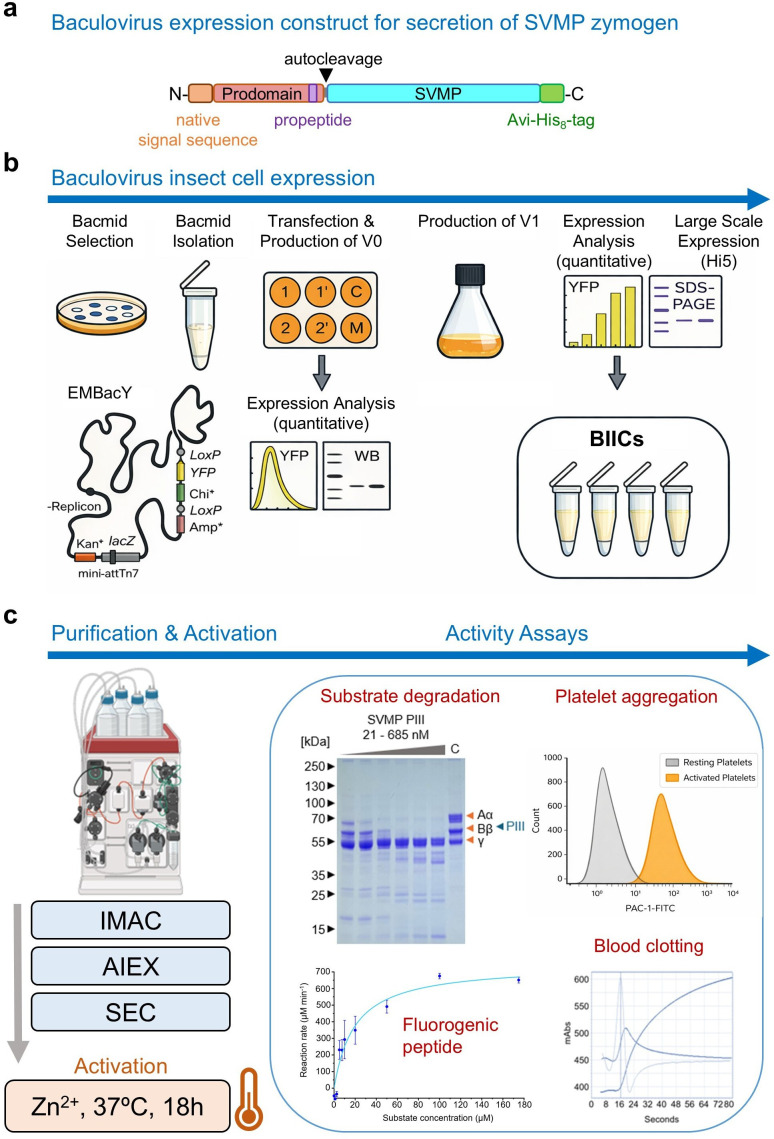
Schematic outlining snake venom metalloproteinase (SVMP) production and characterisation strategy. (**a**) Design of the expression construct for secretion of SVMP zymogens, comprising the native signal sequence, the endogenous prodomain with propeptide, the SVMP, and a C-terminal Avi- and octa-histidine tags for biotinylation and affinity purification, respectively. (**b**) Baculovirus insect cell expression timeline (adapted from Figure 3 in Trowitzsch et al., 2010). Plasmids encoding the SVMP zymogen constructs are integrated into EMBacY baculoviral DNA via Tn7 transposition. Positive clones are identified by blue/white screening. EMBacY baculoviral DNA (single white colonies) is isolated and used to transfect Sf21 cells. Media containing initial virus (V_0_) is removed from the wells and used for infecting insect cell cultures (25 ml volume) in an Erlenmeyer shaker flask. Protein production is followed by the yellow fluorescent protein (YFP) fluorescence signal and by western blot (WB) using anti-His antibodies. Infected Hi5 cell cultures in shaker flasks are split every 24 hr until cell proliferation arrest (PA) occurs. Media containing amplified virus (V_1_) is removed ∼48 hr after PA, and fresh medium is replenished instead. Cells are harvested when the YFP signal has reached a plateau (typically after 3–4 days). Protein production is analysed by SDS-PAGE. Baculovirus-infected insect cell (BIIC) stocks are prepared for long-term storage of viruses. The whole procedure takes less than 2 weeks. (**c**) SVMP zymogens are secreted and purified from the medium using three consecutive chromatography steps. Activation is accomplished by incubation with Zn^2+^, the concentration of Zn^2+^ required for full activation of the zymogen has to be optimised for each individual SVMP. Activated SVMPs are then used for activity assays, including protein degradation, cleavage of fluorogenic peptide, blood clotting, and platelet aggregation assays (for more details see Materials and methods). The amount of SVMP required for each assay has to be optimised.

SVMPs are complex, structurally variable, cysteine-rich, Zn^2+^-dependent metalloproteinases (MPs) that can be classified into three sub-classes based on domain architecture: PI (comprising MP domain only), PII (MP and disintegrin domains), and PIII (MP, disintegrin-like, and cysteine-rich domains; Figure 1b); representatives of all three sub-classes are present in different viper venoms (Fox and Serrano, 2008; Casewell et al., 2011).

SVMPs are particularly toxic proteins with a wide range of substrates. They are known to degrade the basement membrane and extracellular matrix proteins, as well as several blood clotting factors, most notably fibrinogen, through their MP domain (Asega et al., 2020; Kini and Koh, 2016). Extracellular matrix breakdown and loss in blood capillary wall integrity results in vascular leakage, resulting in both local and systemic haemorrhage (Gutiérrez et al., 2005) and in the spread of other venom toxins (Gutiérrez et al., 2016). In PII SVMPs, the disintegrin domain contains a charged, integrin-binding motif, most commonly an arginine-glycine-aspartate (RGD) motif, which binds, among others, the fibrinogen receptor integrin α_IIb_β_3_ (de O Almeida et al., 2023), causing inhibition of platelet aggregation.

Because viper SVMPs often target components of the coagulation cascade with unique specificity and/or lack of reliance on typically essential co-factors, these enzymes are highly valuable for investigating and monitoring the treatment of pathologies related to haemostasis (e.g. factor deficiencies, bleeding disorders; Moore, 2022). In the clinic, the SVMPs RVV-X and Ecarin are both used as standards for coagulation tests (Slagboom et al., 2017). In addition, SVMP-derived disintegrins have been repurposed for use as, or inspiration for the design of, anti-platelet therapies to treat atherothrombosis in the form of unstable angina and myocardial infarction (Oliveira et al., 2022). Thus, a single snake venom toxin family has yielded several translationally valuable tools for diagnosis and treatment of human disease. However, further exploitation of SVMPs is hampered by the considerable challenge in purifying individual SVMP proteins from venom, and the lack of robust, scalable, recombinant toxin production protocols, representing a bottleneck.

Currently, SVMPs are typically purified from venom by size exclusion chromatography (SEC), followed by ion exchange chromatography of the peaks containing SVMPs. Further separation can be achieved by hydrophobic interaction chromatography or reversed-phase high-performance liquid chromatography (RP-HPLC; Wilkinson et al., 2024; Yee et al., 2016). However, complete separation of endogenous functionally active SVMPs is difficult to achieve, because: (i) many venoms contain multiple related SVMP isoforms that share similar physicochemical properties, (ii) the resulting yields of each particular SVMP isoform can be very low, and (iii) the use of some desirable separation approaches (e.g. RP-HPLC) can result in SVMP denaturation. Importantly, this procedure is impeded by the limited access to sufficient amounts of extracted snake venom.

Recombinant expression of SVMPs could resolve the bottleneck but is severely hampered by their cytotoxicity, domain complexity, and high number of cysteine residues that complicate protein folding – for example, the PIII SVMPs contain between 20 and 40 cysteines forming disulfides (Olaoba et al., 2020). Currently, protocols are available for the production of only a select few specific SVMPs (including albocollagenase, bothropasin, and Ecarin; Pinyachat et al., 2011; Assakura et al., 2003; Jonebring et al., 2012). However, these suffer from limitations such as low yield and inactive proteins. Zymogen production of SVMPs has been described before, but it remains generally elusive how the zymogen is processed into the mature, active SVMP. Current protocols describe incubation of the zymogen for >7 days (Jonebring et al., 2012), incubation with other active proteinase toxins (Shimokawa et al., 1996), or the insertion of a TEV protease cleavage site into the SVMP amino acid sequence (Camacho et al., 2019).

Here, we establish a novel and generic protocol to access functional PI, PII, and PIII SVMPs in the quality and quantity required for characterisations and show that all three classes of SVMPs can auto-activate in the presence of zinc ions, resulting in distinct outcomes.

We used our MultiBac baculovirus/insect cell expression system (Bieniossek et al., 2012) for recombinant SVMP zymogen production. The MultiBac system is well-established for secretion of proteins, including the C-terminal heavy chain domain of the highly toxic protein, clostridial botulinum neurotoxin (Villaflores et al., 2013). We postulate that this system is highly suitable for SVMP production because (i) folding of the multi-domain SVMP zymogen will be supported by eukaryotic, endogenous chaperones present in insect cells and (ii) secreted SVMPs will undergo cellular protein quality control in the endoplasmic reticulum (ER) and Golgi apparatus for correct disulfide bond formation and glycosylation before secretion, ensuring that the toxins produced are correctly folded, soluble and authentically modified. We show that zymogen expression overcomes SVMP cytotoxicity, and we demonstrate successful production of PI and PII SVMPs from *E. ocellatus* and a PIII SVMP from *E. carinatus sochureki* (Figure 1b and c). We establish auto-activation of the SVMP zymogens and concomitant cleavage of the prodomain (all classes), C-terminal tags (PI, PII), and the disintegrin domain (PII). The PIII prodomain is completely degraded by the activated SVMP. We further analyse the cytotoxicity and haemotoxicity of the auto-activated SVMPs *in vitro* using established casein (Macêdo and Fox, 2016), fibrinogen (Macêdo and Fox, 2016), fluorogenic peptide (Alsolaiss et al., 2022), and insulin degradation (Wilkinson et al., 2024) assays. For the PI SVMP, we show that the substrate specificity of recombinant SVMP is identical to that of the corresponding toxin purified from venom, compellingly validating the utility of our strategy.

Our generic SVMP production protocol thus paves the way to systematically characterise SVMPs and unlock biomedical applications, in haematology, thrombosis and inflammation research and as targets for the discovery of novel snakebite therapeutics.

## Results

### Recombinant SVMP zymogen expression overcomes their cytotoxicity

For our expression trials, we chose PI, PII, and PIII SVMPs which, according to transcriptomics (Redureau et al., 2025), are highly expressed in the venom gland of medically important saw-scaled vipers. We generated baculovirus/insect cell expression constructs for Q2UXQ3 (PI), A0A3G1E3U2 (PII), and E9JG34 (PIII) (Figure 1c), each encoding a signal sequence for secretion, the toxin of interest and C-terminal tandem octa-histidine- and Avi-tags, codon optimised for both *Spodoptera frugiperda* and *Trichoplusia Ni* as expression hosts (Figure 1—figure supplement 1). Initially, recombinant production of mature SVMPs, without the N-terminally fused prodomain, was attempted (Figure 2—figure supplement 1a). Protein expression yields, however, were extremely low, primarily due to SVMP cytotoxicity causing pervasive cell death immediately upon baculoviral infection due to initial trace amounts of active SVMP. Subsequent expression strategies to produce SVMPs were aimed at inactivating the MP by adding an inhibitor, native propeptides or SVMP active site mutants (Appendix). Marimastat, a broad-spectrum MP inhibitor (Tessier et al., 1991) could not prevent cell death induced by PIII SVMP expression. On the contrary, the cell viability in the presence of 3 µM and 6 µM Marimastat was even further decreased (Figure 2—figure supplement 1b). Similarly, active site mutants in the PIII SVMP still showed significant toxicity, markedly reducing yellow fluorescent protein (YFP) levels, which serve as a proxy for heterologous protein production, as well as cell viability (Figure 2—figure supplement 1c).

**Figure 2.**
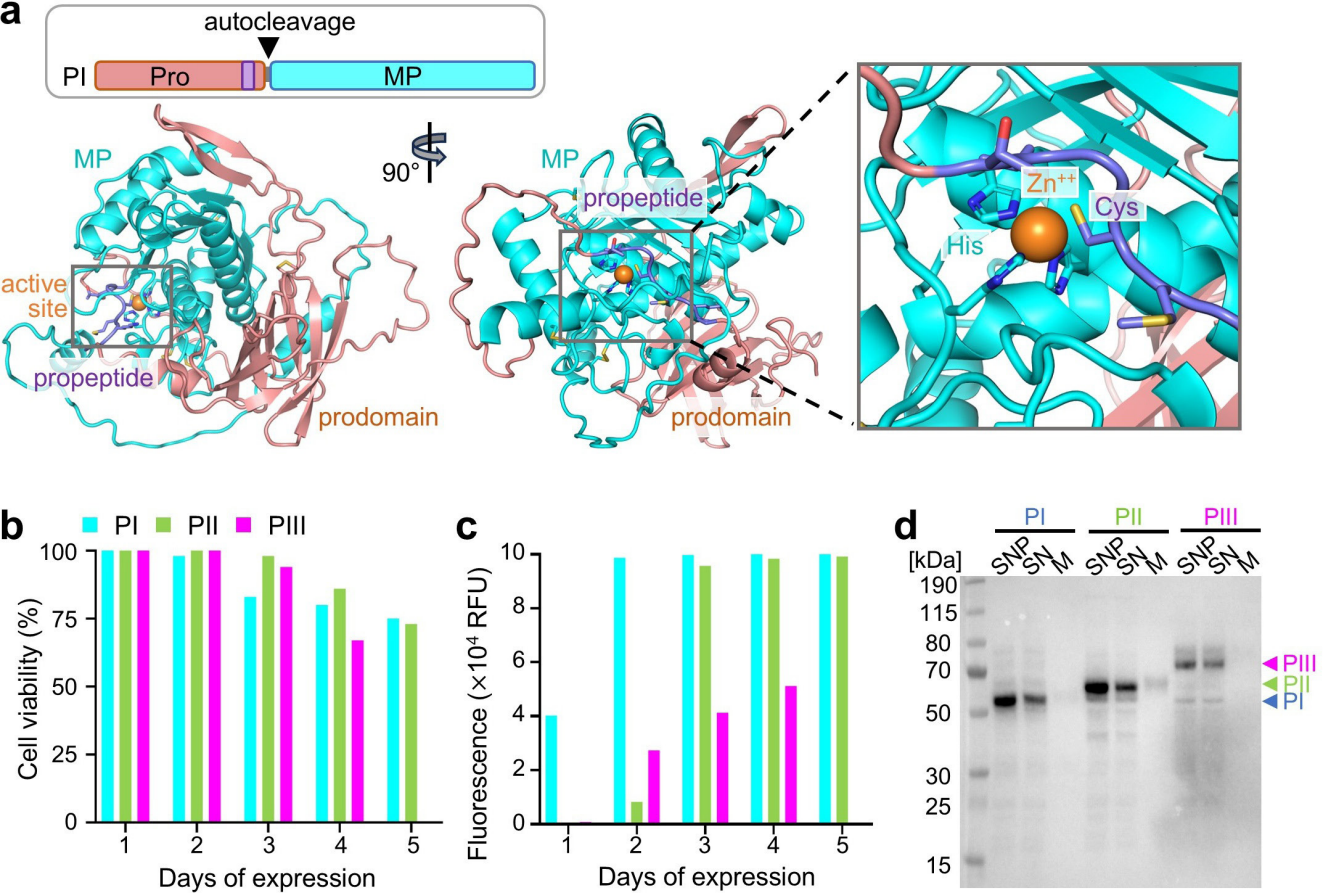
PI, PII, and PIII snake venom metalloproteinase (SVMP) zymogen expression. (**a**) Above: Schematic of PI SVMP zymogen with N-terminal prodomain (Pro, salmon), propeptide (purple). Below: left: two views of AlphaFold 3 prediction of PI zymogen model; right: zoom in on active site Zn^2+^ ion coordinated by histidines and propeptide cysteine. Expression of SVMP zymogens monitored by (**b**) cell viability, (**c**) yellow fluorescent protein (YFP) fluorescence (PIII harvested on day 4) and (**d**) western blot analysis using HRP-conjugated anti-Penta-His antibody. SNP: supernatant + pellet, SN: supernatant, M: media (10 x concentrated). Cyan: PI SVMP, green: PII SVMP, magenta: PIII SVMP. SVMP zymogen expression was repeated five times. Figure 2—source data 1.Original file for western blot analysis displayed in Figure 2d. Figure 2—source data 2.PDF file containing original western blot for Figure 2d, indicating the relevant bands. Figure 2—source data 3.Excel file containing YPF fluorescence reads and cell viability counts for Figure 2b and c.

In the snake venom gland, SVMPs are produced with an N-terminally fused prodomain that is removed through cleavage during post-translational processing of the protein (Figure 2a). The prodomain comprises a small, highly conserved propeptide sequence (usually PKMCGVT) – this propeptide acts as a cysteine switch to maintain enzyme latency (Grams et al., 1993). AlphaFold 3 (Abramson et al., 2024) predicts that the prodomain adopts a β-barrel structure and folds around the MP domain, slotting the propeptide into the active site of the MP domain, where the propeptide cysteine co-ordinates the active site Zn^2+^ ion, along with the three active site histidines (Figure 2a). This renders the MP inactive as the Zn^2+^ ion cannot be accessed by substrates in the active site.

**Figure 2—figure supplement 1.**
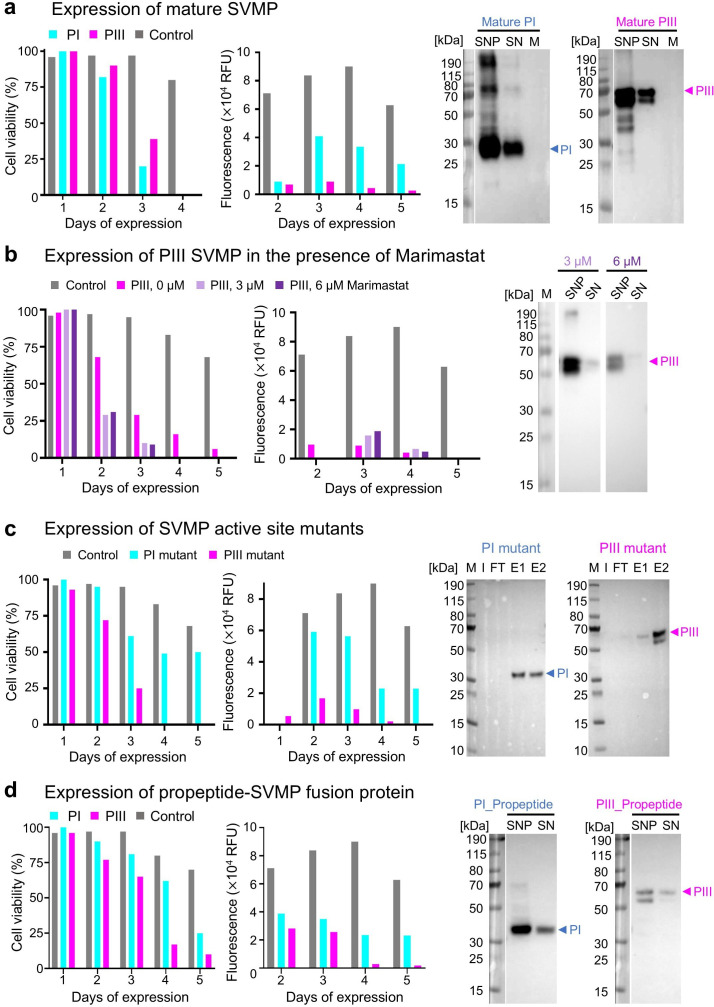
Optimisation of snake venom metalloproteinase (SVMP) expression using mature SVMP, broad-spectrum inhibitor, SVMP active site mutants, and propeptide-SVMP fusion protein to overcome SVMP cytotoxicity. (**a**) Expression of mature SVMPs monitored by cell viability (left), yellow fluorescent protein (YFP) fluorescence (middle), and western blot analysis of His-tagged protein (right). SNP: supernatant + pellet; SN: supernatant, M: medium. Grey bars: non-toxic protein control expression, cyan: PI SVMP, and magenta: PIII SVMP. (**b**) Expression of mature PIII SVMP in the presence of 0 μM (magenta), 3 μM (lilac), or 6 μM (purple) Marimastat. Expression is monitored by cell viability (left), YFP fluorescence (middle), and western blot analysis of His-tagged protein (right). (**c**) Expression of SVMP active site mutants monitored by cell viability (left) and YFP fluorescence (middle). Right: western blot analysis of expressed His-tagged protein following Ni-NTA purification. I: input; FT: flowthrough; E1, E2: elution fractions. (**d**) SVMP propeptide-fusion expression monitored by cell viability (left), YFP fluorescence (middle), and western blot analysis of His-tagged protein (right). Grey bars: non-toxic protein expression control, cyan: PI SVMP, and magenta: PIII SVMP. HRP-conjugated anti-Penta-His antibody was used for all western blots. Experiments were performed at least in duplicate. Figure 2—figure supplement 1—source data 1.Original files for western blot analysis displayed in Figure 2—figure supplement 1. Figure 2—figure supplement 1—source data 2.PDF file containing original western blots for Figure 2—figure supplement 1, indicating the relevant bands. Figure 2—figure supplement 1—source data 3.Excel file containing YPF fluorescence reads and cell viability counts for Figure 2—figure supplement 1a–d.

**Figure 2—figure supplement 2.**
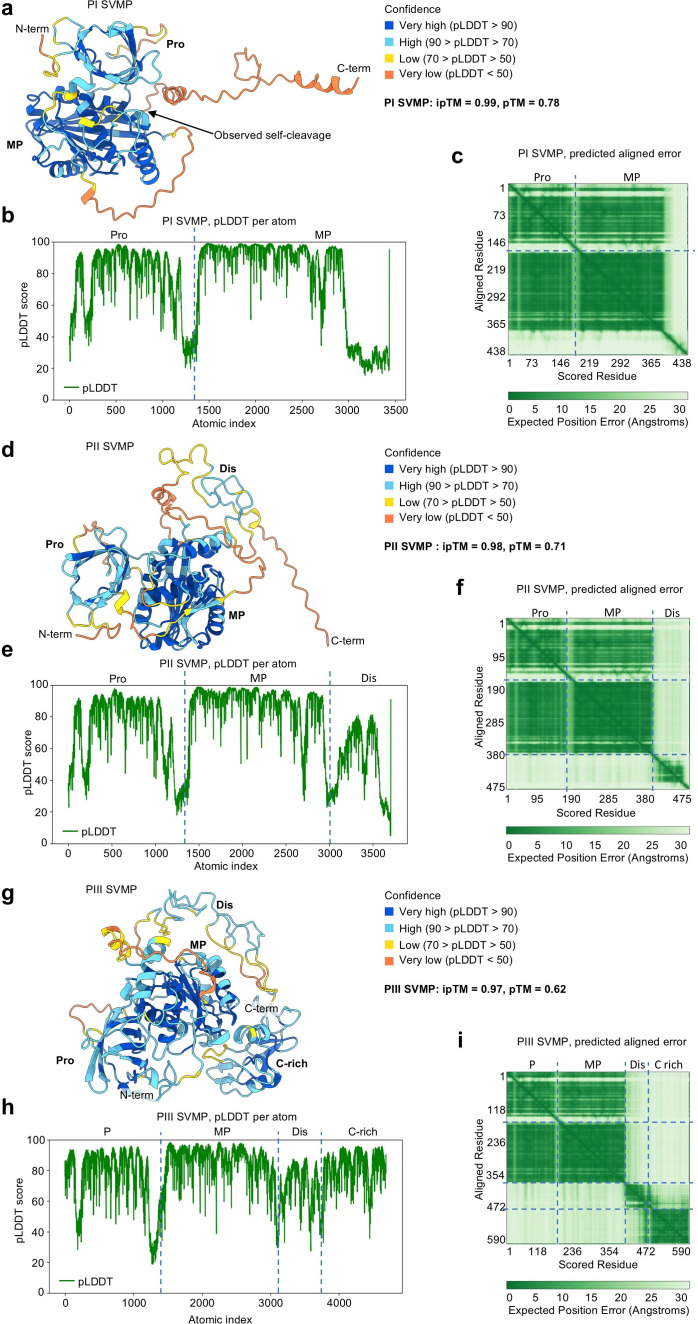
AlphaFold 3 structural predictions of SVMP zymogens. Structural predictions of SVMP zymogens of (**a–c**) PI, (**d-f**) PII, and (**g–i**) PIII class, performed by AlphaFold 3 (Abramson et al., 2024) in the presence of Zn^2+^, along with the corresponding predicted Local Distance Difference Test (pLDDT) (**b, e, h**) and predicted Aligned Error (pAE) (**c, f, i**) plots.

Inspired by this, we designed propeptide-SVMP fusion proteins with three propeptide repeats to the N-terminus of the SVMP followed by a TEV protease cleavage site. We hypothesised that this would increase the avidity of the propeptide to the MP active site and efficiently inactivate the enzyme. While cell viability and YFP fluorescence levels for PI and PIII slightly increased (Figure 2—figure supplement 1d), compared to expression of the mature SVMP (Figure 2—figure supplement 1a), YFP expression was still significantly lower as compared to a positive control which expressed an unrelated, non-toxic protein. Western blot analysis confirmed soluble expression of PI and PIII SVMPs (Figure 2—figure supplement 1d), but protein yields in the media remained too low to progress to purification.

Therefore, we produced complete SVMP zymogens, where the native signal sequence and the entire prodomain including propeptide is expressed N-terminally to the MP domain (Figure 2, Figure 1—figure supplement 1), adopting a compact structure as predicted by AlphaFold 3 (Abramson et al., 2024; Figure 2—figure supplement 2). Cells expressing PI, PII, and PIII SVMPs as zymogens showed clearly improved cell viability, remaining above 60% on day 4 of expression (Figure 2b). PIII SVMP zymogen was harvested on day 4 to avoid additional cell death and lysis. In contrast, the cells expressing PI and PII SVMPs remained ~75% viable on day 5 of expression when the SVMPs were harvested. Notably, the zymogen-expressing cells had considerably higher YFP readings as compared to the cells previously expressing mature PI and PIII SVMPs, and no loss of YFP fluorescence over time was observed (Figure 2c). Western blot analysis using an anti-His-tag antibody confirmed the presence of PI, PII, and PIII zymogens in cell extract and lysate, and zymogen protein was also detected in the concentrated media fraction, indicating successful secretion (Figure 2d). We conclude that SVMPs require the full-length N-terminal prodomain to ensure enzyme latency is maintained, and to allow for significant amounts of protein to be produced and secreted when recombinantly expressed using a baculovirus/insect cell system.

### SVMP zymogens can be produced in milligram amounts

**Figure 3.**
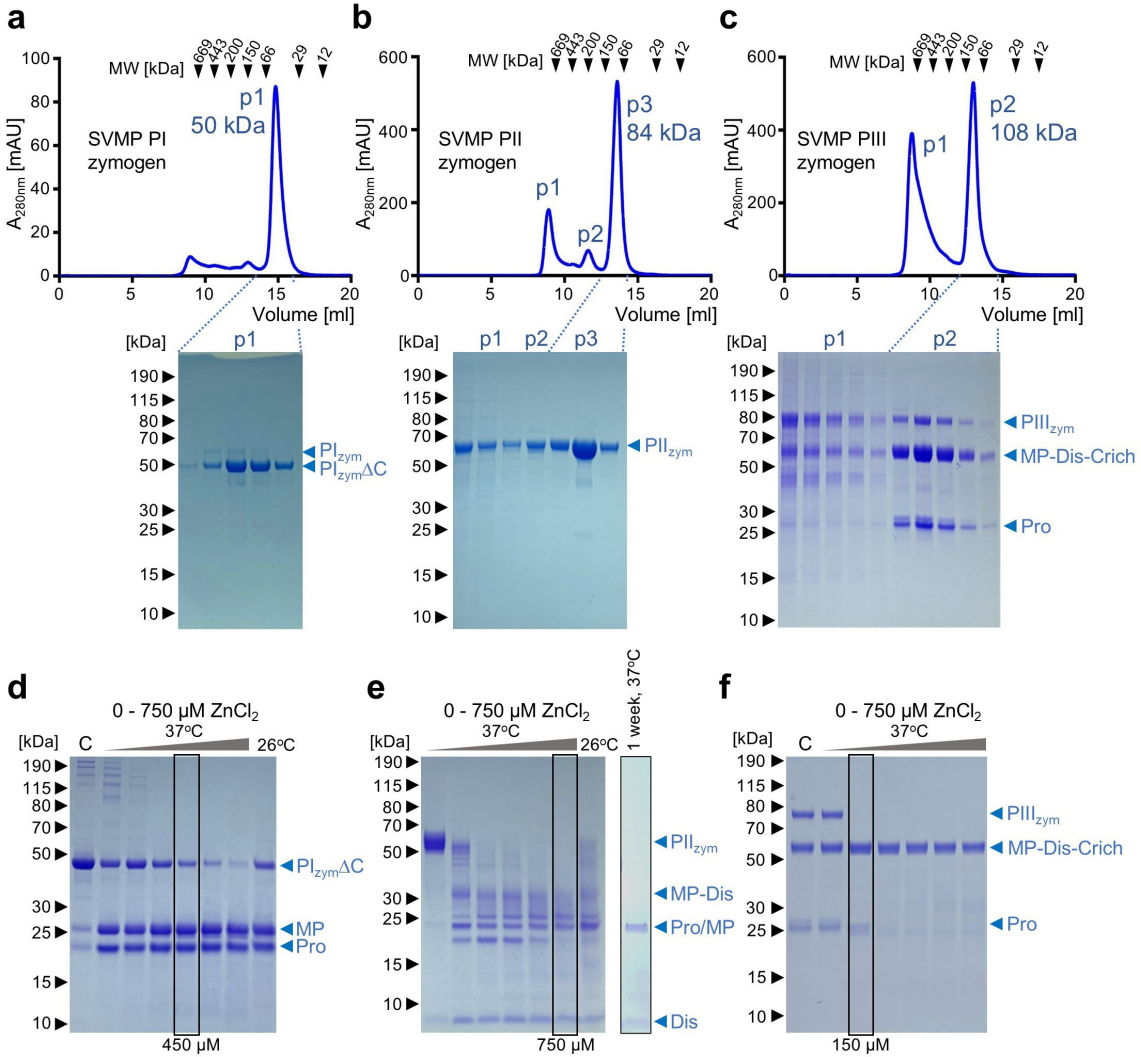
Size exclusion chromatography (SEC) purification of snake venom metalloproteinase (SVMP) zymogens and auto-activation. Size exclusion chromatograms and SDS-PAGE of (**a**) PI zymogen (PI_zym_ΔC lacks C-terminal tags), (**b**) PII zymogen, and (**c**) PIII zymogen after IMAC and IEX. PIII_zym_ partial cleavage separates the prodomain (Pro) and the mature PIII. Elution volumes of molecular weight (MW) calibration markers are indicated in the chromatograms as black arrows. (**d**) Activation of PI zymogen into metalloproteinase and prodomain. C (control): no 18 hr incubation. (**e**) Activation of PII zymogen into metalloproteinase-disintegrin and disintegrin domain (prodomain cannot be definitively identified). After 1 week, only 8 kDa and 23 kDa bands remain. (**f**) Activation of PIII zymogen into prodomain and mature PIII. At higher Zn^2+^ concentrations, the prodomain is degraded. C (control): no 18 hr incubation. SVMP zymogen purifications and activations were repeated five times. Figure 3—source data 1.Original files for original SDS-PAGE gels displayed in Figure 3a–f. Figure 3—source data 2.PDF file containing original SDS-PAGE gels for Figure 3a–f, indicating the relevant bands. Figure 3—source data 3.Excel sheet containing the chromotograms for the affinity (IMAC), IEX and SEC purification of PI SVMP zymogen shown in Figure 3a. Figure 3—source data 4.Excel sheet containing the chromotograms for the affinity (IMAC), IEX and SEC purification of PIII SVMP zymogen shown in Figure 3c. Figure 3—source data 5.Excel sheet containing the chromotograms for the affinity (IMAC), IEX and SEC purification of PIII SVMP zymogen shown in Figure 3c.

Following secretion of the SVMP zymogens into the media, a three-step purification was performed (Figure 1—figure supplement 1). Firstly, cells were pelleted by centrifugation, and the media containing secreted proteins were passed through a HiTrap IMAC column for metal affinity purification to enrich the secreted SVMPs from the comparatively large culture media volume. SVMPs were eluted using imidazole. Fractions containing SVMP zymogen were dialyzed and further purified by anion exchange chromatography (IEX), followed by SEC. SVMP zymogens generally eluted in one prominent peak in SEC (Figure 3).

**Figure 3—figure supplement 1.**
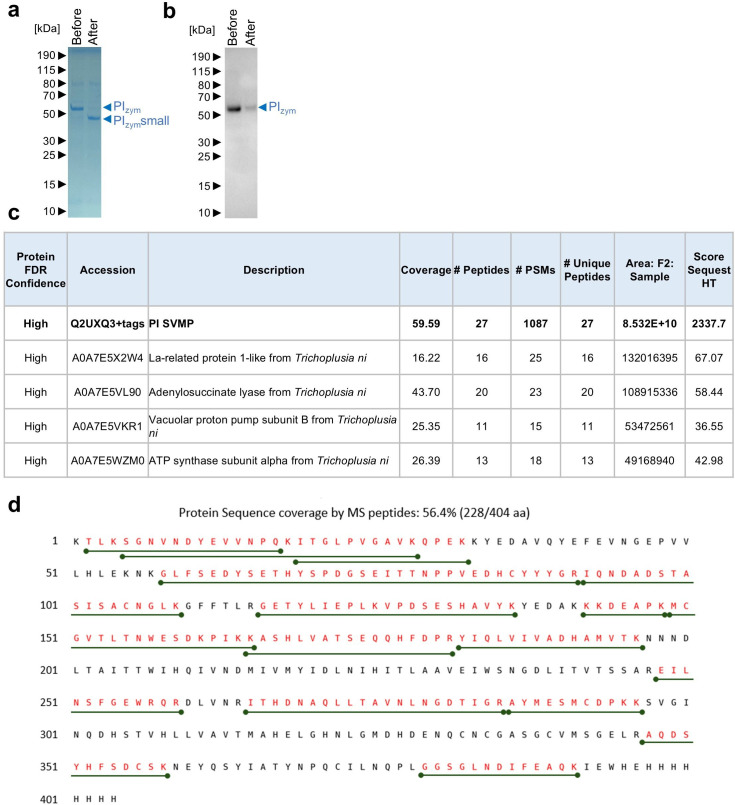
PI snake venom metalloproteinase (SVMP) C-terminal auto-cleavage before and after dialysis, following IMAC purification. (**a**) SDS-PAGE and (**b**) western blot using HRP-conjugated anti-Penta-His antibody of sample before and after dialysis, following IMAC purification. (**c**) Top five LC-MS/MS (liquid chromatography-tandem mass spectrometry) hits of before dialysis sample. Proteins were identified using the Sequest search engine against the UniProtKB *Trichoplusia ni* (Hi5) database containing sequence for the protein of interest (PI SVMP). Keratins and trypsin were excluded as common contaminants. (**d**) PI SVMP protein sequence coverage by LC-MS/MS. Peptides identified by MS that provide unique sequence information are shown as horizontal bars below the amino acid sequence. Residues detected in the analysis are highlighted in red, covering 56% of the protein sequence. Figure 3—figure supplement 1—source data 1.Original files for SDS-PAGE and western blot analysis displayed in Figure 3—figure supplement 1a and b. Figure 3—figure supplement 1—source data 2.PDF file containing original SDS gel and western blot for Figure 3—figure supplement 1a and b, indicating the relevant bands. Figure 3—figure supplement 1—source data 3.Excel sheet comprising the MS results for all peptides identified in the PI SVMP protein sample for Figure 3—figure supplement 1c.

**Figure 3—figure supplement 2.**
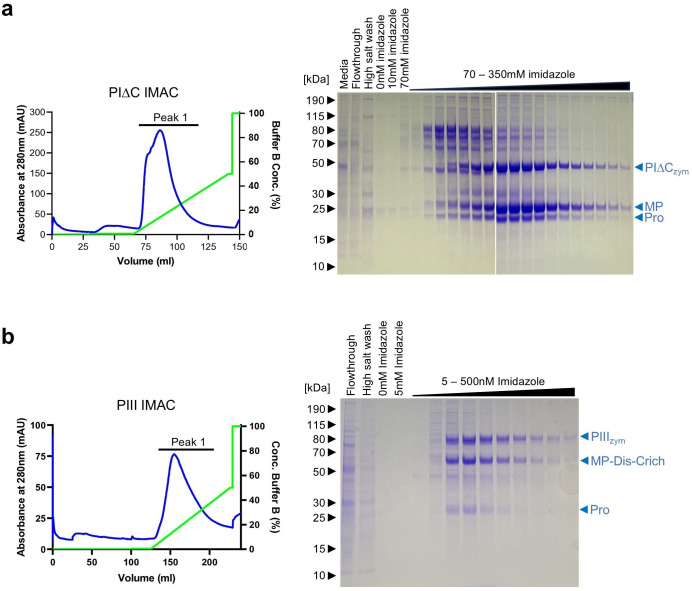
IMAC purification of secreted snake venom metalloproteinases (SVMPs) from insect cell media. Chromatograms and reducing SDS-PAGE of IMAC purification of (**a**) PIΔC and (**b**) PIII SVMP. Figure 3—figure supplement 2—source data 1.Original files for SDS-PAGE analysis displayed in Figure 3—figure supplement 2. Figure 3—figure supplement 2—source data 2.PDF file containing original SDS gels for Figure 3—figure supplement 2, indicating the relevant bands. Figure 3—figure supplement 2—source data 3.Excel sheet comprising the data of the chromatograms of the IMAC purification of PIΔC and PIII SVMP shown in Figure 3—figure supplement 2a and b.

**Figure 3—figure supplement 3.**
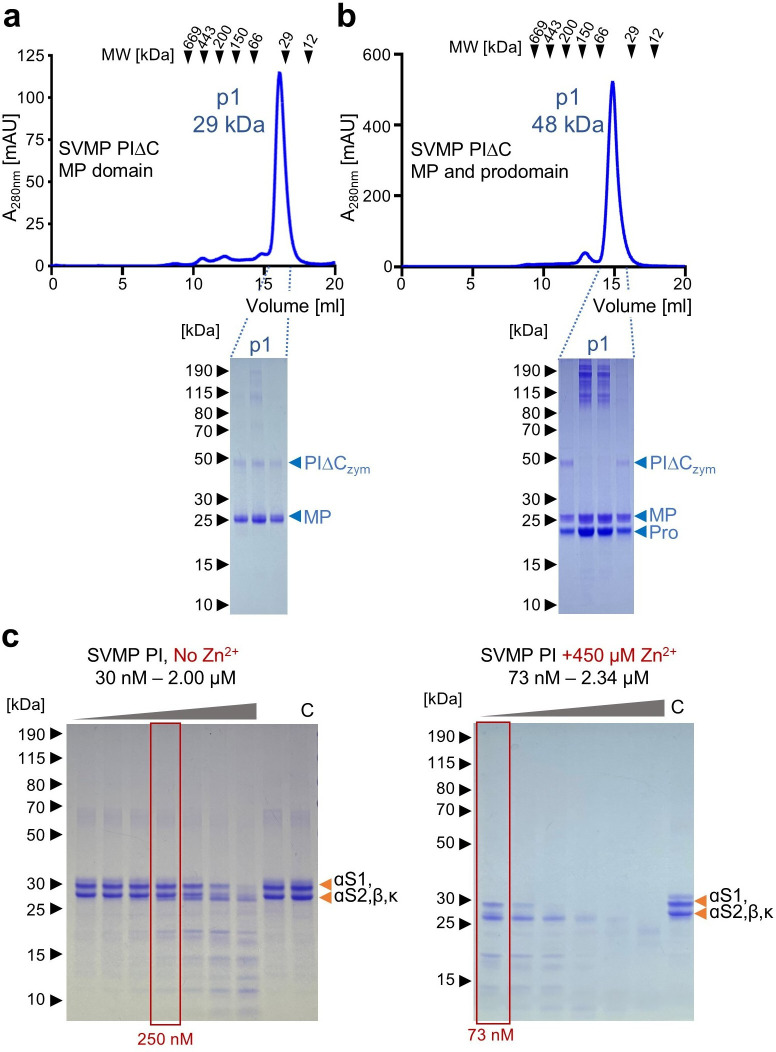
Size exclusion chromatography (SEC) purification and auto-activation of PIΔC snake venom metalloproteinase (SVMP) zymogen, following IMAC and IEX. Size exclusion chromatograms and SDS-PAGE of (**a**) metalloproteinase (MP) domain of PIΔC and (**b**) MP domain and prodomain (Pro) of PIΔC. (**c**) PIΔC activity against casein with no prior addition of Zn^2+^ ions (left) and with prior incubation with 450 μM of Zn^2+^ (right, see also Figure 4a). Titration of PIΔC SVMP (concentration range indicated) into 23 μM casein, followed by incubation at 37 °C for 18 hr. 5 μl reaction run on a reducing SDS-PAGE gel. Figure 3—figure supplement 3—source data 1.Original files for SDS-PAGE analysis displayed in Figure 3—figure supplement 3. Figure 3—figure supplement 3—source data 2.PDF file containing original SDS gels for Figure 3—figure supplement 3, indicating the relevant bands. Figure 3—figure supplement 3—source data 3.Excel sheet comprising the data for the size exclusion chromatograms of PIΔC MP alone and of PIΔC MP with prodomain shown in Figure 3—figure supplement 3a and b.

**Figure 3—figure supplement 4.**
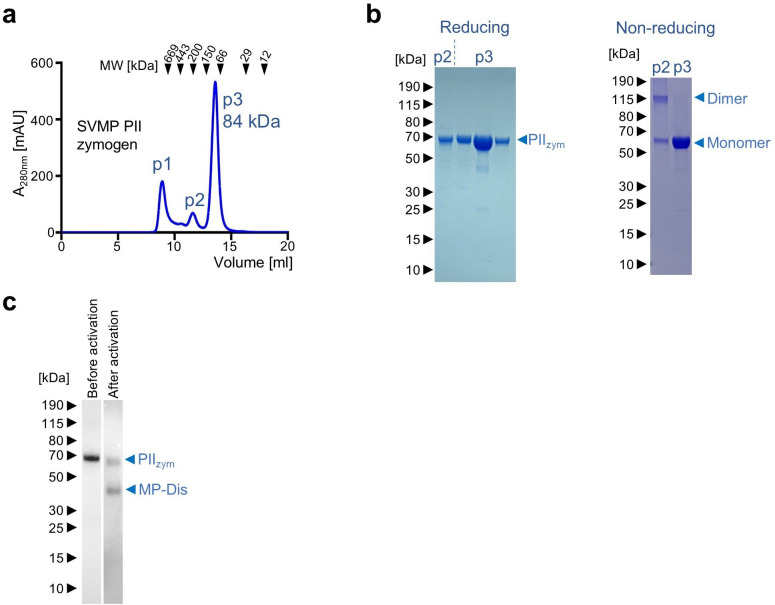
Size exclusion chromatography (SEC) purification of PII snake venom metalloproteinase (SVMP) zymogen, following IMAC and IEX. (**a**) Size exclusion chromatogram and (**b**) reducing and non-reducing SDS-PAGE of SEC peaks 2 and 3. (**c**) Western blot analysis using HRP-conjugated anti-Penta-His antibody of sample before and after activation of PII SVMP. Figure 3—figure supplement 4—source data 1.Original files for SDS-PAGE analysis and western blot displayed in Figure 3—figure supplement 4b and c. Figure 3—figure supplement 4—source data 2.PDF file containing original SDS-PAGE analysis and western blot for Figure 3—figure supplement 4b and c, indicating the relevant bands. Figure 3—figure supplement 4—source data 3.Excel sheet comprising the data for the SEC of PII SVMP zymogen shown in Figure 3—figure supplement 4a.

**Figure 3—figure supplement 5.**
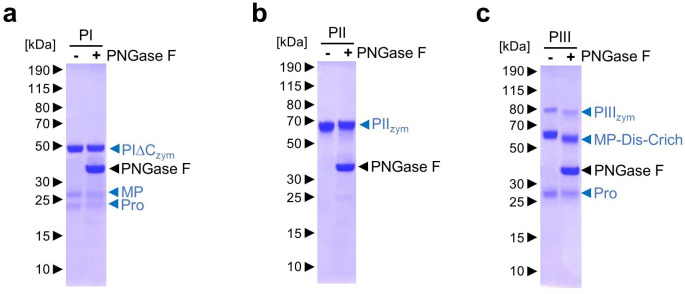
Glycosylation test of recombinant snake venom metalloproteinases (SVMPs). Purified (**a**) PI, (**b**) PII, and (**c**) PIII SVMP zymogens were treated with PNGase F. A shift to lower molecular weight (MW) is observed in bands corresponding to PIII SVMP zymogen and mature PIII SVMP (MP-Dis-Crich) in panel c. Figure 3—figure supplement 5—source data 1.Original files for SDS-PAGE analysis displayed in Figure 3—figure supplement 5. Figure 3—figure supplement 5—source data 2.PDF file containing original SDS-PAGE analysis for Figure 3—figure supplement 5, indicating the relevant bands.

**Figure 3—figure supplement 6.**
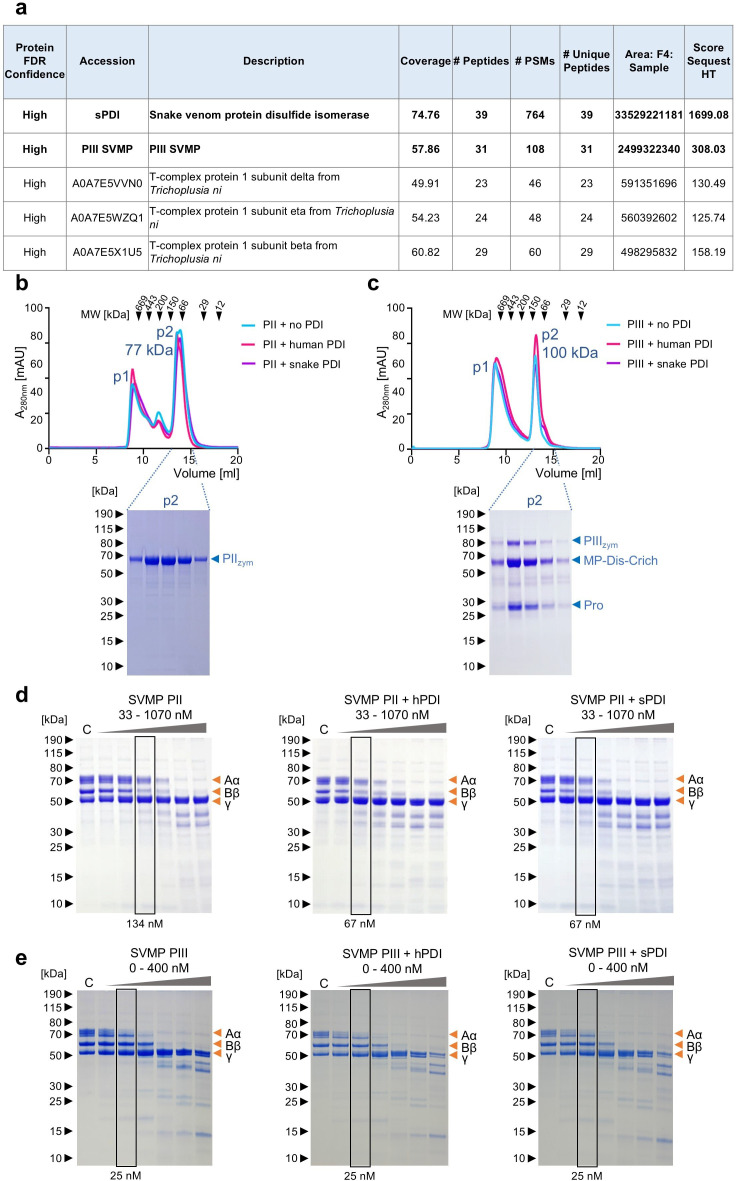
Size exclusion chromatography (SEC) chromatograms of PII and PIII snake venom metalloproteinase (SVMP) zymogens produced in the presence and the absence of human and snake protein disulfide isomerases (PDI). (**a**) Top five LC-MS/MS hits of PIII + sPDI IMAC flowthrough. Proteins were identified using the Sequest search engine against the UniProtKB *Trichoplusia ni* (Hi5) database containing sequences for the proteins of interest (PIII SVMP and snake PDI). Keratins and trypsin have been excluded as common contaminants. (**b**) PII SVMP zymogen and (**c**) PIII SVMP zymogen were co-expressed with human (magenta) or snake (purple) PDIs. SEC chromatograms were aligned with the control where no PDI was expressed (cyan). (**d**) Fibrinogen degradation assays in the presence of Zn^2+^ and increasing amounts of SVMPs PII, PII + hPDI (human PDI) and PII + s PDI (snake PDI). (**e**) Fibrinogen degradation assays in the presence of Zn^2+^ and increasing amounts of SVMPs PIII, PIII + hPDI and PIII + sPDI. Degradation assays were performed four times for the PII SVMP + PDI assays and six times for the PIII SVMP + PDI assays. Figure 3—figure supplement 6—source data 1.Original files for SDS-PAGE analysis displayed in Figure 3—figure supplement 6b–e. Figure 3—figure supplement 6—source data 2.PDF file containing original SDS-PAGE analysis for Figure 3—figure supplement 6b–e, indicating the relevant bands. Figure 3—figure supplement 6—source data 3.Excel sheets comprising all peptides identifiied by LC-MS/MS of PIII + sPDI sample (shown in panel a), and data for size exclusion chromatograms shown in Figure 3—figure supplement 6b and c.

**Figure 3—figure supplement 7.**
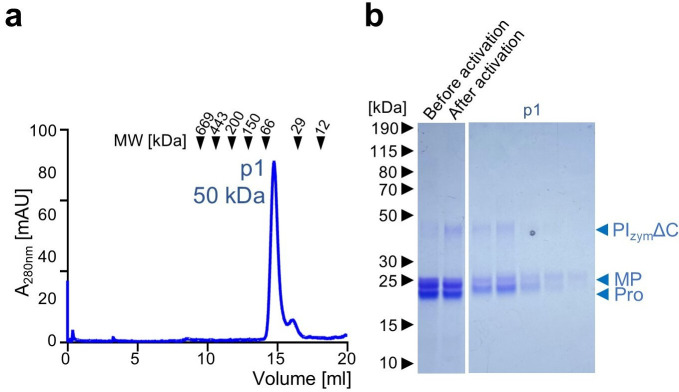
Auto-activated PIzymΔC size exclusion chromatography (SEC). (**a**) Size exclusion chromatogram and (**b**) corresponding Coomassie-stained SDS gel of activated PIzymΔC, confirming metalloproteinase (MP) domain and prodomain (Pro) remain bound together, despite the protein being active. Figure 3—figure supplement 7—source data 1.Original file for SDS-PAGE analysis displayed in Figure 3—figure supplement 7b. Figure 3—figure supplement 7—source data 2.PDF file containing original SDS-PAGE analysis for Figure 3—figure supplement 7b, indicating the relevant bands. Figure 3—figure supplement 7—source data 3.Excel table of sixe exclusion chromatogram of auto-activated PIΔC shown in Figure 3—figure supplement 7a.

Notably, dialysis following IMAC affinity purification of PI SVMP resulted in self-cleavage of the zymogen as evidenced by a downward shift of the PI protein band in the SDS-PAGE gel from 55 kDa to 45 kDa before and after dialysis, respectively (Figure 3—figure supplement 1a). Western blot analysis indicated a loss of the His-tag after dialysis (Figure 3—figure supplement 1b). The protein was confirmed to be PI SVMP by mass spectrometry (Figure 3—figure supplement 1c and d). The observed auto-activation of PI SVMP is likely due to residual metal ions in the insect cell media and buffers, trace metal ions leaching off the resin during IMAC purification, and/or a change in pH from the cell culture medium at pH 6 to the purification buffer at pH 8, which is the pH recommended for binding to the IMAC column – the lower pH of the medium being similar to that of the venom gland (pH 5.4), which helps maintain enzyme latency (Mackessy and Baxter, 2006). The PI SVMP zymogen without the C-terminus (including the Avi-tag and His-tag) eluted from the SEC column in one peak at 14.8 ml, corresponding to a calculated molecular weight (MW) of ~50 kDa which agrees with the predicted MW for the monomeric zymogen (Figure 3a). The yield of purified PI SVMP zymogen was 0.63 mg from a 1 L Hi5 insect cell culture.

Following observation of the loss of the SVMP PI C-terminus during purification, a new protein construct was designed with the Avi- and His-tags fused C-terminally to the observed proteolytic degradation site as suggested by MW loss seen by SDS-PAGE and based on AlphaFold 3 models (Abramson et al., 2024), giving rise to a shorter construct named PIΔC. In fact, this new C-terminus of the MP domain aligns with that of fully characterised PI SVMPs found in venom (Sanchez et al., 2010; Bello et al., 2006; Bernardes et al., 2008). During PIΔC IMAC affinity purification, the zymogen (predicted MW: 45.4 kDa) underwent partial activation, resulting in cleavage between the MP (theoretical MW: 26.2 kDa) and prodomain (theoretical MW: 19.3 kDa; Figure 3—figure supplement 2a). Subsequent IEX allowed for the isolation of a small amount of PIΔC MP domain alone; however, the bulk of the PIΔC MP remained bound to the prodomain, forming a stable complex despite cleavage. The PIΔC MP domain alone eluted from the SEC column at a MW of ~29 kDa, corresponding to the MW expected for the monomeric MP domain (Figure 3—figure supplement 3a). The PIΔC MP in complex with the prodomain eluted from the SEC column in a different peak corresponding to a MW of ~48 kDa, confirming that the cleaved prodomain and MP domain form a stable assembly with a 1:1 stoichiometry (Figure 3—figure supplement 3b). The yield of PIΔC was 0.44 mg for the MP domain and 3.25 mg for the MP with non-covalently bound prodomain from a 1 L Hi5 insect cell culture.

The PII SVMP zymogen was purified as intact zymogen and did not undergo any (self-) cleavage throughout purification (Figure 3b). In SEC, a small amount of protein eluted in the void volume, likely due to the very high concentration of PII zymogen when loaded onto the column, possibly causing some aggregation. The majority of PII SVMP zymogen eluted with a small peak corresponding to ~198 kDa (p2) and a large, sharp peak corresponding to ~84 kDa (p3). SDS-PAGE analysis with non-reducing loading dye suggests that peak p2 corresponds to a PII zymogen dimer (Figure 3—figure supplement 4a and b). Peak p3 corresponds to the monomeric PII SVMP zymogen, which has a predicted MW of 56 kDa, in agreement with its elution volume in SEC. The yield of PII SVMP zymogen was 8.3 mg (peak 3) from a 1 L Hi5 insect cell culture.

The PIII SVMP zymogen underwent partial activation during the purification process, similar to that of PIΔC SVMP. This was observed following elution from the IMAC column (Figure 3—figure supplement 2b) and resulted in the presence of three bands in SDS-PAGE (Figure 3c), corresponding to the PIII zymogen (predicted MW 70.2 kDa), mature PIII SVMP (MP domain, disintegrin-like domain and cysteine-rich domain) with a predicted MW of 50.6 kDa, and the prodomain with a MW of 19.6 kDa. Interestingly, despite the processing of some PIII molecules, the protein still eluted in one peak from the SEC, at a MW of ~108 kDa based on the elution volume. This indicates that the prodomain again remains associated with the mature SVMP following cleavage, as previously observed for the PIΔC SVMP (Figure 3—figure supplement 2a). The yield of the PIII SVMP was 2.2 mg from a 1 L Hi5 insect cell culture.

We observed a difference in the calculated MW based on SEC elution volume (~108 kDa) and the predicted MW (70 kDa) for the PIII SVMP zymogen. Similarly, the PIII zymogen and mature PIII SVMP both run at a higher-than-expected MW on SDS-PAGE (~80 kDa and ~60 kDa, respectively). In order to explain this difference in apparent MW of the PIII SVMP, we analysed the proteins for the presence of N-glycans. The NetNGlyc 1.0 server predicts 2 glycosylation sites in the mature PIII SVMP (Gupta and Brunak, 2002), and we tested all purified SVMP zymogens for glycosylation using Peptide:N-glycosidase F (PNGase F; Figure 3—figure supplement 5). The bands corresponding to PI and PII SVMPs did not change in the presence of PNGase F. However, we observed a downward shift in the MW of the bands corresponding to the PIII zymogen and mature PIII SVMP protein in SDS-PAGE following treatment with PNGase F. De-glycosylation of PIII proteins produced bands at ~77 kDa and ~57 kDa, closer to their predicted MWs (Figure 3—figure supplement 5c). We thus confirmed glycosylation of our recombinant PIII SVMP, accounting for the observed higher MW. Due to unavailability of the corresponding native PIII SVMP from *E. carinatus sochureki*, a more detailed comparative analysis of glycosylation and comparison of glycosylation patterns between native and recombinant PIII SVMP is precluded.

In conclusion, by using the protocol we have developed, we can produce highly purified recombinant SVMP zymogens, each in mg quantities per litre expression culture.

### Co-expression of protein disulfide isomerases does not improve SVMP zymogen yields or folding

Transcriptomic studies have shown that snake venom glands overexpress several chaperones alongside venom toxins, in particular protein disulfide isomerases (PDI), heat shock proteins, and calreticulin (Junqueira-de-Azevedo et al., 2015). These chaperones are expected to be essential for the correct folding of complex, disulfide-rich toxins. In particular, PDIs assist in the formation, breakage, and rearrangement of disulfide bonds, stabilising the proteins. Successful folding of venom peptides aided by PDI was previously demonstrated for marine cone snails (Safavi-Hemami et al., 2016). Furthermore, overexpression of PDI during recombinant protein expression has been shown to increase overall protein yield (Hsu et al., 1996). Therefore, co-expression of SVMPs with PDI could increase SVMP protein yields enabling more protein to be correctly folded and secreted, potentially reducing protein aggregation. To test our hypothesis, we co-expressed the PII and PIII SVMP zymogens with two PDIs: a human PDI (UniProt ID: P07237) and a snake PDI (from *E. ocellatus*; Redureau et al., 2025), the latter is overexpressed in venom gland tissue (Figure 3—figure supplement 6a). Contrary to our expectations, PDI overexpression in insect cells did not noticeably enhance the expression or folding of our SVMP zymogens, and we did not observe increased SVMP yield or activity (Figure 3—figure supplement 6 and Appendix).

### Auto-activation of SVMP zymogens reveals cleavage of the prodomains, the PII disintegrin domain and PIII prodomain degradation

Cleavage of the prodomain in native zymogens is thought to result in the removal of the propeptide from the active site rendering it accessible to substrates, with Zn^2+^ as an essential cofactor. We thus hypothesised that activation of our purified zymogens could be achieved by addition of Zn^2+^ ions. Therefore, we incubated the SVMPs with Zn^2+^ ions at 37 °C for 18 hr and optimised the Zn^2+^ concentration for activation for each SVMP (Figure 3d–f).

The PI SVMP zymogen could be close to completely activated by 750 μM ZnCl_2_ (Figure 3d). When analysed by SDS-PAGE, the ~45 kDa band corresponding to the zymogen mostly disappears, and two lower MW bands of ~23 kDa and ~26 kDa appear, corresponding to the prodomain and MP domain, respectively. The two proteins could not be separated under the native conditions of SEC, indicating that the prodomain remains stably bound to the MP domain, despite cleavage (as observed for PIΔC, Figure 3—figure supplement 3b). Separation of the MP and prodomain has occurred during SDS-PAGE; however, due to the denaturing and thiol-reducing conditions used. AlphaFold 3 generated models (Abramson et al., 2024; Figure 2a) suggest the prodomain is located on the opposite side of the MP domain, distal from the active site. Thus, the bound prodomain is not expected to interfere with the ability of the MP to bind and cleave substrates, once the propeptide has been detached from the active site. It is interesting to note that the C-terminal part of the PI SVMP zymogen was cleaved during buffer exchange by dialysis while the zymogen was still intact (Figure 2—figure supplement 2a) and assumed to be inactive. The observed C-terminal cleavage could be due to a few auto-activated PI SVMPs or due to contaminating proteases from insect cells still present in the affinity purified protein fractions.

The truncated PIΔC SVMP construct was almost completely cleaved into mature protein during purification; however, this protein was not fully active in our assays. For full activity, the PIΔC SVMP was incubated with 450 μM ZnCl_2_ leading to significantly higher activity in casein assays (Figure 3—figure supplement 3c). Despite complete cleavage of the zymogen, the resulting prodomain and MP domain could not be separated, as was the case for the full-length PI, and, thus, no MP was isolated from this construct either (Figure 3—figure supplement 7). Because this PIΔC SVMP corresponds better to the processed, native version of PI SVMP protein, PIΔC was used in all further activity assays and is referred to as PI SVMP from here on.

The PII SVMP zymogen was completely activated by 750 μM ZnCl_2_ (Figure 3e). Notably, when analysed by SDS-PAGE, a clear separation of prodomain and mature protein was not observed. The band migrating at ~40 kDa corresponds to MP and disintegrin (confirmed by western blot, Figure 3—figure supplement 4c). This band disappears with higher concentrations of Zn^2+^ and/or longer incubation times. The band migrating at ~8 kDa is thought to correspond to the processed disintegrin domain but could not be confirmed by western blot due to loss of C-terminal tags during activation. After incubation for 1 week, only the 8 kDa and 23 kDa bands remained (Figure 3e). Based on apparent MW, these bands correspond to the disintegrin and prodomain, respectively. In summary, PII SVMP has undergone further processing to remove the tags and disintegrin from the MP domain, and the MP domain appears to be unstable over time.

The PIII SVMP zymogen was completely activated by the addition of 150 μM ZnCl_2_ (Figure 3f) as evidenced by the disappearance on SDS-PAGE of the ~77 kDa band corresponding to the zymogen. Instead, the band strengthened at ~60 kDa corresponding to the mature SVMP (MP domain plus disintegrin-like domain and cysteine-rich domain) together with a band at ~25 kDa corresponding to the prodomain. Notably, at higher concentrations of Zn^2+^, the C-terminal tags and the prodomain band are degraded by the PIII SVMP.

We conclude that all SVMP zymogens studied can auto-activate by incubation with Zn^2+^, leading to cleavage of the prodomain from the zymogen. In the case of PIΔC and PIII SVMPs the prodomain remains associated with the SVMP at low Zn^2+^ concentrations. After Zn^2+^ activation, the C-terminal tags and the PIII prodomain are degraded by the activated SVMPs, and in the case of PII SVMP, further proteolysis occurs into the constituent parts.

### Casein and fibrinogen degradation assays demonstrate SVMP activity

**Figure 4.**
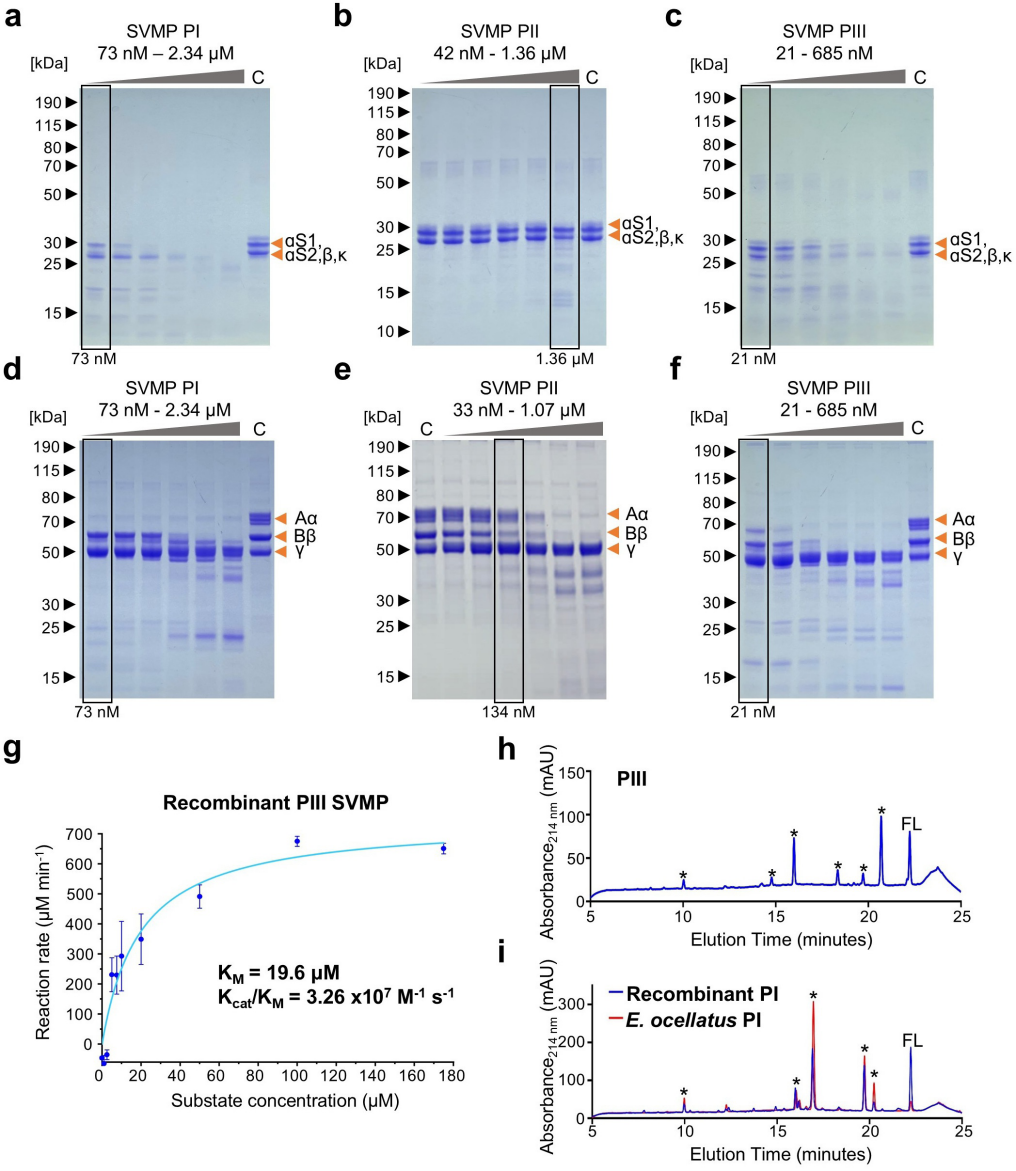
*In vitro* snake venom metalloproteinase (SVMP) activity and substrate specificity. Casein degradation in the presence of increasing amounts of (**a**) PI, (**b**) PII, and (**c**) PIII SVMPs. Orange arrowheads: domains αS1, αS2, β, and κ. Fibrinogen degradation in the presence of increasing amounts of (**d**) PI, (**e**) PII, and (**f**) PIII. Orange arrowheads: fibrinogen chains Aα, Bβ, and γ. C (control): incubation in the presence of 5 mM EDTA. (**g**) PIII SVMP (20 nM) activity towards the fluorogenic peptide substrate ES010. (**h, i**) Degradation of insulin B by (**h**) PIII and (**i**) recombinant (blue) and *Echis ocellatus* PI (red). Full-length (FL) insulin and degradation products (*) are marked above the peaks. All degradation assays were repeated three times. The fluorogenic peptide assay shows the result of three independent repeats. Figure 4—source data 1.Original files for original SDS-PAGE gels displayed in Figure 4a–f. Figure 4—source data 2.PDF file containing original SDS-PAGE gels for Figure 4a–f, indicating the relevant bands. Figure 4—source data 3.Excel sheets comprising the data for the fluorogenic peptide substrate ES010 degradataion assay using PIII SVMP (g) and the data for the RP-HPLC runs of insulin B degradation by PIII SVMP (h) and by recombinant and native PI SVMP (i) shown in Figure 4g, h and i.

Degradation of casein is a general protease activity assay that is used in functional assays for all SVMP classes (Macêdo and Fox, 2016). SVMP zymogens were supplemented with optimal concentrations of ZnCl_2_ for SVMP activation (Figure 3d–f) and incubated with 0.525 μg/μl (~23 μM) casein overnight at 37 °C. The degradation of casein was analysed by SDS-PAGE; indicated concentrations relate to input SVMP zymogen concentrations (Figure 4a–f). The PI SVMP degraded casein (Figure 4a); activity was observed when adding 73 nM PI and complete degradation of casein occurred at the addition of 1.17 μM PI. The PII SVMP exhibited weak activity against casein (Figure 4b), which may be due to the self-degradation of the MP domain observed during Zn^2+^ activation (Figure 3e). Casein degradation was observed when adding 1.36 μM PII, as evidenced by the emergence of lower MW bands (Figure 4b). The PIII SVMP was active against all casein chains (Figure 4c), with activity seen from concentrations as low as 21 nM PIII, and complete digestion of casein was observed with 343 nM PIII.

**Figure 4—figure supplement 1.**
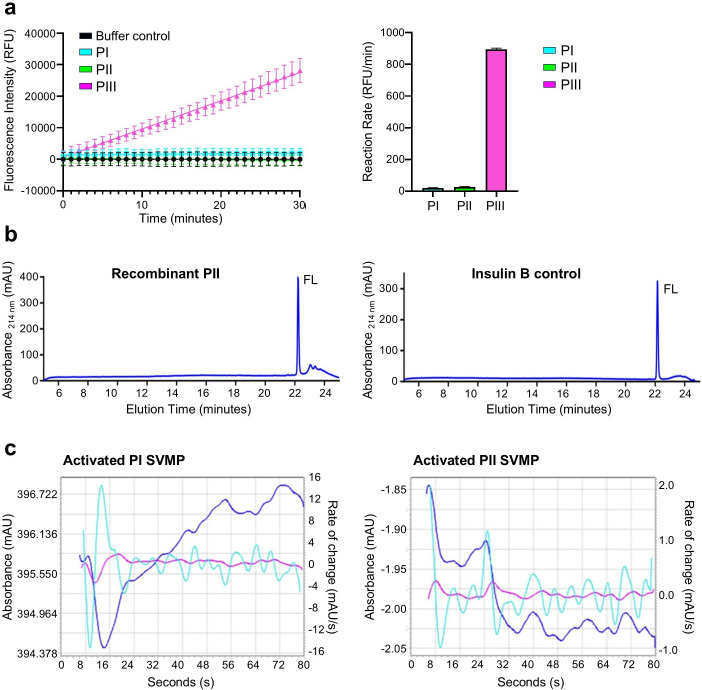
Snake venom metalloproteinase (SVMP) activity assays and blood coagulation tests. (**a**) PI, PII, and PIII SVMP activity (40 nM each) towards the fluorogenic peptide substrate ES010 (100 µM). Data points represent the mean of four individual values recorded over two independent technical replicates, and error bars represent SD. The rate of reaction was determined by calculating the slope of the fluorescence intensity versus time curve.(**b**) Degradation of insulin B by PII SVMP (above) and insulin B only (negative control, below). Full-length (FL) insulin is marked. See Figure 4h and i PI and PIII SVMP degradation tests, respectively. (**c**) Citrated human blood was spiked with the recombinant PI SVMP (left) and the recombinant PII SVMP (right). Change in absorbance (cyan) is plotted on the left Y axis, rate of change (first derivative: blue; second derivative: pink) is plotted on the right Y axis. See Figure 5d for PIII SVMP for human blood coagulation test. Figure 4—figure supplement 1—source data 1.Excel sheets comprising the data for the fluorogenic peptide substrate ES010 degradataion assays using PI and PII SVMPs (a), the data for the RP-HPLC runs of insulin B degradation by PII SVMP and the insulin B control (b), and the data for the blood clotting experiments with PI and PII SVMPs (c) shown in Figure 4—figure supplement 1a–c.

Next, we performed fibrinogen degradation assays followed by SDS-PAGE analyses (Figure 4d–f). None of our recombinant SVMPs had very strong activity against the γ chain of fibrinogen, as is usually the case with SVMPs (Bernardes et al., 2008). However, PI, PII, and PIII SVMPs degraded Aα and Bβ chains of fibrinogen to different extents.

The majority of PI SVMP activity was directed against the Aα chain of fibrinogen (Figure 4d), with the lowest amount of enzyme tested (73 nM). At higher concentrations of PI (585 nM), activity towards the Bβ chain was also observed. Therefore, PI SVMP can be classified as an alpha/beta fibrinogenase, with a preference for the Aα chain. PII SVMP caused removal of fibrinopeptide B (FpB) from the Bβ chain (Figure 4e) at a concentration of 134 nM. At higher concentrations (268 nM or more), the Aα chain was also degraded. Thus, PII SVMP is an alpha/beta fibrinogenase with a preference for the Bβ chain. PIII SVMP degraded the Aα chain of fibrinogen at the lowest concentration of PIII tested, 21 nM (Figure 4f). When supplementing 85 nM PIII or more, the band at 60 kDa corresponding to the Bβ chain disappears as well. Thus, we classify PIII SVMP as an alpha/beta fibrinogenase, with a preference for the Aα chain.

To summarise, all recombinant SVMPs (PI, PII, and PIII) degraded casein, confirming their activity. They also degraded the alpha and beta chains of fibrinogen in a fibrinogenolytic manner, rather than cleaving fibrinopeptides in a thrombin-like manner.

### Auto-activated PIII SVMP shows high substrate affinity and catalytic efficiency

We next tested if the SVMPs cleave a quenched fluorogenic substrate, ES010 (R&D Systems) which is commonly used to study SVMPs (Nguyen et al., 2022) and matrix MPs (Azevedo et al., 2016) for small molecule therapeutic discovery. PIII SVMP was able to cleave the Gly-Leu bond in ES010 (Figure 4g). In contrast, SVMPs PI and PII did not effectively cleave this PIII SVMP and MMP-prototypic substrate under the conditions tested (Figure 4—figure supplement 1a). For the PIII SVMP, the rate of reaction (monitored by fluorescence increase) was plotted against varying substrate concentrations (ranging from 0 μM to 180 μM), and a Michaelis-Menten curve was calculated (Figure 4g). The low K_M_ of 19.6 μM indicates a high binding affinity of the PIII SVMP for the ES010 fluorogenic substrate. The high catalytic efficiency (k_cat_/K_M_) of 3.26 x 10^7^ M^–1^s^–1^ further suggests that the PIII SVMP is highly effective and specific in processing this substrate. For comparison, a k_cat_/K_M_ value of 4471 M^–1^s^–1^ was reported for matrix metalloproteinase MMP10 for an ESO10-derived peptide (Schlage et al., 2015), and k_cat_/K_M_ values of 30,000 M^–1^s^–1^ for a triple-helical peptide for MMP-2 (Akers et al., 2012). Together, these parameters demonstrate that the recombinant PIII SVMP exhibits both strong substrate affinity and high catalytic efficiency towards this prototypical PIII SVMP substrate.

### Native and recombinant PI SVMP exhibit the same substrate specificity in insulin B degradation assays

Next, we assayed the activity of SVMPs PI, PII, and PIII against insulin B, a test substrate used previously in SVMP characterisation (Wilkinson et al., 2024). Degradation of insulin B at multiple sites was visualised by RP-HPLC. PI and PIII showed distinct insulin B degradation patterns indicating different sequence specificities and cleavage sites while the PII SVMP did not degrade insulin B (Figure 4—figure supplement 1b). PIII SVMP cleaves insulin B into six products (Figure 4h). PI SVMP cleaves insulin B into five products (Figure 4i). While we could not purify the native PII and PIII SVMPs from *Echis* venom for comparative purposes, the native PI SVMP can be readily purified from *E. ocellatus* venom (Howes et al., 2003). Therefore, we could compare insulin B cleavage by our recombinant PI SVMP with insulin B cleavage by the native source-purified PI enzyme. Identical degradation products of insulin B were observed (Figure 4i). Thus, our recombinant PI SVMP is active and has the same substrate specificity as the native enzyme from venom, underscoring that our recombinant expression strategy leads to native-like SVMPs, compellingly validating our approach.

### PII zymogen and activated PII SVMP inhibit platelet aggregation

**Figure 5.**
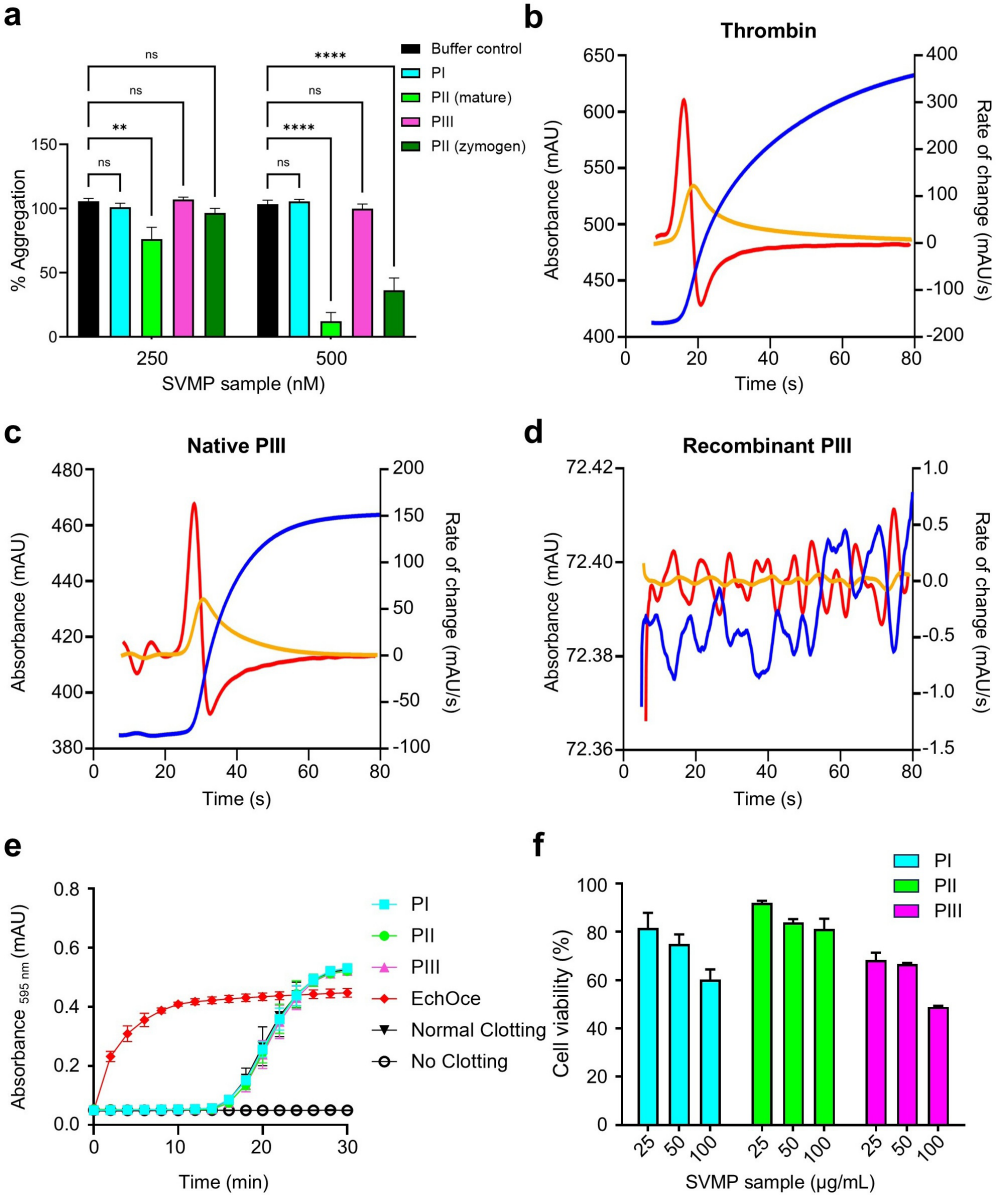
Snake venom metalloproteinase (SVMP) blood coagulation, platelet aggregation, and cytotoxicity. (**a**) Inhibition of platelet aggregation by PI, PII (mature and zymogen), and PIII. Control: PRP with buffer. Data are presented as the mean of % aggregation  ± SEM. A two-way ANOVA was conducted, followed by simple effects analysis (Dunnett post-test) to compare column means (SVMPs) within each row (concentrations). ****: p≤0.0001, **: p≤0.01. The assay was carried out in experimental duplicate and biological triplicate. (**b-d**) Thrombin clot time assays using the ACL TOP coagulation analyser. Citrated human blood was spiked with (**b**) thrombin, (**c**) a pool of native *Echis ocellatus* PIII SVMPs, and (**d**) the recombinant PIII SVMP. Change in absorbance (blue) is plotted on the left Y axis, and rate of change (first derivative: orange; second derivative: red) is plotted on the right Y axis. Assays were repeated three times. (**e**) Coagulation profiles of the recombinant SVMPs and *E. ocellatus* venom in bovine plasma over 30 min at an absorbance of 595 nm. Data points represent the mean of three individual values ± SD. The experiment was repeated two times. (**f**) MTT (3-(4,5-dimethylthiazol-2-yl)–2,5-diphenyltetrazolium bromide) cytotoxicity assay showing viability compared to the buffer-only control of HaCaT cells after 24 hr incubation with activated PI, PII, and PIII SVMPs. Bars show the results of two independent repeats. Figure 5—source data 1.Excel sheets comprising the original data used to generate graphs shown in Figure 5a–f.

Some haemorrhagic SVMPs can disrupt cell adhesion and prevent haemostasis by interfering with platelet aggregation (Takeda et al., 2012). SVMPs with either a disintegrin (PII) or disintegrin-like domain (PIII) can inhibit platelet aggregation through a motif found in these domains. Most commonly, this corresponds to a RGD sequence, which binds integrins. Binding of disintegrin to integrin α_IIb_β_3_ can be measured through inhibition of 2-MeS-ADP-induced platelet aggregation whereby turbidity is determined by plate aggregometry (Figure 5a). When tested at a concentration of 500 nM SVMP, platelet aggregation was reduced to 12% and 36% by the activated PII SVMP and the zymogen form, respectively, confirming that the disintegrin is active and capable of inhibiting platelet aggregation. Based on our results, the presence of the prodomain in the zymogen has low inhibitory effect on the RGD-containing disintegrin domain, as anticipated. In comparison, the PI and PIII SVMPs did not inhibit 2-MeS-ADP-induced platelet aggregation (Figure 5a). The PI SVMP lacks the disintegrin domain and instead of an RGD motif, the PIII SVMP has a divergent ‘RSECD’ motif in its disintegrin-like domain, which most likely does not interact with integrin α_IIb_β_3_. We conclude that our recombinant PII SVMP inhibited platelet aggregation, confirming disintegrin activity in the context of both the zymogen and also the activated PII SVMP.

### PIII SVMP has no pro-coagulant activity in thrombin clot time assays

Thrombin clot time assays measure the time required for thrombin to convert fibrinogen to fibrin, leading to clot formation. This assay is used in the clinic to highlight abnormalities in fibrinogen and fibrin clot formation. In our assays, thrombin was replaced with each SVMP to identify the thrombin-like activity of the enzyme. The assay measures turbidity, which is plotted against time. The first and second derivatives are plotted on the same graph, and the maximum rate of change of turbidity (the peak in the second derivative) indicates the time of clot formation. When thrombin is added, fibrin clot formation requires 16.5 s (Figure 5b). As a positive control and example of pro-coagulant SVMPs, thrombin was replaced with native PIII SVMPs purified from *E. ocellatus* venom. Fibrin clot formation occurs in 28.1 s in the presence of the *E. ocellatus* PIII SVMPs (Figure 5c), confirming clot formation and pro-coagulation activity. However, when replaced with the recombinant PIII SVMP (from *E. carinatus sochureki*) no clot formation occurs (Figure 5d). Similarly, PI and PII SVMPs do not cause blood clotting (Figure 5e, Figure 4—figure supplement 1c). This shows that our recombinantly produced SVMPs have no pro-coagulant activity, which was reported previously for the PI SVMP (Howes et al., 2003) but remained unknown for the PII and PIII SVMPs.

### PI and PIII SVMPs are cytotoxic

HaCaT cells (immortalised human keratinocyte cells), commonly used in dermatological, toxicological, and cancer research, were incubated with PI, PII and PIII SVMPs for 24 hr, and cytotoxicity was monitored using the MTT (3-(4,5-dimethylthiazol-2-yl)–2,5-diphenyltetrazolium bromide) assay (Mosmann, 1983). At the tested concentration, PII SVMP showed minimal cytotoxicity (Figure 5f). In comparison, at 100 µg/ml the PI and PIII SVMPs were clearly cytotoxic, with cell viability decreasing by 40% and 50% after 24 hr, respectively (Figure 5f). The cytotoxic effect of our PIII SVMP (*E. carinatus sochureki*; 100 µg/ml) is comparable to that of crude *E. carinatus* venom on HEK 293T cells, which induces 63% cytotoxicity with 100 µg/ml venom after 3 hr (Pierce et al., 2011). Published IC_50_ values for HaCaT cells treated with whole venom for 24 hr fall between 10 and 100 µg/µl (Hall et al., 2023); however, it is important to highlight the distinction between the complex cocktail of toxins found in venom and the homogenous, purified SVMP evaluated in this study.

## Discussion

The characterization of SVMP toxins, critical for effective bioprospecting and the discovery of snakebite therapeutics, is severely hampered by difficulty in isolating the native proteins from venom. Here, we established a generic protocol for efficient recombinant production of the three structurally diverse classes of SVMPs as zymogens, which overcomes this obstacle. We demonstrate expression of PI, PII and PIII SVMP zymogens from *Echis* species in high quality and quantity using the MultiBac baculovirus/insect cell expression system.

In SVMP zymogens, the highly conserved propeptide sequence which blocks the active site of the MP domain, is clamped in place by the N-terminal prodomain. It should be noted that despite expression as a zymogen, residual cytotoxicity was still observed, evident by the decrease in cell viability throughout expression and reduction in the amount of co-expressed YFP fluorescence due to cell lysis (Figure 2). In agreement with this observation, PI and PIII SVMPs were able to partially activate themselves, resulting in the presence of three characteristic bands in Coomassie-stained SDS gels – the zymogen, mature SVMP, and prodomain. We speculate that this auto-cleavage could be due to the SVMP active site accreting divalent metal ions from the media and buffers used. Additionally, it is likely that the change in pH from 6 (expression) to 8 (purification) allows the activation of SVMPs, as is the case when venom is released from the venom gland (pH 5.4) into the victim (pH 7.4) during envenoming (Mackessy and Baxter, 2006). A complete control of self-activation of SVMP zymogens would be highly desirable. However, previous studies reported that (i) SVMP zymogens are processed within secretory cells of the venom gland (Portes-Junior et al., 2014), and (ii) mature SVMPs accumulate in secretory vesicles during venom production (Carneiro et al., 2002). Accordingly, complete prevention of SVMP zymogen auto-processing will be very difficult to achieve because this would require Zn^2+^ ion depletion within the cells which would result in cytotoxicity.

Of note, despite the self-cleavage of the PI and PIII zymogens, the majority of the mature SVMP protein remained associated with the prodomain. Interestingly, this did not affect the activity of the SVMPs. According to AlphaFold 3 structure predictions, the prodomain is located on the side of the MP domain, which is opposite to the active site. Upon activation, cleavage of the prodomain releases the propeptide from the active site. This renders the Zn^2+^ ion in the active site free to interact with a substrate. In agreement, we observe that the SVMPs are active even if the prodomain, now no longer covalently linked to the SVMP, remains bound to the MP domain (Figure 3c, Figure 3—figure supplement 2).

SVMP zymogens undergo additional post-translational processing: The PI SVMP construct described here was based on transcriptomic data and corresponds to the UniProt sequence ID: Q2UXQ3. During purification, it underwent C-terminal truncation by ~10 kDa, leading to a smaller protein which corresponds closely to previously characterised PI SVMPs (Sanchez et al., 2010; Bello et al., 2006; Bernardes et al., 2008). Moreover, we observed a general trend of loss of C-terminal tags during purification, activation, and subsequent activity assays for all three SVMP classes.

Here, we show that the recombinant PIII SVMP comprises N-linked glycosylation. We would like to point out that when expressed in insect cells, the glycosylation pattern may be different compared to endogenous SVMPs due to differences in the snake and insect glycosylation pathways. The Sf21 and Hi5 cells used in this study typically produce N-glycans that are trimmed to a core ‘paucimannose’ structure (Man3GlcNAc2), often with an alpha1,6-fucosylation (Watanabe et al., 2002). While snakes can produce more complex, sialylated N-glycans, glycomic studies of native venoms (e.g. *Bothrops* venoms) have demonstrated that high-mannose and paucimannose structures are also prevalent in native SVMPs (Andrade-Silva et al., 2018). Therefore, the recombinant glycoforms likely represent a subset of the native glycan heterogeneity and not the full diversity found in snake venom toxins. Moreover, Sf21 and Hi5 cells recognise the same N-glycosylation sequon (Asn-X-Ser/Thr) as reptilian cells, and therefore the site-occupancy remains consistent with the native protein, preserving the overall topography of the toxin.

To improve SVMP yields and/or activity, we hypothesised that co-expression of SVMPs with human or snake PDI would aid their proper folding, ensuring the multiple disulfide bonds were correctly formed. Based on transcriptomic data from *Echis* venom gland (Redureau et al., 2025), PDIs are highly overexpressed in the snake venom gland. However, PDI over-expression in insect cells did not improve protein yields nor enzyme activity. We speculate that the endogenous insect cell PDIs are sufficient for SVMP folding in our setup. Alternatively, a combination of different chaperones may be required for further boosting SVMP production.

SVMP zymogens from all three classes could be auto-activated by the addition of Zn^2+^ ions. While some PI SVMP was processed during purification, any remaining PI zymogen could be cleaved into the mature SVMP and prodomain by incubation with Zn^2+^. The PII zymogen could be activated by the same means, although there was no clear separation into prodomain and mature protein. Instead, we observed two bands corresponding to the disintegrin and prodomain after prolonged incubation with Zn^2+^ (Figure 3e). It is unlikely that the observed disappearance of the MP domain is due to misfolding because the PII zymogen is well expressed with high yields (9–10 mg/l culture) in insect cells and eluted in two clean peaks in SEC corresponding to a monomer and dimer, respectively. In the case of misfolding, we would have expected aggregation and/or degradation during purification, and we would not expect to see degradation of the zymogen in SDS-PAGE analysis only after addition of Zn^2+^ which activates the MP (Figure 3e). Notably, the disintegrin domain of this PII has 100% sequence identity to ocellatusin, a short, monomeric disintegrin of *E. ocellatus* venom (Smith et al., 2002). Previous studies have identified a discrepancy between the amounts of ocellatusin and its PII-derived MP in the venom, whereby ocellatusin represents 3.9%, and the SVMP less than 0.1% of venom proteins (Wagstaff et al., 2009). In summary, the disintegrin is likely liberated from this PII SVMP and is the more pathogenic component of the SVMP, causing inhibition of platelet aggregation (Figure 5a). In contrast, the PII-derived MP domain is unstable and therefore requires high toxin concentrations to elicit proteolytic activity in our activity assays (Figure 4b and e).

The recombinant PIII zymogen was fully auto-activated in our experiments by the addition of Zn^2+^ ions (Figure 3f). Interestingly, this PIII SVMP was able to degrade its prodomain despite the latter initially remaining bound to the mature protein according to SEC (Figure 3c). This agrees with the finding that prodomains, which are highly conserved in SVMPs, are rarely identified in the venom proteome (Moura-da-Silva et al., 2016), suggesting that all prodomains might eventually be fully degraded by venom proteases including PIII SVMPs.

Activated recombinant SVMPs were active against bovine casein, a general protease substrate (Figure 4a–c). PI and PIII exhibited strong activity, degrading all bands, while PII was significantly less active, requiring much higher enzyme concentrations. This agrees with our previous observation that bands corresponding to the MP-disintegrin protein or the MP domain alone after activation with Zn^2+^ (Figure 3e) are absent in SDS-PAGE, indicating that the MP domain is likely unstable.

Fibrinogen degradation assays are particularly relevant for SVMPs and indicate their potential haemotoxic activity (Figure 4d–f). The 340 kDa fibrinogen glycoprotein is made up of three pairs of polypeptide chains, which lead to three groups of distinct bands when analysed by reducing SDS-PAGE: the Aα (70–75 kDa), Bβ (65 kDa), and γ (50 kDa) chains. In contrast to the endogenous serine protease thrombin, which cleaves fibrinopeptides A and B off the Aα and Bβ chains, causing self-polymerisation into fibrin fibres and formation of blood clots (Weisel and Litvinov, 2017), the PI, PII, and PIII SVMPs studied here caused a fuller degradation of fibrinogen (Figure 4d–f). All SVMPs were active against both the Aα and Bβ chains of fibrinogen; the PI and PIII showed a preference for the Aα chain and the PII preferentially degraded the Bβ chain of fibrinogen. We conclude that the three SVMPs interfere with clot formation and would thus contribute to an anticoagulant outcome upon envenoming. In agreement, the PIII SVMP cannot cause clot formation in the thrombin clot time assay (Figure 5d), suggesting it is not an Ecarin-like prothrombin activator, which is typically responsible for procoagulant activity seen in such assays with *Echis* venoms (Figure 5c and e).

The recombinant PIII SVMP was highly active against a fluorogenic peptide substrate, ES010, showing high affinity and specificity for cleavage of the Gly-Leu bond within the peptide (Figure 4g). In this context, we would like to point out that enzyme concentrations and kinetics are based on zymogen concentrations, assuming 100% SVMP activation by Zn^2+^ (Figure 4a–g). If zymogen activations were incomplete or non-linear, our k_cat_/K_M_ calculations could either underestimate or overestimate the PIII SVMP’s catalytic efficiency (Figure 4g).

The SVMP PIII was also active against insulin B (Figure 4h), as was SVMP PI (Figure 4i) which produced identical degradation products to native PI, confirming identical cleavage sites and successful recombinant production of toxin with native-like characteristics, validating our approach.

In addition to the proteolytic activity of the MP domain, PII and PIII SVMPs also include a disintegrin or disintegrin-like domain containing a potential integrin binding motif. In platelet aggregation assays, our RGD-containing PII SVMP inhibited 2-MeS-ADP-induced platelet-aggregation with and without the prodomain present (Figure 5a). The RSECD-containing PIII, in contrast, did not inhibit 2-MeS-ADP-induced platelet aggregation, but this does not exclude other disintegrin-like activity of this domain.

Zymogen expression was previously reported for ADAMs (A Disintegrin and Metalloproteinases) and MMPs (matrix MPs), which are structurally and functionally related to SVMPs. ADAMs and MMPs are involved in cell-cell interactions, ectodomain shedding, and degradation of extracellular matrix components, respectively (Takeda, 2016). ADAMs and MMPs have become prominent targets in drug discovery (Overall and López-Otín, 2002; Craig et al., 2015). For MMPs, zymogen expression in mammalian and insect cells has been reported and enzyme activation was achieved via 4-aminophenylmercuric acetate or trypsin to remove the prodomain (Ogata et al., 1995; Vallon et al., 1997). Moreover, ADAMs have been expressed with their prodomain to aid proper folding and latency (Jones et al., 2010; Chavaroche et al., 2014). The ADAMs prodomain has been removed post-translationally, for example by co-expression of furin (Wong et al., 2015). Interestingly, in agreement with our PI and PIII SVMP observations, ADAM33 zymogen was processed and secreted in *Drosophila* S2 cells, with the prodomain remaining associated with the catalytic domain after purification. The processing was enhanced by addition of cadmium chloride and zinc chloride (Prosise et al., 2004). Auto-activation of zymogens by Zn^2+^ incubation alone, however, has not been reported to the best of our knowledge.

While ADAMs and MMPs are well characterised in the literature, comparable insights into SVMPs were largely absent to date, due to lack of means for recombinant SVMP protein production. Our generic protocol for recombinant SVMP zymogen production of all three classes overcomes their high cytotoxicity and allows, for the first time, to study auto-activation revealing important differences between the SVMP classes. Furthermore, our protocol will unlock the production of many additional hitherto inaccessible SVMPs, allowing in-depth characterisation of their post-translational processing, enzymatic activity, substrate specificity, cyto- and haemotoxicity, and enable the evaluation of potential biomedical applications to develop new diagnostics and treatments for blood clotting disorders. Our study paves the way to engineer existing and new SVMPs for improved and novel applications in haematology, thrombosis, and inflammation research and other important areas in which SVMPs can play a crucial role. Importantly, the recombinantly produced, tagged SVMPs can serve as antigens for the future selection and evolution of neutralising antibodies as a basis for next-generation snakebite treatment to combat snakebite envenomation.

## Materials and methods

**Key resources table:** 

Reagent type (species) or resource	Designation	Source or reference	Identifiers	Additional information
Gene (*Echis ocellatus*)	Group I snake venom metalloproteinase	UniProt	UniProt ID: Q2UXQ3	
Gene (*E. ocellatus*)	Venom metalloprotease PII-SVMP EOC00006	UniProt	UniProt ID: A0A3G1E3U2	
Gene (*Echis carinatus sochureki*)	*E. carinatus sochureki* Metalloproteinase	UniProt	UniProt ID: E9JG34	
Gene (*Homo sapiens*)	Human protein disulfide-isomerase	UniProt	UniProt ID: P07237	
Strain, strain background (*Trichoplusia ni*)	Hi5 insect cells (BTI-TN-5B1-4)	Thermo Fisher Scientific	Cat number: B85502	Insect cell lines for recombinant protein production
Strain, strain background (*Spodoptera frugiperda*)	Sf21 insect cells (IPLB-Sf-21-AE)	Expression systems	Cat number: 94–003 F	Insect cell lines for recombinant protein production
Genetic reagent (plasmid)	pACEBac1	Geneva Biotech		Baculovirus transfer plasmid used in the MultiBac system
Genetic reagent (plasmid)	pIDC	Geneva Biotech		Baculovirus transfer plasmid used in the MultiBac system
Cell line (*Escherichia coli*)	DH10 with EMBacY	Geneva Biotech		Modified baculovirus genome maintained in DH10 strain
Cell line (*H. sapiens*)	HaCaT	AddexBio	Cat number: T0020001 RRID:CVCL_0038	Cells derived from a 62-year-old Caucasian male donor’s skin epidermis
Biological sample (*E. ocellatus*)	*Echis ocellatus* venom	Liverpool School of Tropical Medicine		Venom extracted from specimens held at the herpetarium facility.
Biological sample (*Bos taurus*)	Citrated bovine plasma	Biowest	Cat number: S0260-500	
Antibody	Rabbit Monoclonal anti-PDI antibody	Cell Signalling Technologies	Cat number: 3501	diluted 1:1,000 in PBS
Antibody	IRDye 680RD Goat Polyclonal Anti-Rabbit IgG	LICORbio	Cat number: 926–68071	diluted 1:10,000 in PBS
Recombinant DNA reagent	PI	Genscript		Synthesised and inserted into the plasmid pACEBac1. Codon optimised for *Spodoptera frugiperda*.
Recombinant DNA reagent	PII	Genscript		Synthesised and inserted into the plasmid pACEBac1. Codon optimised for *Spodoptera frugiperda*.
Recombinant DNA reagent	PIII	Genscript		Synthesised and inserted into the plasmid pACEBac1. Codon optimised for *Spodoptera frugiperda*.
Recombinant DNA reagent	hPDI	Genscript		Synthesised and inserted into the plasmid pIDC. Codon optimised for *Spodoptera frugiperda*.
Recombinant DNA reagent	sPDI from *E. ocellatus*	Genscript		Synthesised and inserted into the plasmid pIDC. Codon optimised for *Spodoptera frugiperda*.
Peptide, recombinant protein	PNGase F	Promega	Cat number:V4831	
Commercial assay or kit	Penta-His HRP Conjugate Kit	QIAGEN	Cat number: 34460	diluted 1:2000 in PBS
Commercial assay or kit	SuperSignal West Pico PLUS Chemiluminescent Substrate	Thermo Scientific	Cat number: 34580	
Commercial assay or kit	Gel Filtration HMW Calibration Kit	Cytiva	Cat number: 28403841	
Commercial assay or kit	Gel Filtration LMW Calibration Kit	Cytiva	Cat number: 28403842	
Commercial assay or kit	Thrombin Time	HemosIL	Cat number: 0009758515	
Commercial assay or kit	MTT Cell Viability Assay Kit	Biotium	Cat number: 30006	
Chemical compound, drug	Marimastat	APExBIO	Cat number: A4049	
Chemical compound, drug	2-MeS-ADP	Tocris	Cat number: 1624	
Chemical compound, drug	Mca-KPLGL-Dpa-AR-NH2 Fluorogenic Peptide Substrate	R&D systems Inc	Cat number: ES010	
Software, algorithm	AlphaFold 3	ColabFold	RRID:SCR_028034	Protein 3D structure prediction
Software, algorithm	UCSF ChimeraX	UCSF Resource for Biocomputing, Visualization, and Informatics	RRID:SCR_015872	3D structure visualization and figure generation
Software, algorithm	Origin	OriginLab Corporation	RRID:SCR_014212	Data analysis and graphing
Software, algorithm	GraphPad Prism 10	GraphPad Software, Inc	RRID:SCR_002798	Data analysis and graphing
Other	ESF 921	Expression Systems	Cat number: 96-001-01	Insect Cell Culture Media
Other	DMEM high glucose with GlutaMAX	Thermo Fisher Scientific	Cat number: 10566016	Supplemented with 2 mM sodium pyruvate, 100 IU/ml penicillin, 250 µg/ml streptomycin, 10% Fetal Bovine Serum, 1% GlutaMAX
Other	Casein from bovine milk	Sigma-Aldrich	Cat number: C7078	
Other	Fibrinogen from human plasma	Sigma-Aldrich	Cat number: F3879	
Other	HiTrap IMAC FF 5 ml	Cytiva	Cat number: 17092104	Connected to an AKTA Pure system
Other	HiTrap Q XL 5 ml	Cytiva	Cat number: 17515901	Connected to an AKTA Pure system
Other	Superdex 200 increase 10/300 GL	Cytiva	Cat number: 28990944	Connected to an AKTA Pure system
Other	HiLoad 16/600 Superdex 75 pg	Cytiva	Cat number: 28989333	Connected to an AKTA Pure system
Other	Amicon Ultra Centrifugal Filter, 30 kDa MWCO	Millipore	Cat number: UFC9030	
Other	Amicon Ultra Centrifugal Filter, 10 kDa MWCO	Millipore	Cat number: UFC9010	

All other chemical reagents were purchased from Sigma-Aldrich/ Merck.

### SVMP expression construct design

PI, PII, and PIII SVMP constructs were designed by fusing an Avi- and His-tag (GGSGLNDIFEAQKIEWHEHHHHHHHH*) to the C terminus of the native SVMP sequences listed on UniProt (RRID:SCR_004426; PI – Uniprot ID: Q2UXQ3, PII – Uniprot ID: A0A3G1E3U2, PIII – Uniprot ID: E9JG34). The sequences were codon optimised for *Spodoptera frugiperda*, gene synthesised (Genscript) and inserted into the plasmid pACEBac1 (Geneva Biotech), which acted as the donor in the MultiBac system (Sari et al., 2016). Structure predictions of SVMP zymogens were performed using AlphaFold 3 (Abramson et al., 2024) in the presence of a single Zn^2+^. Per residue predicted Local Distance Difference Test (pLDDT) plots, indicating local confidence, were generated from B-factor values stored in the .cif files. The highest-ranked model among the five predictions was selected for visualisation and figure generation using UCSF ChimeraX (RRID:SCR_015872, https://www.cgl.ucsf.edu/chimerax/).

Mature SVMP constructs did not include the prodomain, but did contain an N-terminally fused melittin signal sequence (MKFLVNVALVFMVVYISYIYA). Propeptide-SVMP fusions contained an additional N-terminally fused propeptide triplicate and TEV site (GGSPKMCGVTPKMCGVTPKMCGVTENLYFQSN) following the melittin signal sequence. SVMP active site mutants were based on the mature SVMP constructs, but with the active site glutamic acid mutated to aspartic acid and glycine mutated to cysteine (Camacho et al., 2016). Zymogen constructs comprised the native signal sequence preceding the native prodomain (which is specific to the SVMP) and the SVMP sequence with a C-terminal Avi-His-tag fusion. PI was found to undergo C-terminal processing, the site of which was predicted by modelling using AlphaFold 3 (Abramson et al., 2024). PIΔC construct contained the Avi-His-tag fusion following this site.

### Recombinant SVMP expression

Each SVMP-containing pACEBac1 plasmid was expressed using the MultiBac baculovirus/insect cell expression system following established protocols (Sari-Ak et al., 2021). SVMPs were expressed in Hi5 insect cells at 19 °C, in ESF 921 Insect Cell Culture Media (Expression Systems). The cells were monitored throughout expression by measuring YFP fluorescence (encoded in baculoviral genome) and cell viability (by determining percentage of alive cells using Trypan Blue). A control expression of an unrelated, non-toxic protein (E3 ligase) was used as a control for YFP and cell viability measurements. Expression in the presence of Marimastat was tested using a final concentration of 0 μM, 3 μM, and 6 μM Marimastat in the cell culture media, which was added alongside the virus (at the start of expression). Cultures were harvested 5 days after infection by centrifugation (1000 × *g*, 10 min), or when the cell viability dropped below 80% if this occurred sooner. The secreted SVMP proteins were purified immediately after harvesting from the supernatant.

### Western blotting

Proteins resolved by SDS–PAGE were transferred to PVDF membranes (Trans-Blot Turbo mini 0.2 µm PVDF Transfer Pack, Bio-Rad) using a Trans-Blot Turbo system for 7 min at 2.5 A and 25 V. Membranes were blocked in 2.5% (w/v) skimmed milk in PBST either overnight at 4 °C or for 1 hr at room temperature, then washed three times for 5 min in PBST. For detection of His-tagged proteins, membranes were incubated for 1 hr at room temperature with HRP-conjugated anti-Penta-His antibody (diluted 1:2000 in PBS; QIAGEN), followed by PBST washes. For PDI detection, membranes were incubated overnight at 4 °C with rabbit anti-PDI antibody (Cell Signalling Technologies) diluted in PBS, washed in PBST, and then incubated for 2 hr at room temperature with IRDye 680RD Goat Anti-Rabbit IgG (diluted 1:10,000 in PBS) (LICORbio), followed by further PBST washes. Membranes were rinsed in PBS prior to imaging. His-tagged proteins were visualised using SuperSignal West Pico PLUS Chemiluminescent Substrate (Thermo Fisher Scientific) and the Synergene G: Box gel doc system, while PDI blots were imaged using a fluorescence-based detection system in the Odyssey Fc Imager (LICORbio). Transfer efficiency was assessed by Ponceau S staining following imaging.

### Co-expression of SVMPs with protein disulfide isomerase

The PII and PIII SVMPs were additionally co-expressed with human or snake PDI which was incorporated into the plasmid using Cre-lox (Haffke et al., 2013). The multigene expression construct was made using PDI-containing pIDC plasmids (Geneva Biotech). pIDC_hPDI and pIDC_sPDI (PDI-insert synthesised by Genscript) as the donor plasmids were fused with SVMP-containing pACEBac1 plasmids as the acceptor plasmid (Haffke et al., 2013). SVMP expression was carried out as above.

### Recombinant SVMP purification

Clarified media (containing SVMP) was continuously passed through HiTrap IMAC FF 5 ml column (Cytiva) at 2 ml/min, for 18 hr at 4 °C using a peristaltic pump. Subsequently, the column was washed with 10 CV high-salt buffer (50 mM Tris-Cl, 1 M NaCl, pH 8.0) and 10 CV SVMP buffer (50 mM Tris-Cl, 300 mM NaCl, pH 8.0). After the washing steps, the IMAC column was attached to an AKTA Pure system. The SVMP was eluted from the IMAC column using a gradient of imidazole from 0 mM to 500 mM imidazole in SVMP buffer. Fractions containing SVMP were pooled and dialysed into SVMP low-salt buffer (50 mM Tris-Cl, 50 mM NaCl, pH 8.0). SVMP was further purified by ion exchange chromatography by passing the dialysed protein through a 5 ml HiTrap Q XL column. Subsequently, the SVMP was eluted from the column by a gradient of 50 mM to 1 M NaCl in SVMP buffer. Fractions containing SVMP were pooled and concentrated to 500 μl (maximum 10 mg/ml). Finally, SVMP was purified by SEC using a Superdex 200 increase 10/300 GL column (Cytiva) in SVMP buffer with a flow rate of 0.375 ml/min. Cytiva Gel Filtration Calibration Kits were used to calibrate the column, with elution peaks plotted to generate a standard curve with which to calculate the MWs of SVMP proteins. Eluted SVMP was concentrated to 0.5–2 mg/ml using an Amicon concentrator with appropriate MW cut off. Glycerol was added to 5% before the protein was flash frozen in liquid nitrogen and stored at –80 °C.

### SVMP deglycosylation assay and PNGase F treatment

Glycosylation of SVMP zymogens was determined by incubation with peptide-*N*-glycosidase F (PNGase F, Promega) to remove N-linked oligosaccharide groups, followed by SDS-PAGE. Briefly, the reaction was carried out with 1–2 μg SVMP diluted in 12 μl water, followed by addition of 1 μl 5% SDS and 1 μl 1 M DTT. Next, the sample was heated at 95 °C for 5 min to denature the SVMP zymogens. Once cooled at room temperature, 2 μl SVMP buffer, 2 μl 10% NP-40 and 2 μl PNGase F were added. The reaction mix was incubated at 37 °C for 3 hr. Subsequently, SDS-PAGE sample buffer (reducing) was added, and the samples were analysed by SDS-PAGE. SVMP zymogen deglycosylation was identified by a shift towards lower MW of the SVMP band compared to the band where PNGase F was omitted.

### Purification of PIII SVMP fraction from *E. ocellatus* venom

As a procoagulant control for blood clot time assays, an *E. ocellatus* SVMP PIII fraction was prepared using SEC. Whole venom (25 mg sourced from the herpetarium facility at the Liverpool School of Tropical Medicine) was dissolved in 1.5 ml of PBS (25 mM sodium phosphate, 150 mM NaCl, pH 7.2), centrifuged and the supernatant loaded onto a 120 ml column of Superdex75 (Cytiva). Proteins were separated in PBS using a flow-rate of 1.0 ml/min and the separation was monitored at 280 nm. Two ml fractions were collected and SDS-PAGE was used to determine the fractions containing SVMP PIIIs. These were pooled and shown to be free of serine protease activity using the casein assay (see above) with appropriate inhibitors.

### SVMP enzyme activation

To determine optimal activation conditions, SVMP was titrated with 0–750 μM ZnCl_2_, then incubated at 26 °C or 37 °C for 18 hr. Activation was monitored using SDS-PAGE and disappearance of the SVMP zymogen band in Coomassie-stained gels.

### Casein and fibrinogen degradation assays

Activated SVMP was titrated into a freshly prepared 20 μl reaction mix containing casein or fibrinogen at a final concentration of 0.525 mg/ml in SVMP buffer. Final SVMP concentrations ranged from 0 to 2.34 μM (based on SVMP zymogen concentration at the start of the experiment). The negative control contained 5 mM EDTA as an SVMP inhibitor and maximum concentrations of SVMP (PI - 2.34 μM, PII –1.07 and 1.34 μM, PIII - 685 nM). Incubation was carried out at 37 °C for 18 hr. SDS-PAGE sample buffer was added, and the samples were heated to 95 °C to stop the reaction, and the degradation pattern was analysed by reducing SDS-PAGE.

### Fluorogenic peptide substrate cleavage assay

The quenched fluorogenic peptide Mca-KPLGL-Dpa-AR-NH_2_ (ES010, R&D Systems Inc) was used to kinetically measure SVMP activity, whereby the activated SVMP cleaves the Gly-Leu bond. Cleavage leads to the release of the Dpa-containing quencher from the fluorescent Mca-containing fragment. ES010 substrate was diluted in reaction buffer (50 mM Tris-Cl, 300 mM NaCl, pH 7.5) and used in the assay at final concentrations of 0 μM to 180 μM, in 100 μl in a 96-well plate. Enzymes were activated with Zn^2+^ (see above) and added to a final concentration of 40 or 20 nM based on the SVMP zymogen concentration at the start of the experiment. Fluorescence was followed for 30 min or 1 hr at 25 °C using a BioTech Synergy Neo2 instrument, an excitation wavelength of 320 nm and emission wavelength of 405 nm. Measurements were taken in triplicate. Kinetic data was plotted using Origin (RRID:SCR_014212, https://www.originlab.com/index.aspx?go=PRODUCTS/Origin), and a non-linear curve was fitted using the Michaelis-Menten function. Origin provided the best-fit values for V_max_ and K_M_, and k_cat_ could be calculated by V_max_/enzyme concentration. The fluorescence intensity versus time curve and the reaction rate were plotted and calculated using GraphPad Prism 10.

### Insulin B degradation assay

Insulin B degradation was determined following the established protocol (Wilkinson et al., 2024), using a final concentration of 0.02 mg/ml insulin B and 0.01 mg/ml activated SVMP, in 50 mM Tris-Cl, 150 mM NaCl, 1 mM CaCl_2_, pH 7.5 (TBS) buffer. Incubation was carried out at 37 °C for 120 min, then trifluoracetic acid was added to 1% to stop the reaction. The whole sample (=1 μg insulin B) was analysed by RP-HPLC using a C18 reverse phase column.

### Plate-based aggregometry

Research involving derivatives of human blood samples was approved by the United Bristol Healthcare NHS Trust Research Ethics Committee (project E5736), and all participants provided written informed consent in accordance with the Declaration of Helsinki. Blood samples were collected from healthy adult volunteers into 3.2% sodium citrate Vacutainer tubes, and platelet-rich plasma (PRP) was prepared by centrifugation of citrated whole blood at 180 × *g* for 17 min at room temperature. 2-MeS-ADP was prepared in HEPES-Tyrode’s buffer (10 mM HEPES pH 7.3, 145  mM NaCl, 1 mM MgSO_4_, 3 mM KCl), and experiments were performed using an end-point assay in 96-well half-area plates. 5 µL of each SVMP (PI, PII, PIII and PII zymogen) were incubated with 45 μL PRP for 5 min at 37 °C prior to platelet aggregation induction by 2-MeS-ADP (2.5 μM). The plate was shaken at 1200 rpm, 37 °C for 5 min, and the absorbance was then read at 595  nm using a Labtech LT4500 plate reader. To determine % aggregation, the sample absorbance was normalised accounting for unstimulated PRP as 0% aggregation and the control (buffer without SVMP) as 100% of aggregation. All means were calculated based on three assays performed in duplicate. A two-way ANOVA was conducted, followed by simple effects analysis (Dunnett post-test) to compare column means within each row, using GraphPad Prism 10.

### Bovine plasma clotting assay

The assay was carried out following the protocol described by Still et al., 2017. Briefly, 20 μl activated SVMP or EchOce NGA (Nigerian *E. ocellatus* venom) at 20 μg/ml was added to 40 μl of 20 mM CaCl_2_, and then 40 μl of citrated bovine plasma (Biowest S0260-500) was added immediately before measuring absorbance at 595 nm on a CLARIOstar Plus microplate reader. The clot formation was monitored for 30 min at room temperature. To obtain the normal clotting curve, 20 µl of PBS was added. For the no clotting control, CaCl₂ was replaced with 20 µl of PBS. Absorbance versus time curves were plotted using GraphPad Prism 10.

### Thrombin/ SVMP clot time assay

Experiments were carried out following manufacturer’s kit instructions (HemosIL, Thrombin Time 0009758515), using an ACL TOP coagulation analyser using 3.0 U/ml thrombin, 500 ng PIII SVMP pool purified from *E. ocellatus* venom and 500 ng activated recombinant PIII SVMP. The assay measures the rate of increase in turbidity as the fibrin clot forms. The peak in the rate of change (second derivative) is considered to be the point of clot formation.

### MTT cell viability assay

MTT assay was based on the methods of Hall et al., 2023. Immortalised human epidermal keratinocytes, HaCaT (AddexBio, certified mycoplasma-free cells derived from a 62-year-old Caucasian male donor’s skin epidermis, RRID:CVCL_0038) were seeded (5000 cells/well, clear-sided 96-well plates) in standard medium (DMEM high glucose with GlutaMAXTM supplemented with 2 mM sodium pyruvate, 100 IU/ml penicillin, 250  µg/ml streptomycin, 10% Fetal Bovine Serum, 1% GlutaMAXTM [Thermo Fisher Scientific, #35050061]), then left to adhere overnight at 37 °C, 5% CO_2_. The cell line was freshly procured and checked for contamination before the start of the experiment. The next day, the SVMPs treatments (25, 50, and 100 µg/ml) were prepared in standard medium, and cells were treated with each prepared solution (100 µl/well, duplicate wells) for 24  hr. For this assay, the MTT Cell Viability Assay Kit (Biotium) was used: 10 μl of MTT solution was added to the 100 μl of medium in each well and mixed by tapping gently on the side of the tray. The cells were incubated at 37 °C for 4 hr. 200 μl DMSO was added directly into the medium in each well and pipetted up and down several times to dissolve the formazan salt. The absorbance was measured on BioTek Synergy Neo2 spectrophotometer at 570 nm. The background absorbance was also measured at 630 nm (subtracted background absorbance from signal absorbance to obtain normalised absorbance values). The % of cell viability for each treatment well was calculated as follows with buffer-treated cells as a control (100% viability):

$$\displaystyle \frac{Normalised \, abs \, treatment \, well- Average \, normalised \, abs \, blank \, wells}{Average\, normalised\, abs \, cell \, control \, wells- Average \,normalised \, abs \, blank \, wells}$$

### Materials availability

Materials from this study are available from the corresponding authors upon reasonable request.

## Data Availability

All data generated or analysed during this study are included in the manuscript and supporting files.

## Additional files

MDAR checklist

## Appendix 1

### Optimisation of SVMP expression constructs

Initially, recombinant production of mature SVMPs, without the N-terminally fused prodomain, was attempted (Figure 2—figure supplement 1a). Protein expression yields, however, were extremely low, primarily due to SVMP cytotoxicity causing cell death immediately upon baculoviral infection. This was evidenced by very low cell viability, with more than 60% of cells dying within 72 hr of expression, and 100% cell death by 96 hr into the expression for the PI and PIII SVMPs (Figure 2—figure supplement 1a). Yellow fluorescent protein (YFP) is used in the MultiBac expression system as an indicator of baculovirus performance and a proxy for heterologous protein production (Bieniossek et al., 2012). Typically, YFP fluorescence is followed during expression and cells/proteins are harvested when YFP production reaches a plateau (Bieniossek et al., 2012). Throughout mature SVMP expression, YFP fluorescence remained very low, consistent with pervasive premature cell death caused by the SVMP toxin resulting in low YFP production and cell lysis leading to release of YFP into the media (Figure 2—figure supplement 1a). Additionally, the decrease in YFP readings observed (days 3–5 after proliferation arrest) during expression could also be due to proteolytic degradation of YFP by the mature, active SVMP.

Western blot analysis detecting the His-tag fused C-terminally to the SVMP showed that the mature PI and PIII had been successfully expressed within the cells. It also indicated that these SVMPs were soluble because they could be detected not only in the whole cell extract (SNP; combined supernatant/pellet) but also in the cleared lysate (SN; supernatant; Figure 2—figure supplement 1a). However, no SVMP was detected in the media sample in the western blot, indicating that little or no SVMP had been secreted. This is possibly due to the premature death and lysis of the cells caused by the toxin, impairing the secretion machinery before noticeable secretion had occurred. Taken together, expression of mature SVMPs detrimentally affects cell integrity, damaging the cells early after baculovirus infection and as soon as protein production initiates, underscoring the toxicity of the SVMPs.

Marimastat binds to the active site of the metalloproteinase mimicking the structure of a peptide substrate, inhibiting SVMP activity (Xie et al., 2020). During baculovirus infection, Marimastat was added to the medium at a final concentration of 3 µM and 6 µM, respectively (Figure 2—figure supplement 1b). At these concentrations however, Marimastat could not prevent cell death induced by PIII SVMP expression. On the contrary, the cell viability in the presence of 3 µM and 6 µM Marimastat was even further decreased. Additionally, less SVMP was detected in anti-His-tag western blots in whole cell extract (SNP) and cleared lysate (SN), compared to the expression of mature SVMP without Marimastat (Figure 2—figure supplement 1a). Thus, Marimastat itself appears to be toxic to insect cells at the doses tested and has no positive effect on SVMP expression and recovery.

*SVMP active site mutants* were designed based on a previous report of an inactive PII SVMP purified from *Bothriechis lateralis* venom (Camacho et al., 2016). Two active site mutations were identified in the protein rendering it inactive (canonical sequence: HEXXHXXGXXH, inactive sequence: H**D**LGHNL**C**IDH). We inserted these mutations into our PI and PIII SVMPs. We found improved cell viability and detected elevated YFP signal for the PI SVMP active site mutant (Figure 2—figure supplement 1c). However, the PIII SVMP active site mutant still showed significant toxicity, reducing YFP levels as well as cell viability. Nonetheless, we successfully purified both PI and PIII SVMP active site mutants from the media using immobilised metal affinity purification (IMAC) via the octa-histidine tags present in our proteins. Based on our observations with the active site mutants, we concluded that inhibition of metalloproteinase activity during SVMP protein production has a positive effect, indicating that expressing inactivated SVMPs could be a viable, although inefficient, approach.

Next, we designed propeptide-SVMP fusion proteins using the propeptide responsible for the cysteine switch, PKMCGVT. We fused three propeptide repeats to the N-terminus of the SVMP followed by a TEV protease cleavage site. We hypothesised that this would increase the avidity of the propeptide to the MP active site and efficiently inactivate the enzyme. Cell viability for the propeptide-SVMP fusion expression trials was higher at days 3–5 when compared to the mature SVMPs (Figure 2—figure supplement 1a and d). However, towards the end of expression less than 25% of cells were left alive (day 5), likely limiting protein secretion. Although YFP fluorescence levels for PI and PIII had slightly increased (Figure 2—figure supplement 1d) compared to expression of the mature SVMP (Figure 2—figure supplement 1a), YFP expression was still significantly lower as compared to a positive control which expressed an unrelated, non-toxic protein. Western blot analysis confirmed that PI and PIII SVMPs were expressed and soluble (Figure 2—figure supplement 1d). However, propeptide-SVMP fusion protein yields in the media remained too low to progress to purification.

### Co-expression of protein disulfide isomerases with SVMP zymogens

We generated expression cassettes for human PDI or snake PDI alongside our SVMPs and used Cre-LoxP recombination to integrate these cassettes into our SVMP expression constructs (Haffke et al., 2013). Successful co-expression of human PDI or snake PDI, alongside our SVMPs, was confirmed by western blot and mass spectrometry analysis (Figure 3—figure supplement 6a). Contrary to our expectation, however, co-expression of PII and PIII SVMP zymogens with these PDIs did not improve the yield of SVMP zymogens: The SEC chromatograms for PII SVMP zymogens after IMAC and IEX purification expressed in the presence and absence of co-expressed PDI aligned perfectly, with identical peak areas (Figure 3—figure supplement 6b). Similarly, the SEC chromatograms for purified PIII SVMP zymogens are virtually identical in the presence or absence of PDIs (Figure 3—figure supplement 6c). In conclusion, PDI overexpression in insect cells does not enhance the expression and/or folding of our SVMP zymogens and therefore did not result in a noticeable improvement in yield.

It remained possible, however, that the activity of these SVMPs could have been enhanced by the co-expression of the PDIs resulting in improved protein folding, for example through optimised disulfide bond formation. When tested against fibrinogen as a substrate, however, the activities of the different SVMPs remained unchanged by PDI co-expression (Figure 3—figure supplement 6d and e).
