# Supplementary figures and images for: Purified zymogens reveal mechanisms of snake venom metalloproteinase auto-activation
Source: eLife. 2026 Jun 10;15:RP109112. doi: 10.7554/eLife.109112 (PMC13252954; doi:10.7554/eLife.109112)

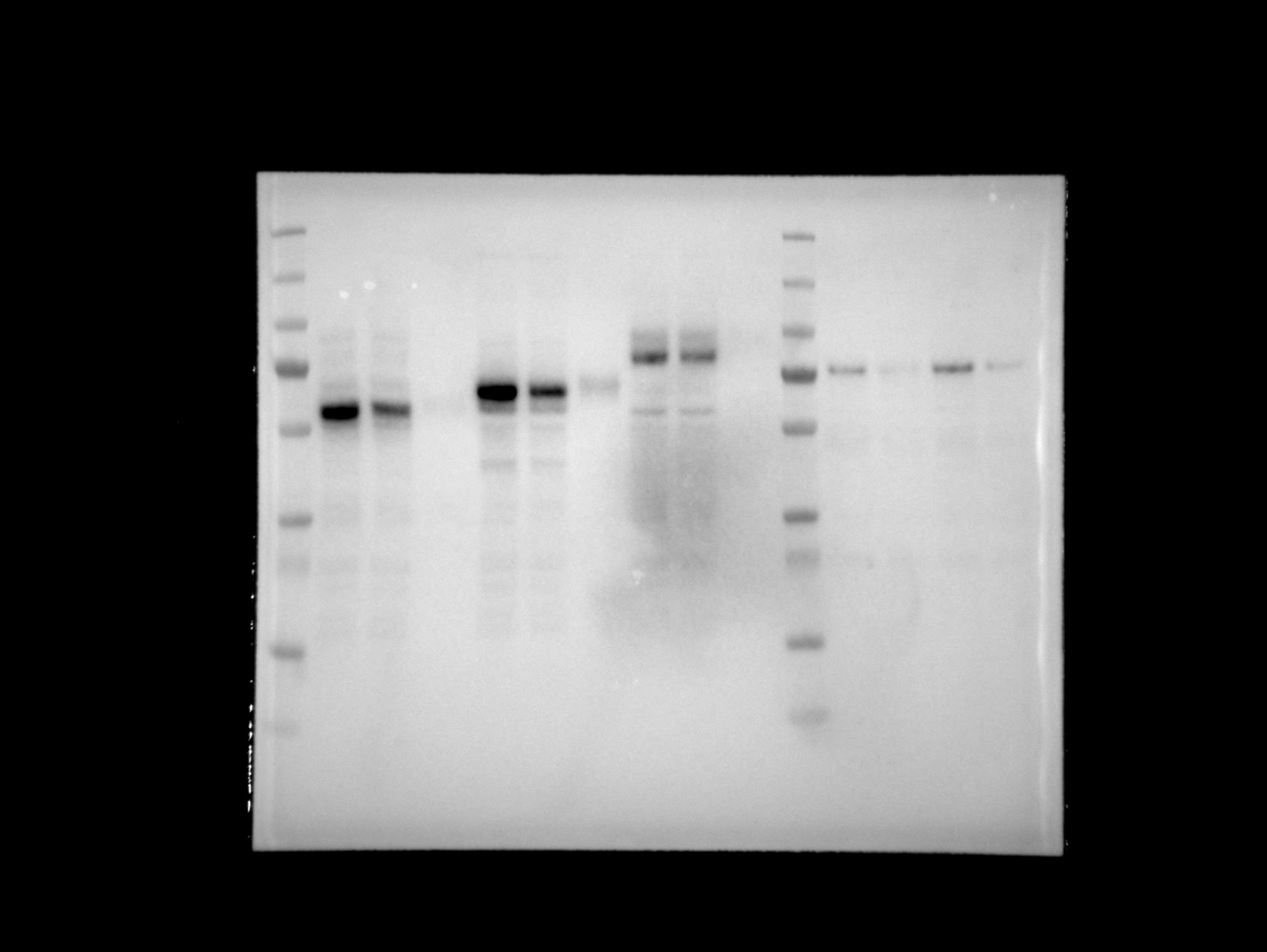

Supplement: Figure 2—source data 1. [file elife-109112-fig2-data1.zip › Figure 2 - source data 1/Figure 2d - source data 1.jpg]

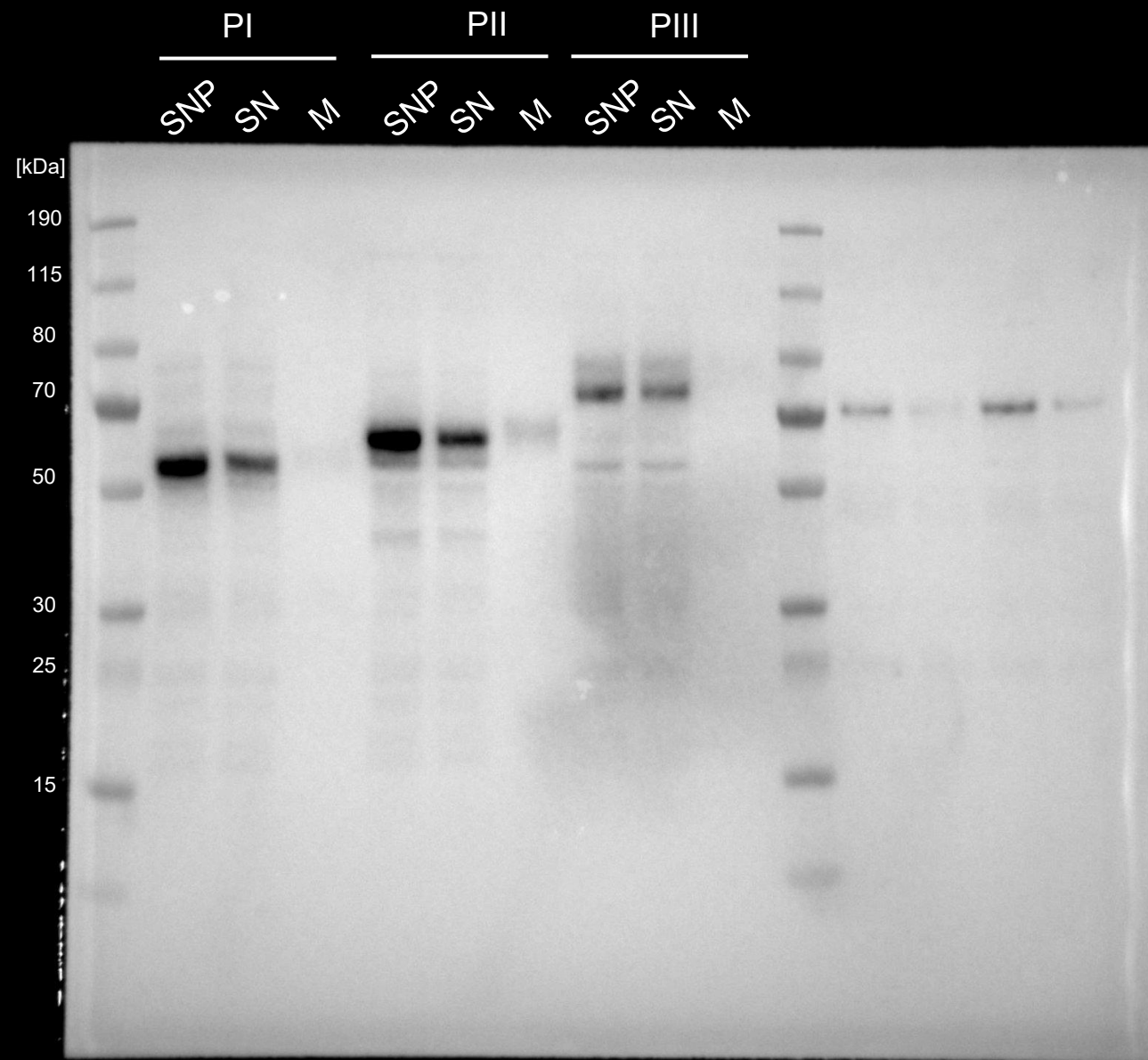

Supplement: Figure 2—source data 2. [file elife-109112-fig2-data2.zip › Figure 2d - source data 2.pdf]

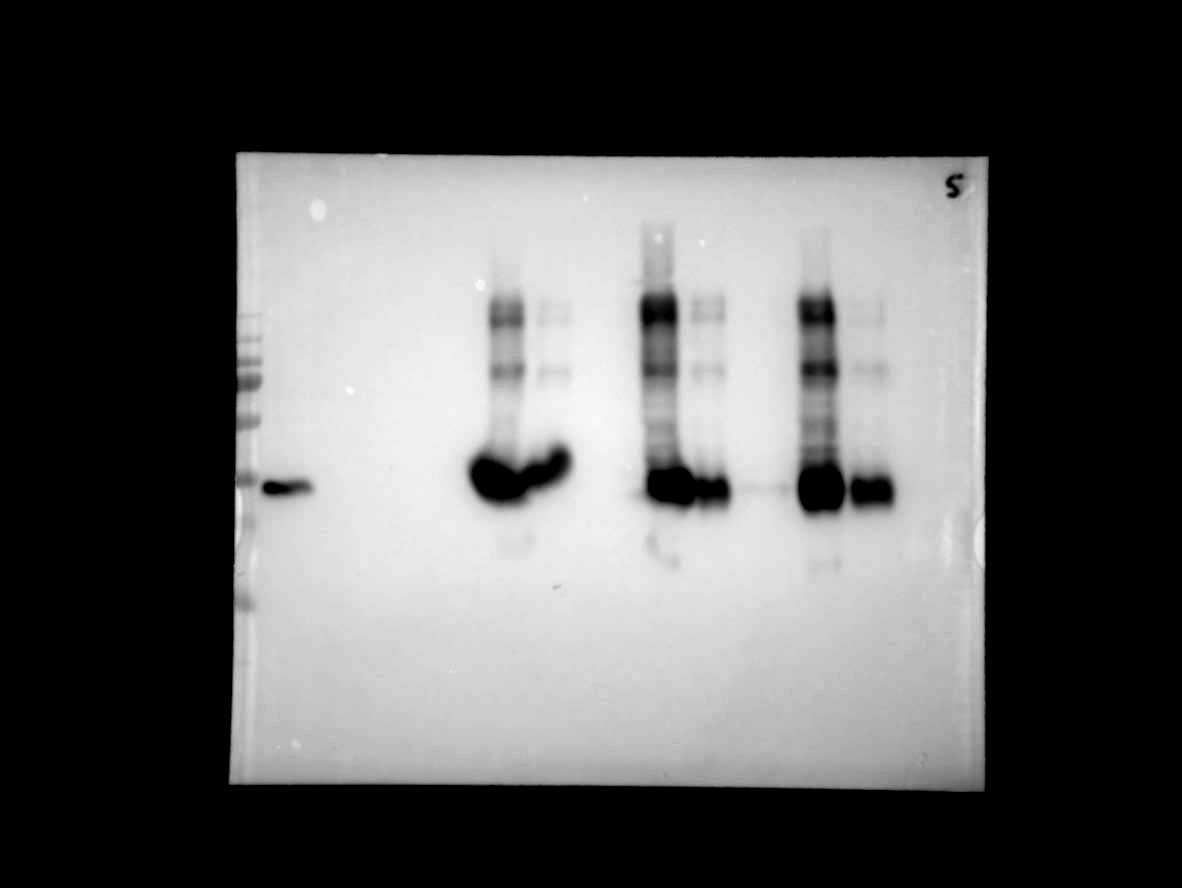

Supplement: Figure 2—figure supplement 1—source data 1. [file elife-109112-fig2-figsupp1-data1.zip › Figure 2 supplement 1 - source data 1/Figure 2 supplement 1 - source data 1/Figure 2 supplement 1a - source data 1.1.jpg]

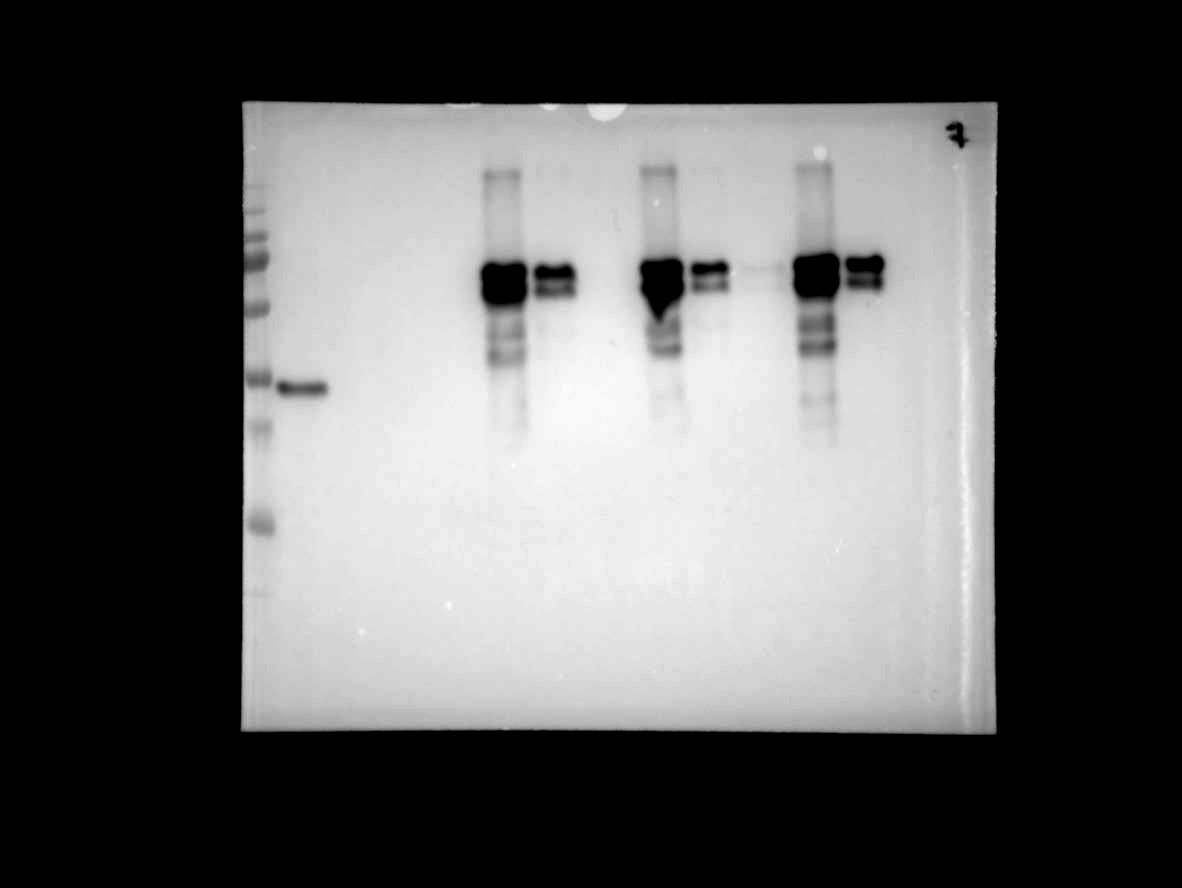

Supplement: Figure 2—figure supplement 1—source data 1. [file elife-109112-fig2-figsupp1-data1.zip › Figure 2 supplement 1 - source data 1/Figure 2 supplement 1 - source data 1/Figure 2 supplement 1a - source data 1.2.jpg]

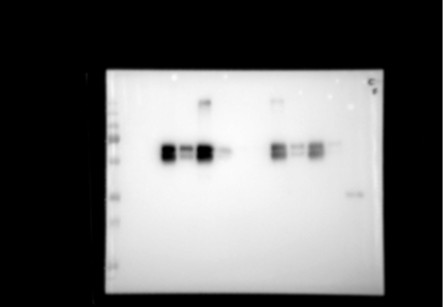

Supplement: Figure 2—figure supplement 1—source data 1. [file elife-109112-fig2-figsupp1-data1.zip › Figure 2 supplement 1 - source data 1/Figure 2 supplement 1 - source data 1/Figure 2 supplement 1b - source data 1.jpg]

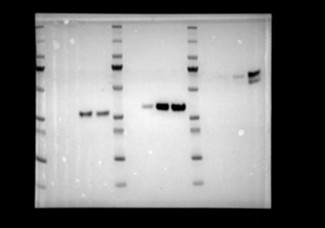

Supplement: Figure 2—figure supplement 1—source data 1. [file elife-109112-fig2-figsupp1-data1.zip › Figure 2 supplement 1 - source data 1/Figure 2 supplement 1 - source data 1/Figure 2 supplement 1c - source data 1.jpg]

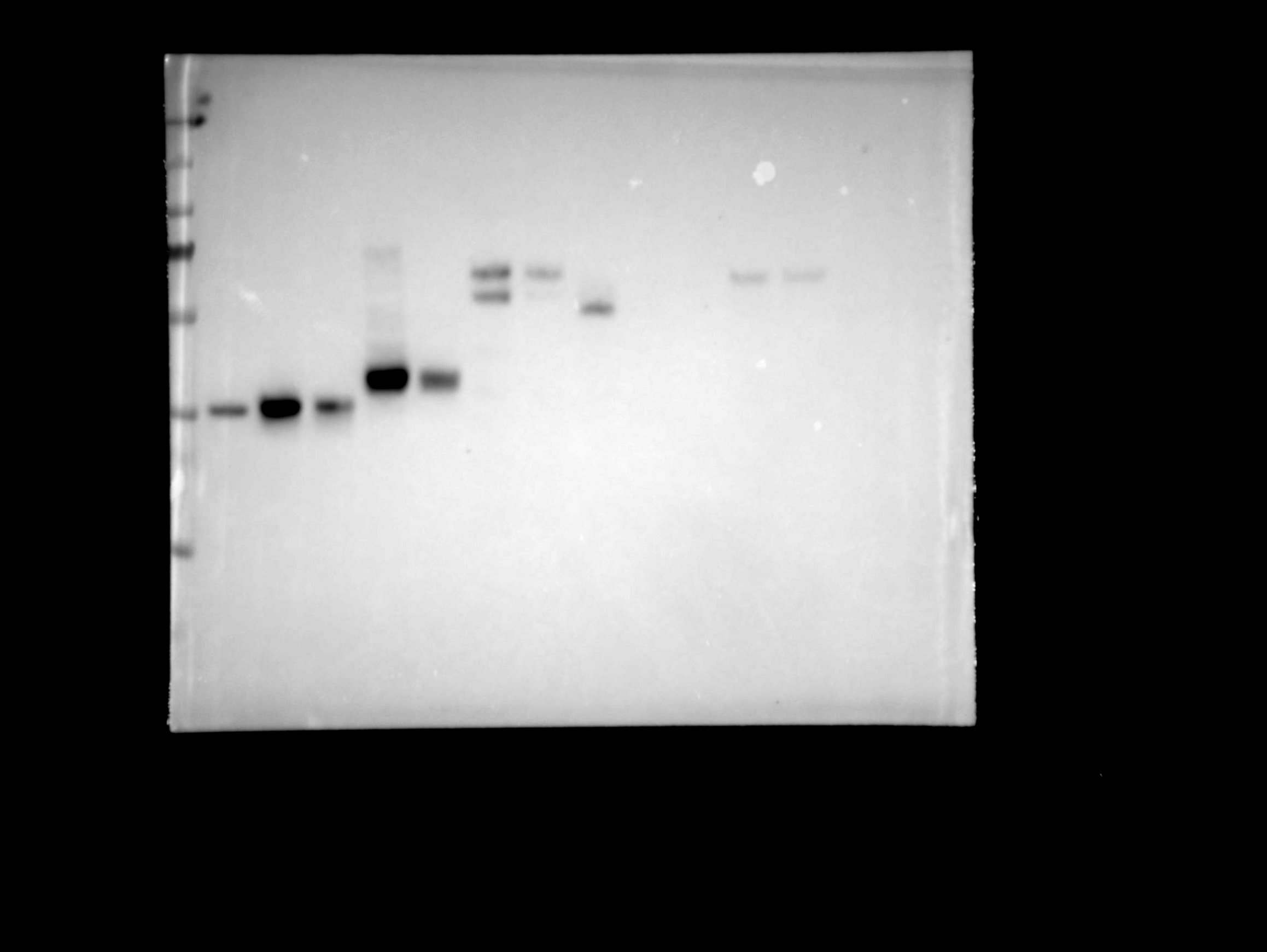

Supplement: Figure 2—figure supplement 1—source data 1. [file elife-109112-fig2-figsupp1-data1.zip › Figure 2 supplement 1 - source data 1/Figure 2 supplement 1 - source data 1/Figure 2 supplement 1d - source data 1.jpg]

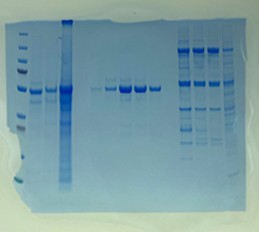

Supplement: Figure 3—source data 1. [file elife-109112-fig3-data1.zip › Figure 3 - source data 1/Figure 3a - source data 1.jpg]

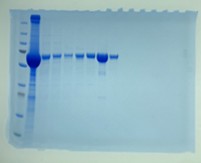

Supplement: Figure 3—source data 1. [file elife-109112-fig3-data1.zip › Figure 3 - source data 1/Figure 3b - source data 1.jpg]

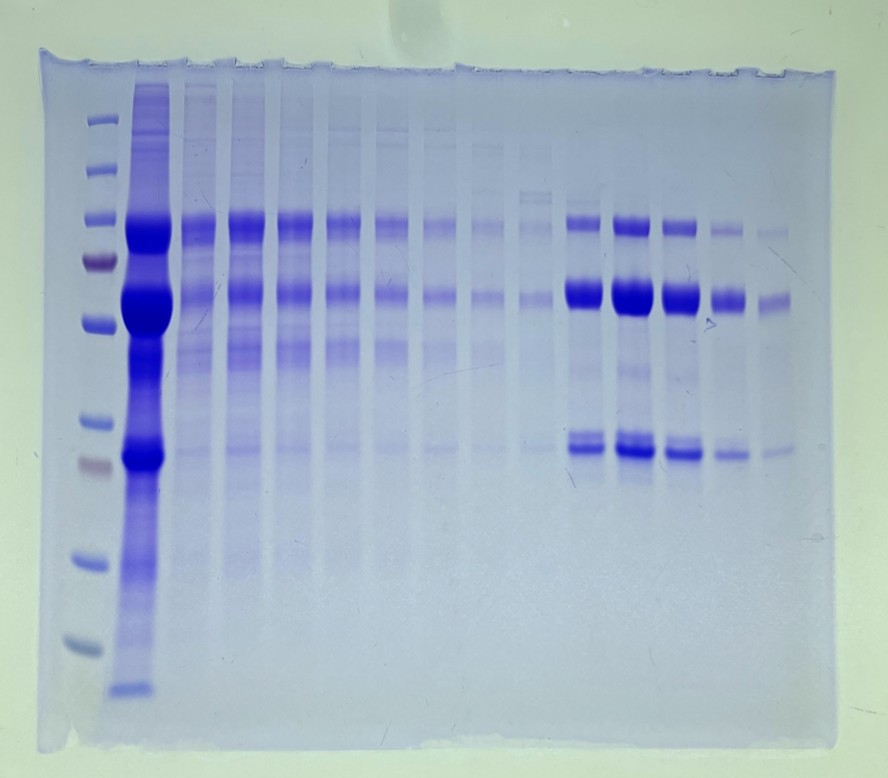

Supplement: Figure 3—source data 1. [file elife-109112-fig3-data1.zip › Figure 3 - source data 1/Figure 3c - source data 1.jpg]

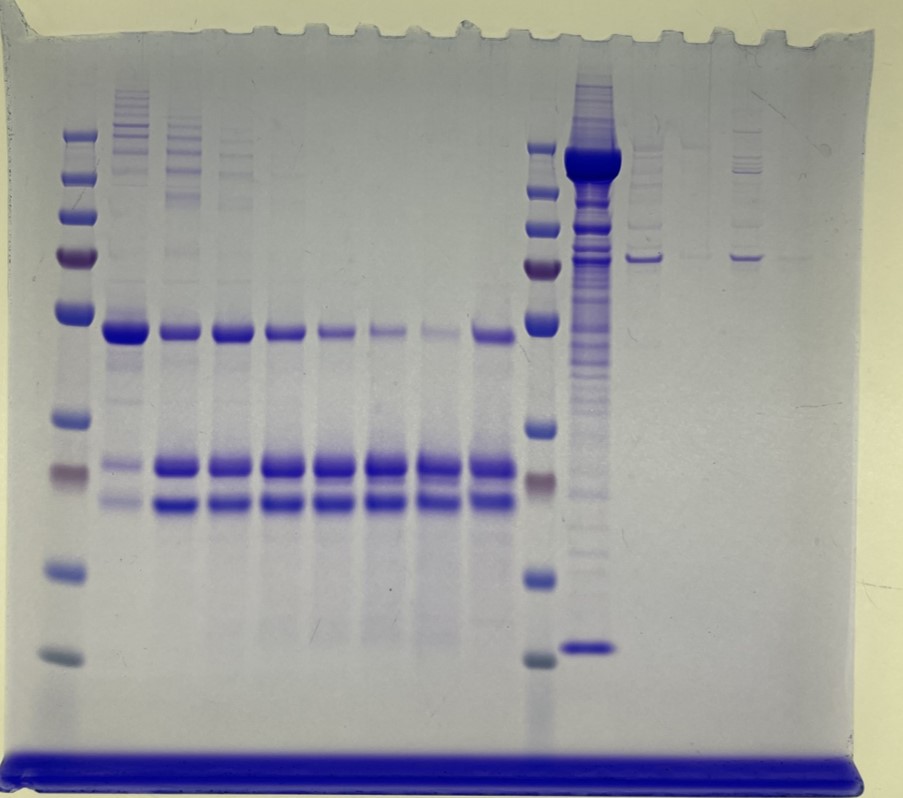

Supplement: Figure 3—source data 1. [file elife-109112-fig3-data1.zip › Figure 3 - source data 1/Figure 3d - source data 1.jpg]

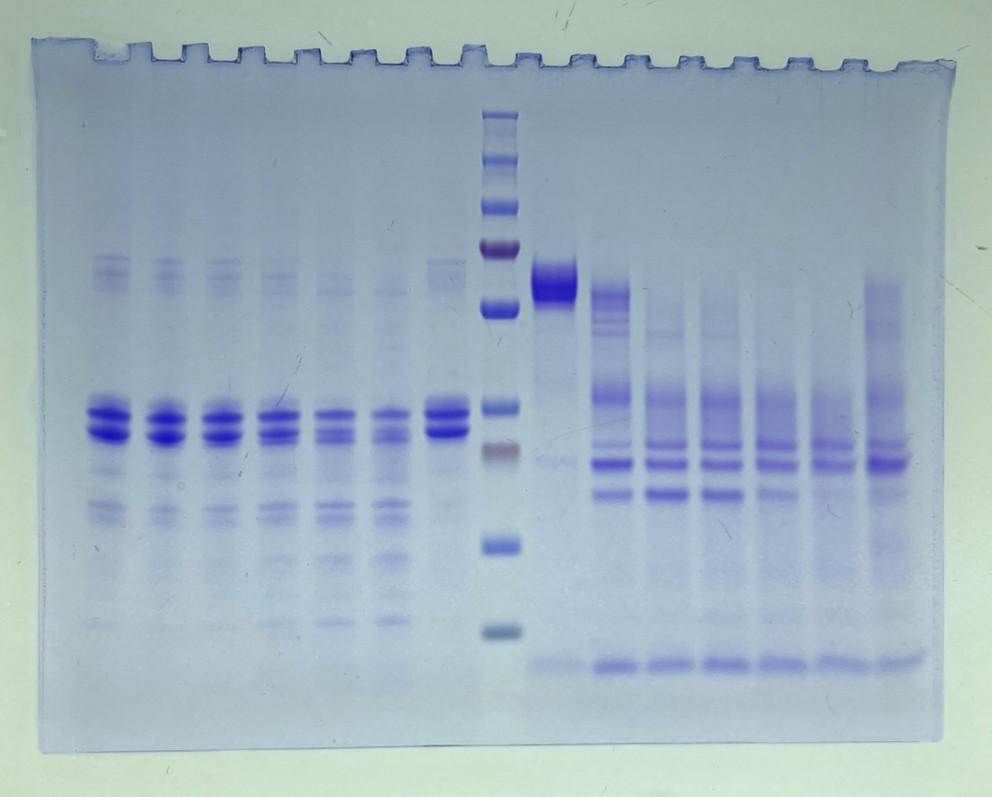

Supplement: Figure 3—source data 1. [file elife-109112-fig3-data1.zip › Figure 3 - source data 1/Figure 3e - source data 1.jpg]

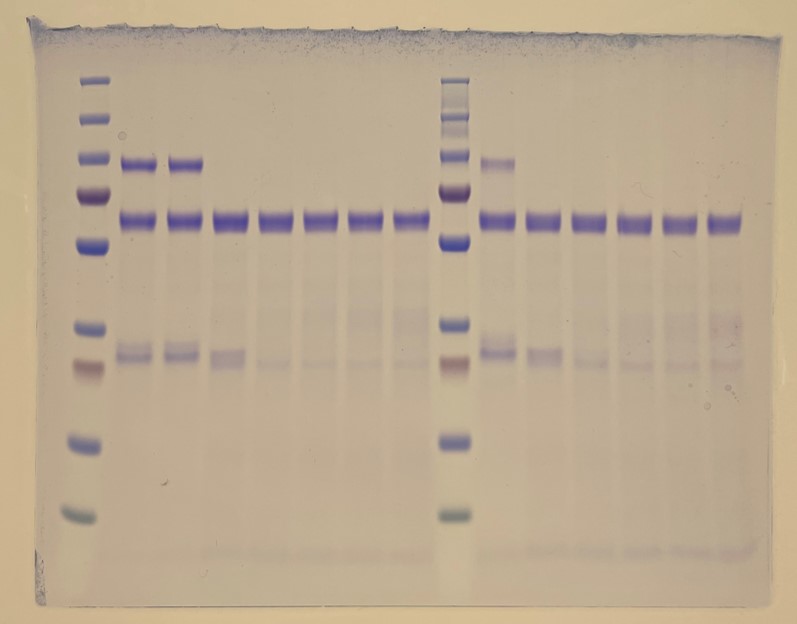

Supplement: Figure 3—source data 1. [file elife-109112-fig3-data1.zip › Figure 3 - source data 1/Figure 3f - source data 1.jpg]

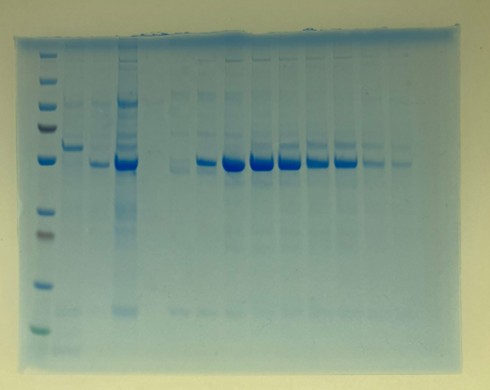

Supplement: Figure 3—figure supplement 1—source data 1. [file elife-109112-fig3-figsupp1-data1.zip › Figure 3 supplement 1 - source data 1/Figure 3 supplement 1 - source data 1/Figure 3 supplment 1a - source data 1.jpg]

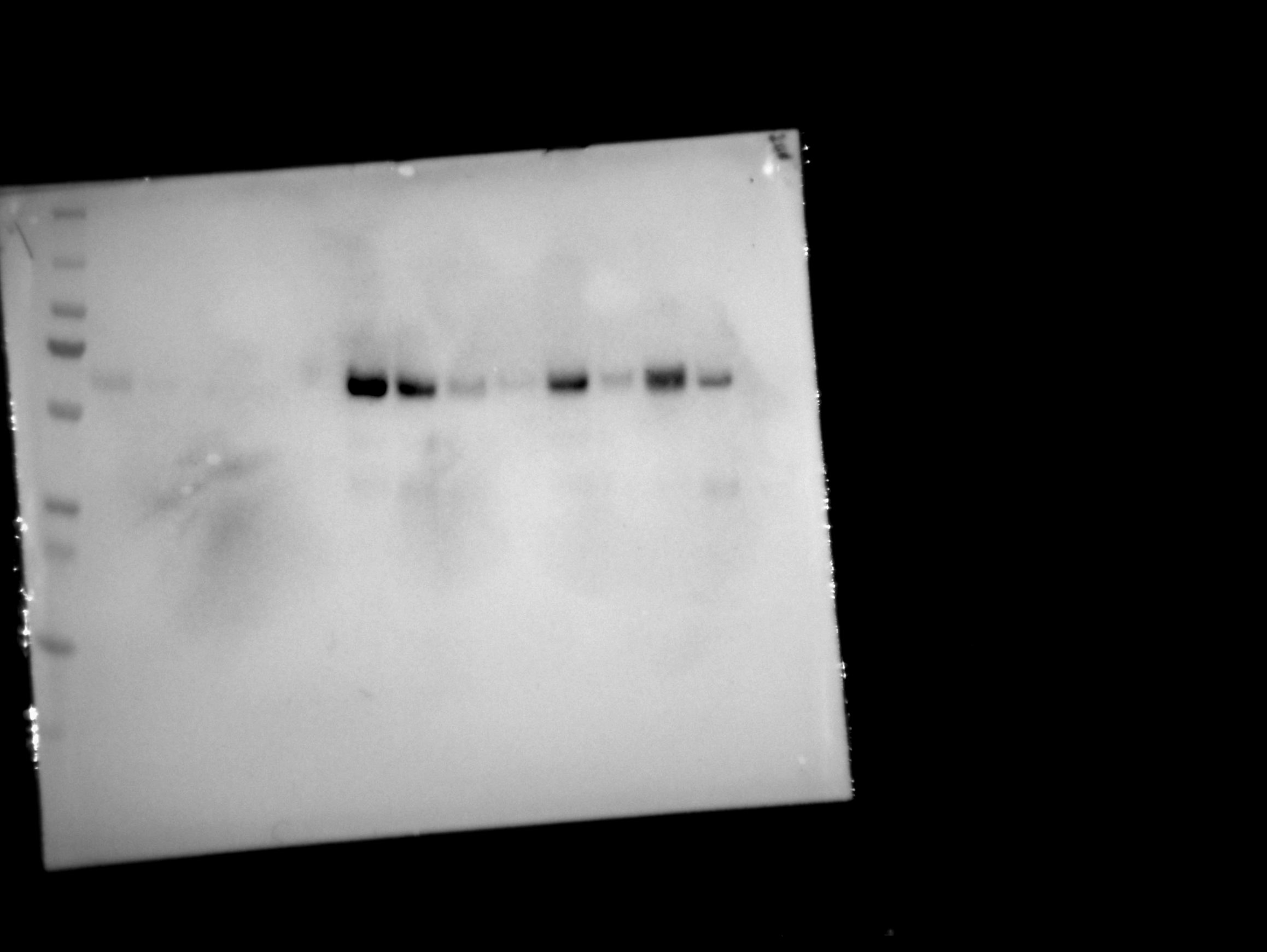

Supplement: Figure 3—figure supplement 1—source data 1. [file elife-109112-fig3-figsupp1-data1.zip › Figure 3 supplement 1 - source data 1/Figure 3 supplement 1 - source data 1/Figure 3 supplment 1b - source data 1.jpg]

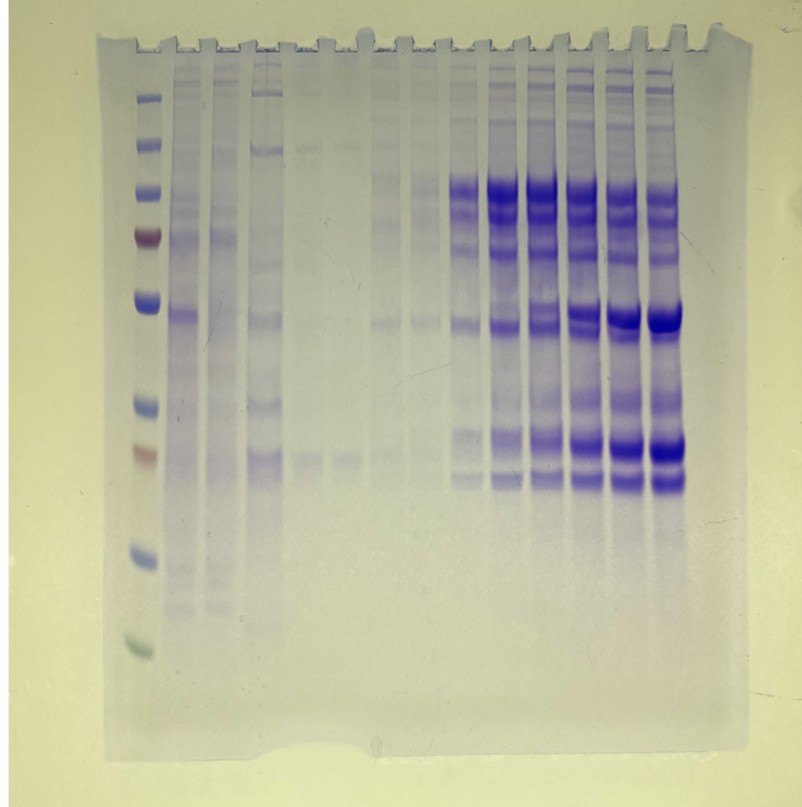

Supplement: Figure 3—figure supplement 2—source data 1. [file elife-109112-fig3-figsupp2-data1.zip › Figure 3 supplement 2 - source data 1/Figure 3 supplement 2 - source data 1/Figure 3 supplement 2a source data 1.1.jpg]

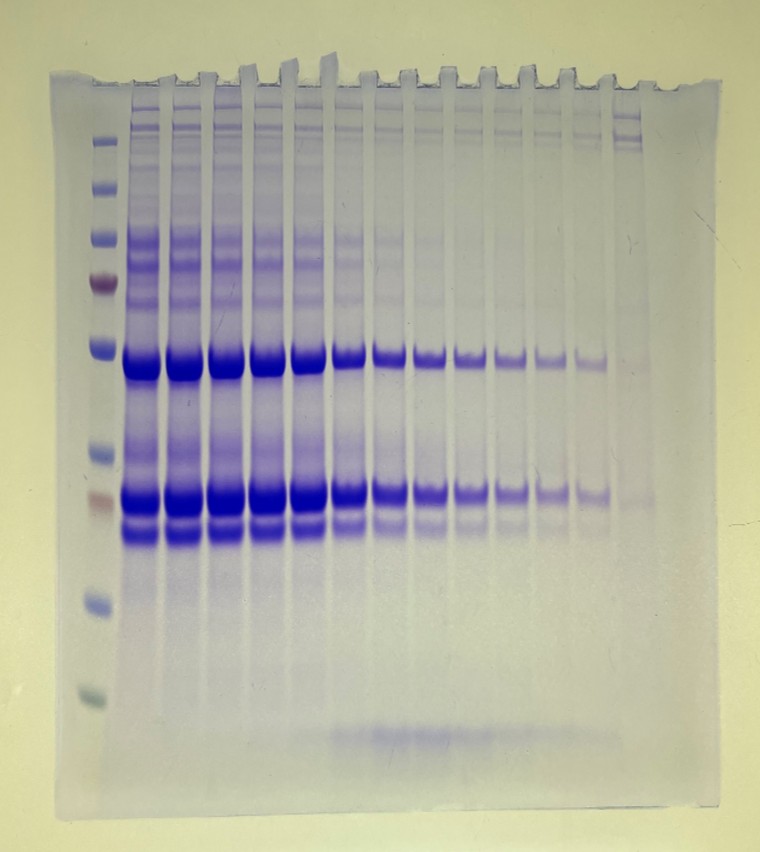

Supplement: Figure 3—figure supplement 2—source data 1. [file elife-109112-fig3-figsupp2-data1.zip › Figure 3 supplement 2 - source data 1/Figure 3 supplement 2 - source data 1/Figure 3 supplement 2a source data 1.2.jpg]

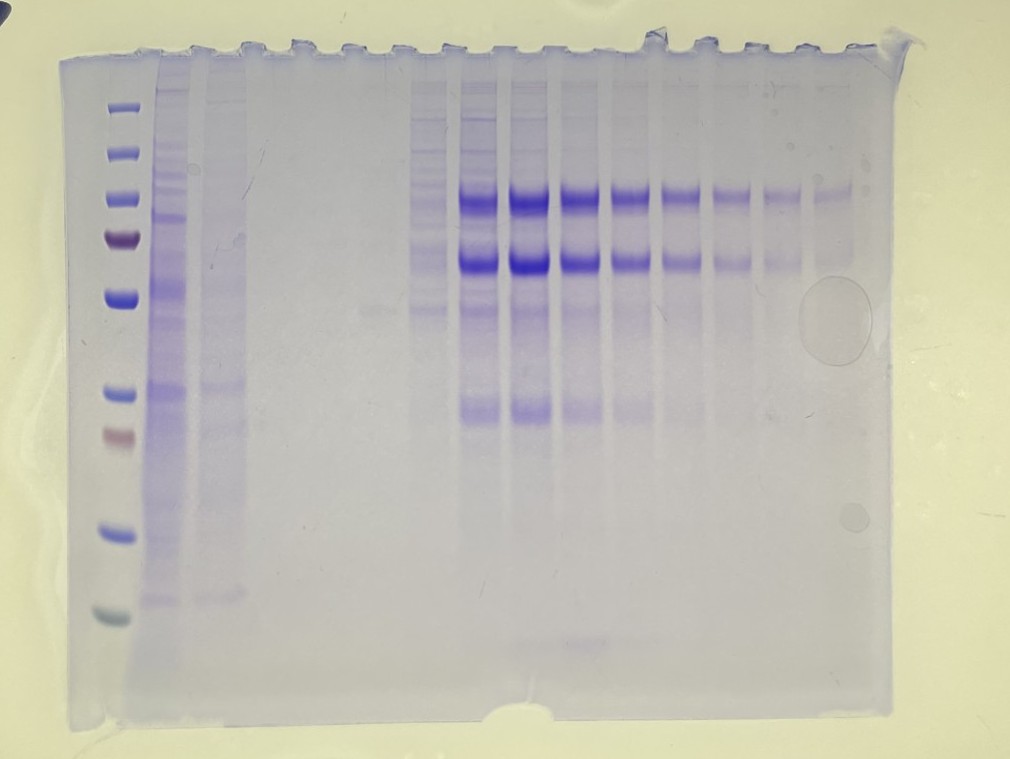

Supplement: Figure 3—figure supplement 2—source data 1. [file elife-109112-fig3-figsupp2-data1.zip › Figure 3 supplement 2 - source data 1/Figure 3 supplement 2 - source data 1/Figure 3 supplement 2b - source data 1.jpg]

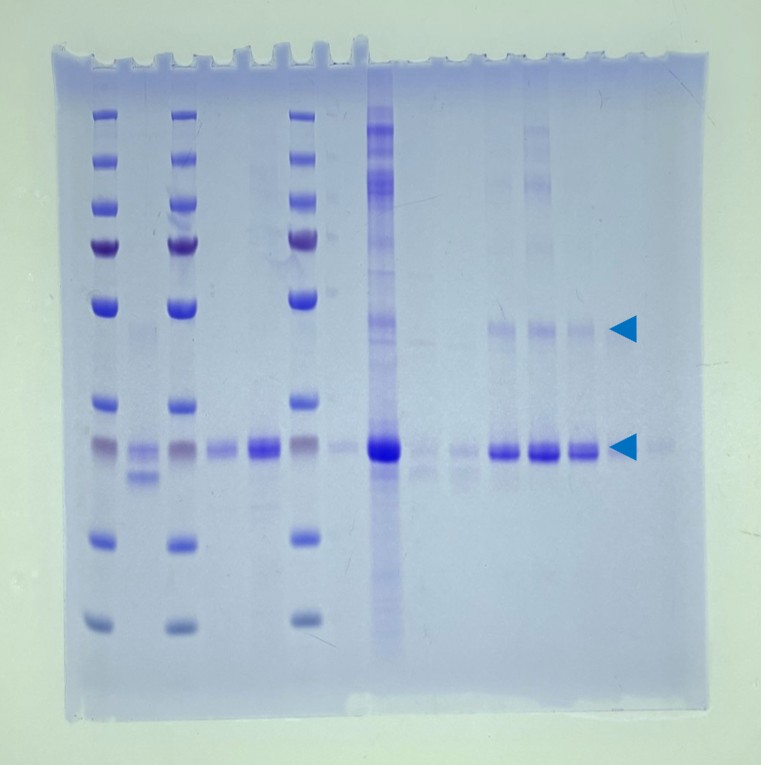

Supplement: Figure 3—figure supplement 3—source data 1. [file elife-109112-fig3-figsupp3-data1.zip › Figure 3 supplement 3 - source data 1/Figure 3 supplement 3 - source data 1/Figure 3 supplement 3a - source data.jpg]

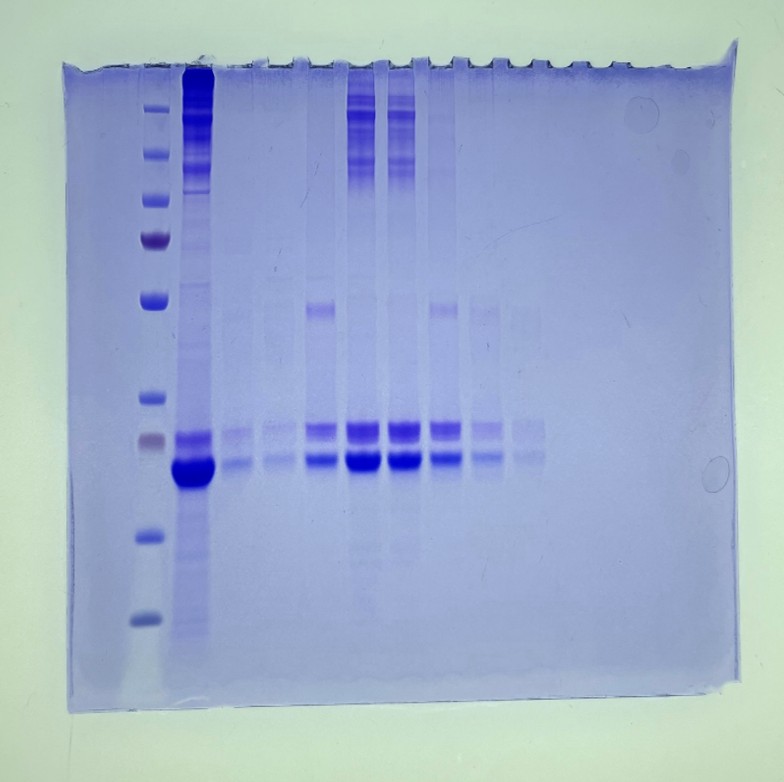

Supplement: Figure 3—figure supplement 3—source data 1. [file elife-109112-fig3-figsupp3-data1.zip › Figure 3 supplement 3 - source data 1/Figure 3 supplement 3 - source data 1/Figure 3 supplement 3b - source data.jpg]

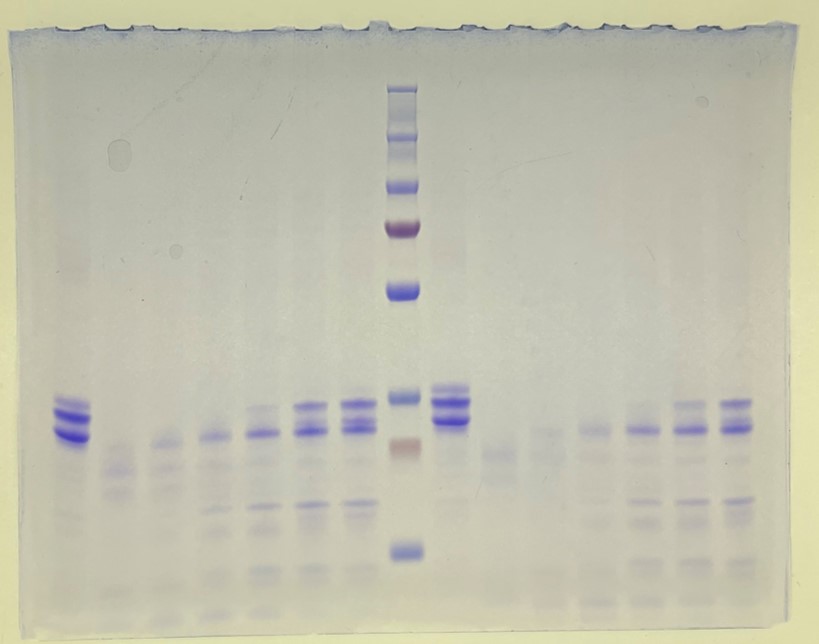

Supplement: Figure 3—figure supplement 3—source data 1. [file elife-109112-fig3-figsupp3-data1.zip › Figure 3 supplement 3 - source data 1/Figure 3 supplement 3 - source data 1/Figure 3 supplement 3c - source data 1.2.jpg]

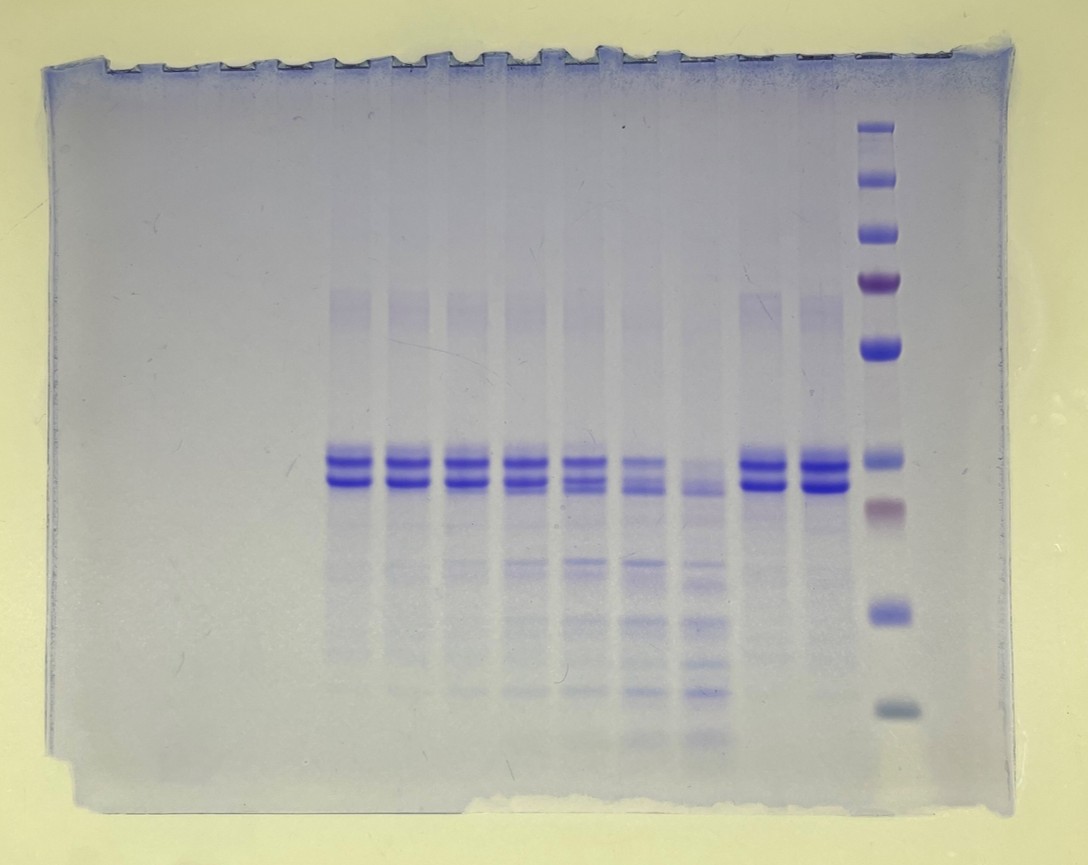

Supplement: Figure 3—figure supplement 3—source data 1. [file elife-109112-fig3-figsupp3-data1.zip › Figure 3 supplement 3 - source data 1/Figure 3 supplement 3 - source data 1/Figure 3 supplement 3c - source gel 1.1.jpg]

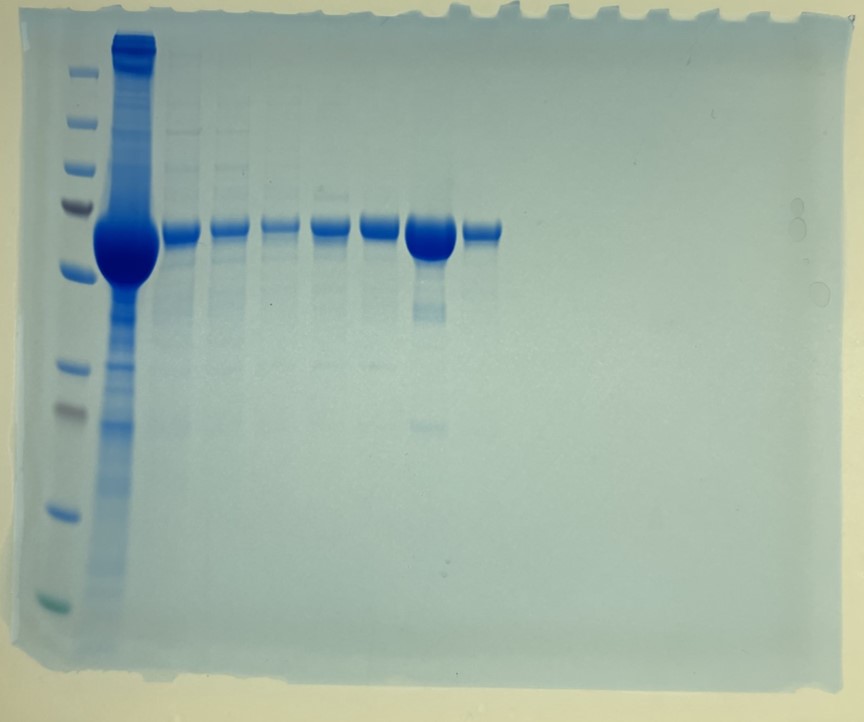

Supplement: Figure 3—figure supplement 4—source data 1. [file elife-109112-fig3-figsupp4-data1.zip › Figure 3 supplement 4 - source data 1/Figure 3 supplement 4 - source data 1/Figure 3 supplement 4b - source data 1.1.jpg]

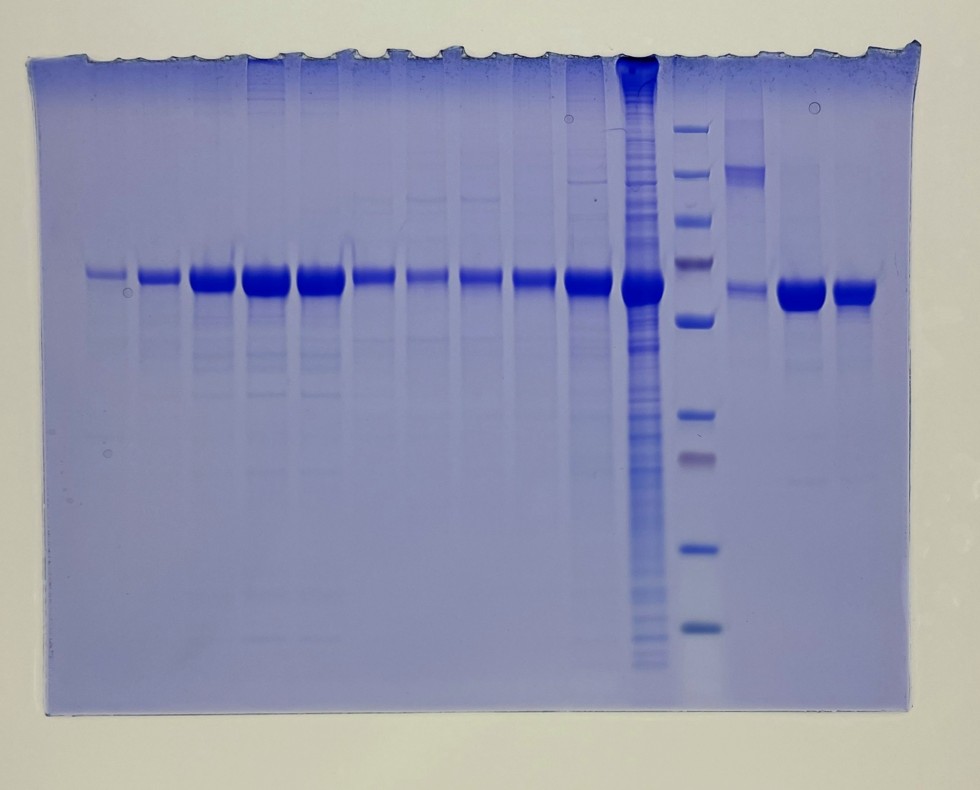

Supplement: Figure 3—figure supplement 4—source data 1. [file elife-109112-fig3-figsupp4-data1.zip › Figure 3 supplement 4 - source data 1/Figure 3 supplement 4 - source data 1/Figure 3 supplement 4b - source data 1.2.jpg]

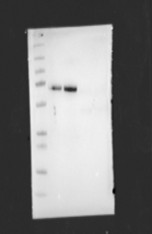

Supplement: Figure 3—figure supplement 4—source data 1. [file elife-109112-fig3-figsupp4-data1.zip › Figure 3 supplement 4 - source data 1/Figure 3 supplement 4 - source data 1/Figure 3 supplement 4c - source data 1.1.jpg]

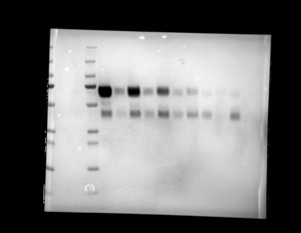

Supplement: Figure 3—figure supplement 4—source data 1. [file elife-109112-fig3-figsupp4-data1.zip › Figure 3 supplement 4 - source data 1/Figure 3 supplement 4 - source data 1/Figure 3 supplement 4c - source data 1.2.jpg]

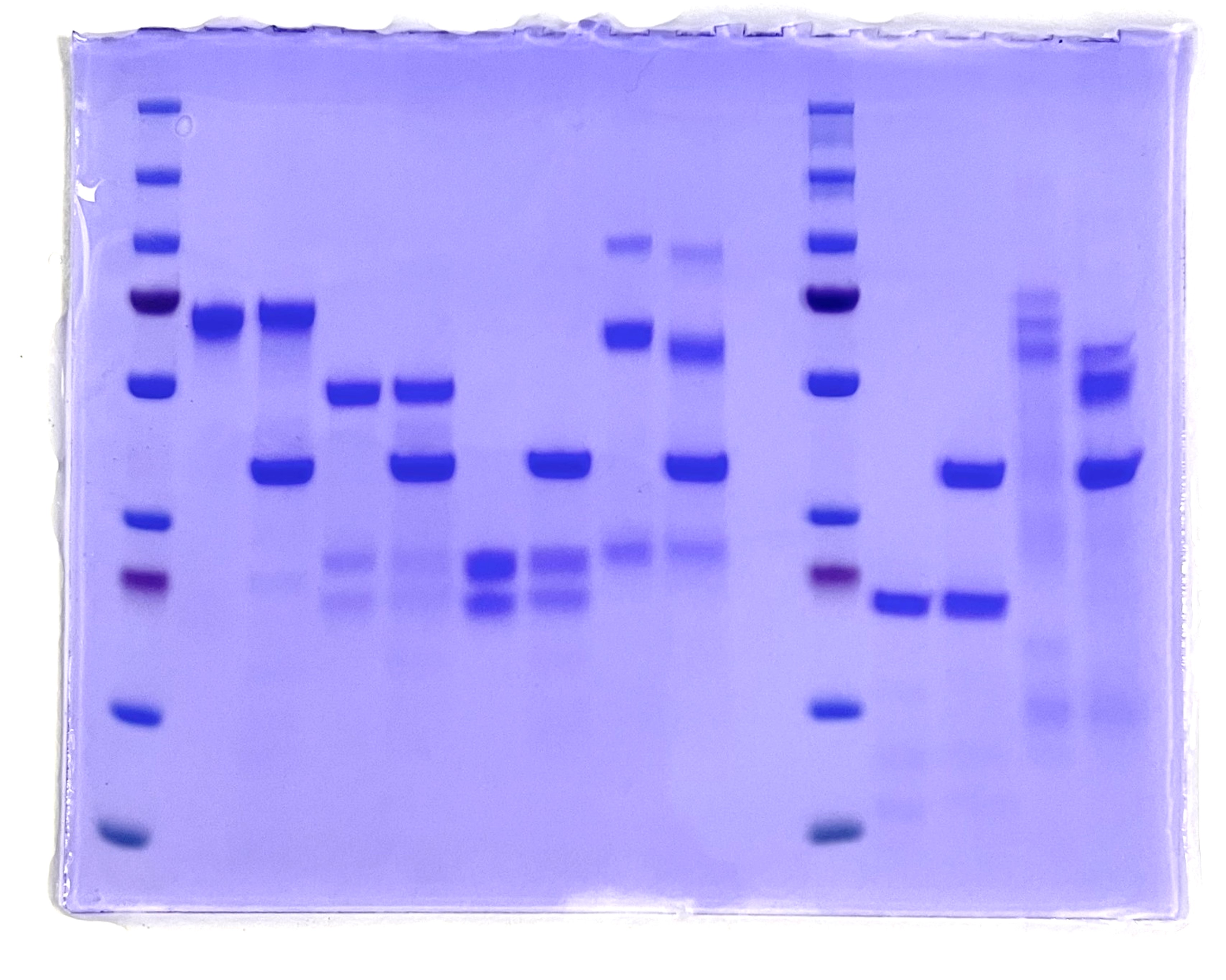

Supplement: Figure 3—figure supplement 5—source data 1. [file elife-109112-fig3-figsupp5-data1.zip › Figure 3 supplement 5 - source data 1/Figure 3 supplement 5- source data 1/Figure 3 supplement 5 - source data 1.jpg]

Figure S8

Glycosylation test of recombinant SVMPs

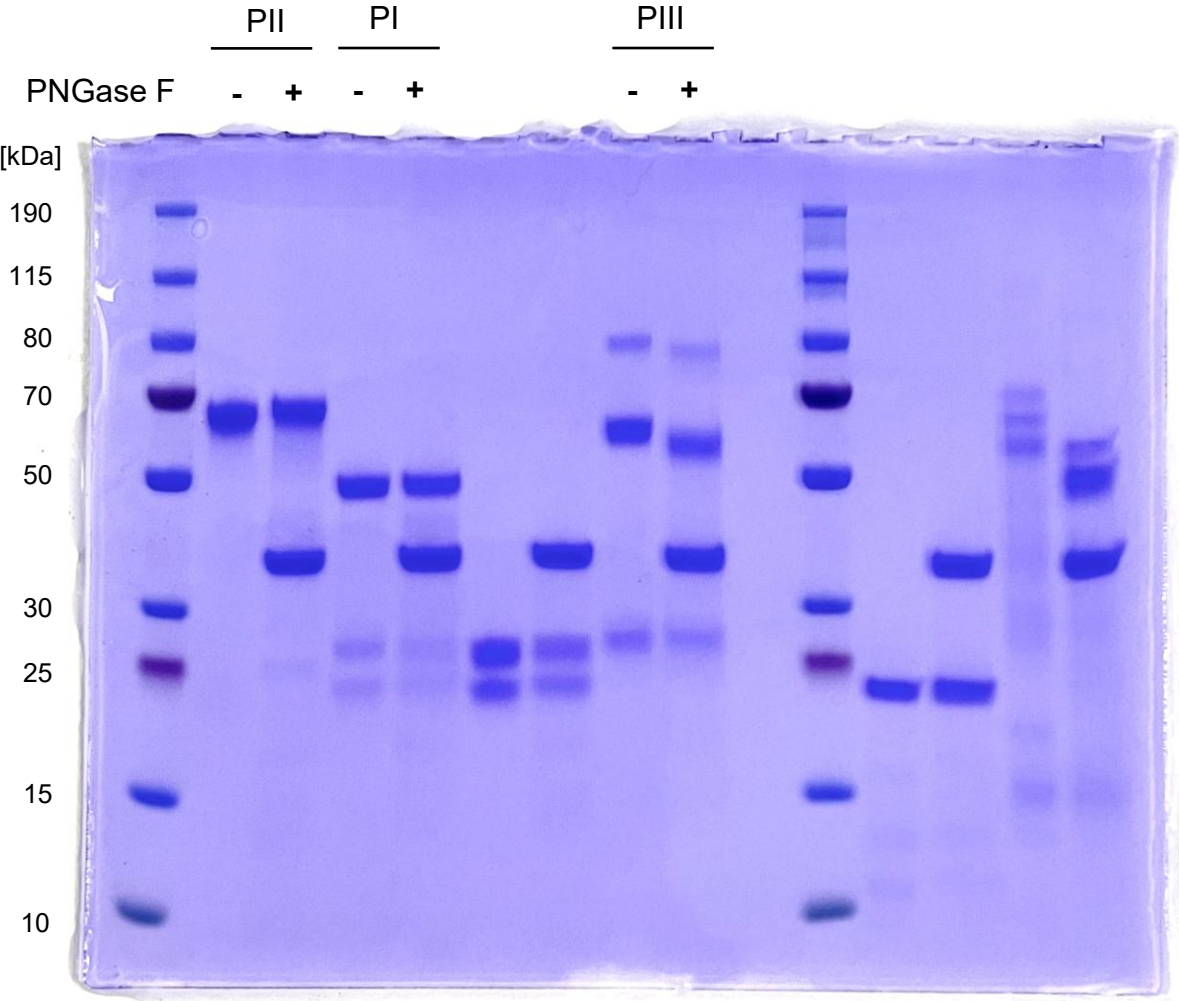

Supplement: Figure 3—figure supplement 5—source data 2. [file elife-109112-fig3-figsupp5-data2.zip › Figure 3 supplement 5 - source data 2/Figure 3 supplement 5 - source data 2.pdf]

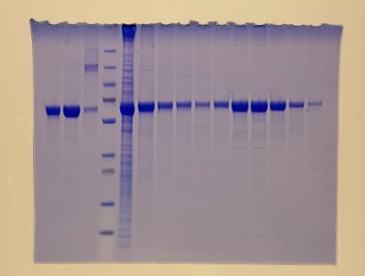

Supplement: Figure 3—figure supplement 6—source data 1. [file elife-109112-fig3-figsupp6-data1.zip › Figure 3 supplement 6 - source data 1/Figure 3 supplement 6 - source data 1/Figure 3 supplement 6b - source data 1.jpg]

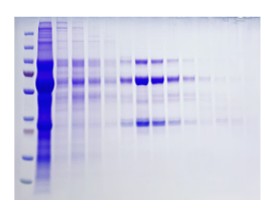

Supplement: Figure 3—figure supplement 6—source data 1. [file elife-109112-fig3-figsupp6-data1.zip › Figure 3 supplement 6 - source data 1/Figure 3 supplement 6 - source data 1/Figure 3 supplement 6c - source data 1.jpg]

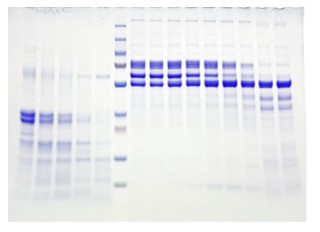

Supplement: Figure 3—figure supplement 6—source data 1. [file elife-109112-fig3-figsupp6-data1.zip › Figure 3 supplement 6 - source data 1/Figure 3 supplement 6 - source data 1/Figure 3 supplement 6d - source data 1.1.jpg]

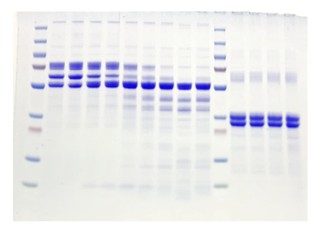

Supplement: Figure 3—figure supplement 6—source data 1. [file elife-109112-fig3-figsupp6-data1.zip › Figure 3 supplement 6 - source data 1/Figure 3 supplement 6 - source data 1/Figure 3 supplement 6d - source data 1.2.jpg]

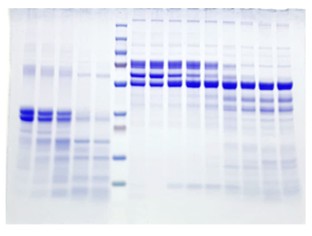

Supplement: Figure 3—figure supplement 6—source data 1. [file elife-109112-fig3-figsupp6-data1.zip › Figure 3 supplement 6 - source data 1/Figure 3 supplement 6 - source data 1/Figure 3 supplement 6d - source data 1.3.jpg]

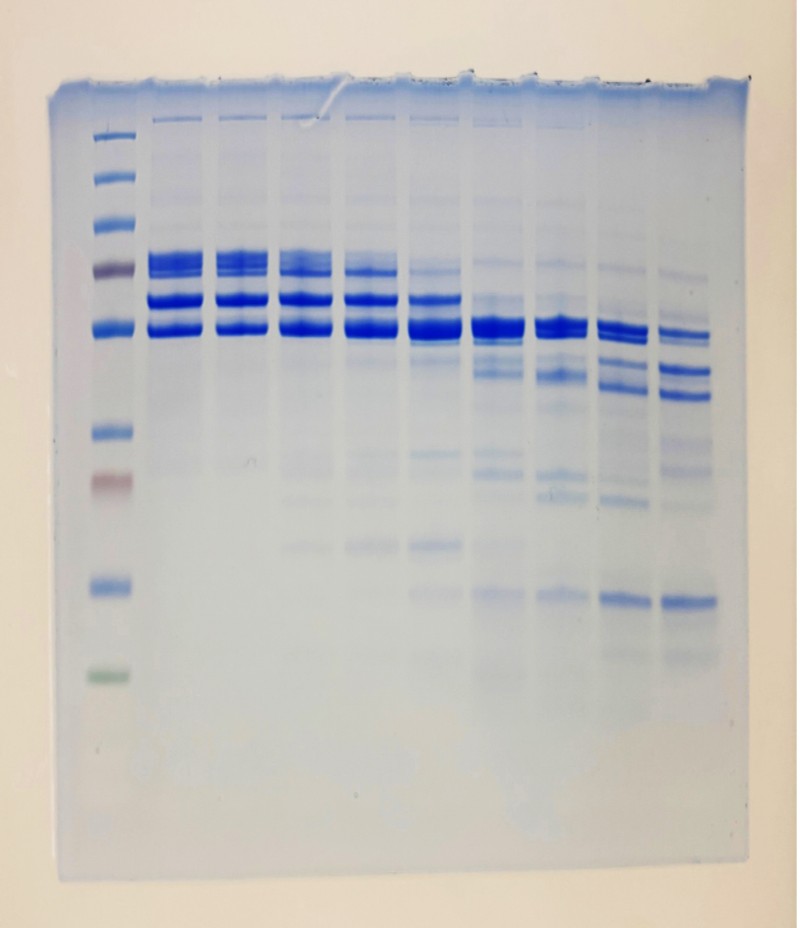

Supplement: Figure 3—figure supplement 6—source data 1. [file elife-109112-fig3-figsupp6-data1.zip › Figure 3 supplement 6 - source data 1/Figure 3 supplement 6 - source data 1/Figure 3 supplement 6e - source data 1.1.jpg]

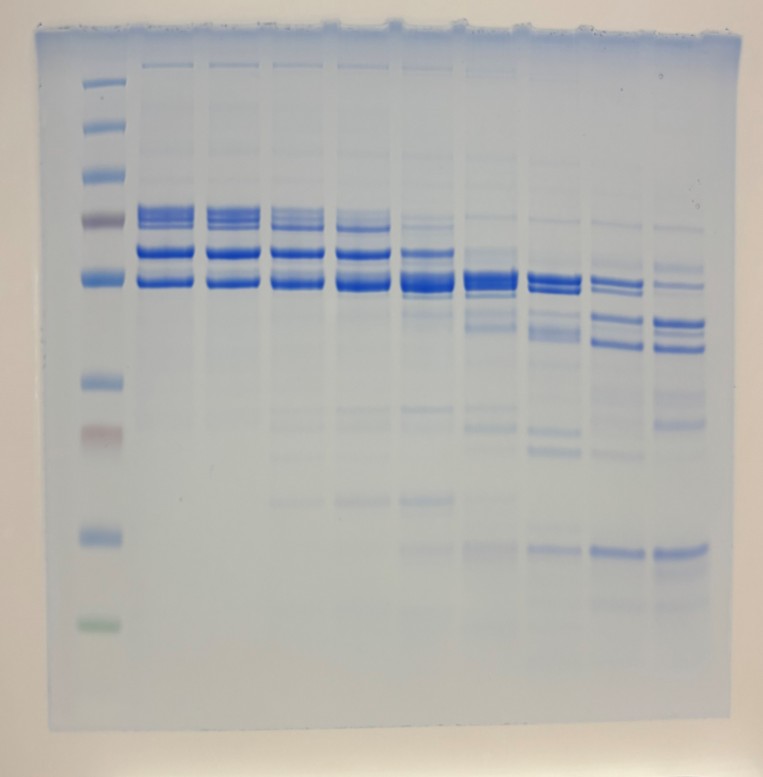

Supplement: Figure 3—figure supplement 6—source data 1. [file elife-109112-fig3-figsupp6-data1.zip › Figure 3 supplement 6 - source data 1/Figure 3 supplement 6 - source data 1/Figure 3 supplement 6e - source data 1.2.jpg]

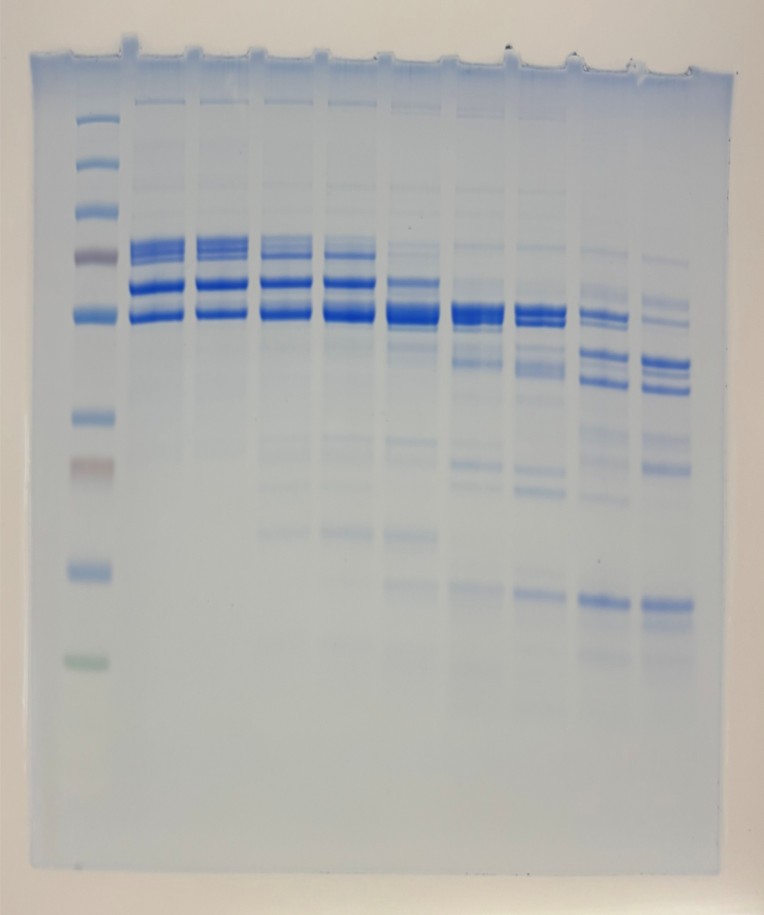

Supplement: Figure 3—figure supplement 6—source data 1. [file elife-109112-fig3-figsupp6-data1.zip › Figure 3 supplement 6 - source data 1/Figure 3 supplement 6 - source data 1/Figure 3 supplement 6e - source data 1.3.jpg]

Figure S10b

Coomassie-stained SDS gel of activated PI zym  $\Delta$ C SEC

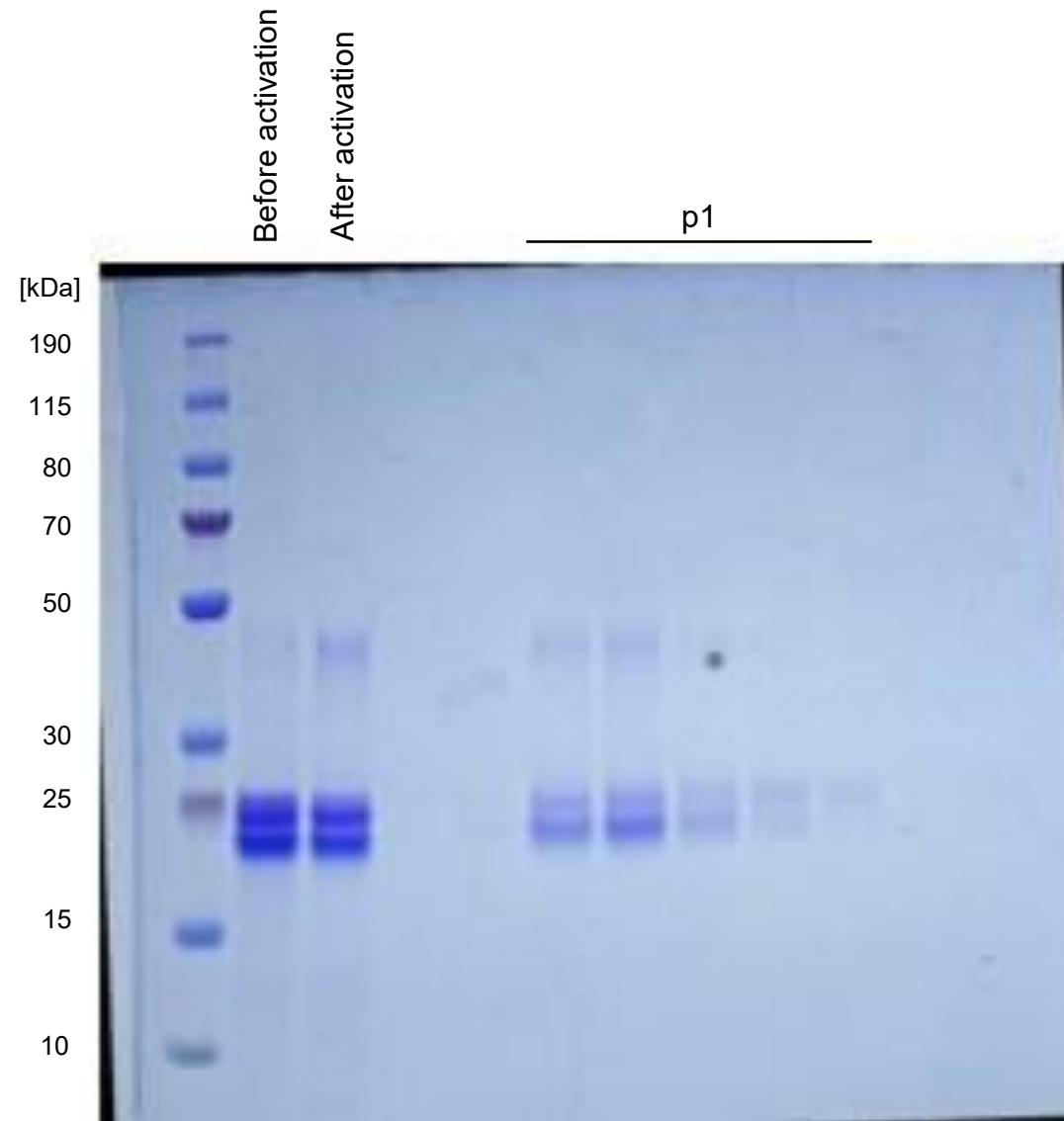

Supplement: Figure 3—figure supplement 7—source data 2. [file elife-109112-fig3-figsupp7-data2.zip › Figure 3 supplement 7 - source data 2/Figure 3 supplement 7 - source data 2.pdf]

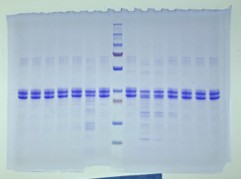

Supplement: Figure 4—source data 1. [file elife-109112-fig4-data1.zip › Figure 4 - source data 1/Figure 4b - source data 1.jpg]

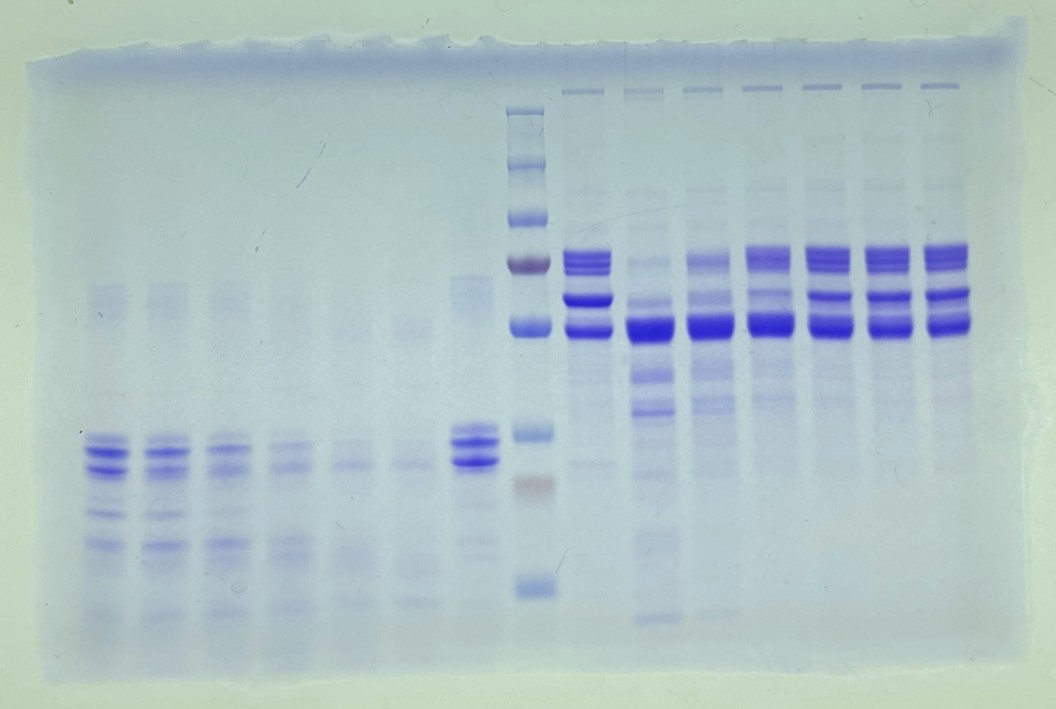

Supplement: Figure 4—source data 1. [file elife-109112-fig4-data1.zip › Figure 4 - source data 1/Figure 4c - source data 1.jpg]

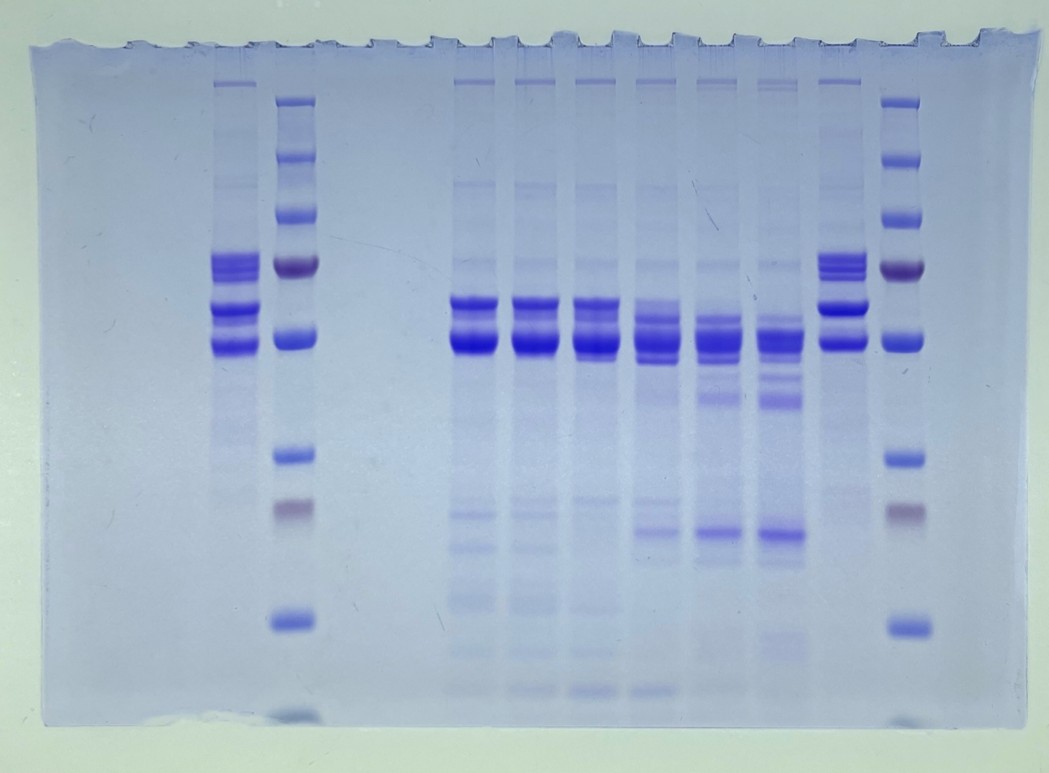

Supplement: Figure 4—source data 1. [file elife-109112-fig4-data1.zip › Figure 4 - source data 1/Figure 4d - source data 1.jpg]

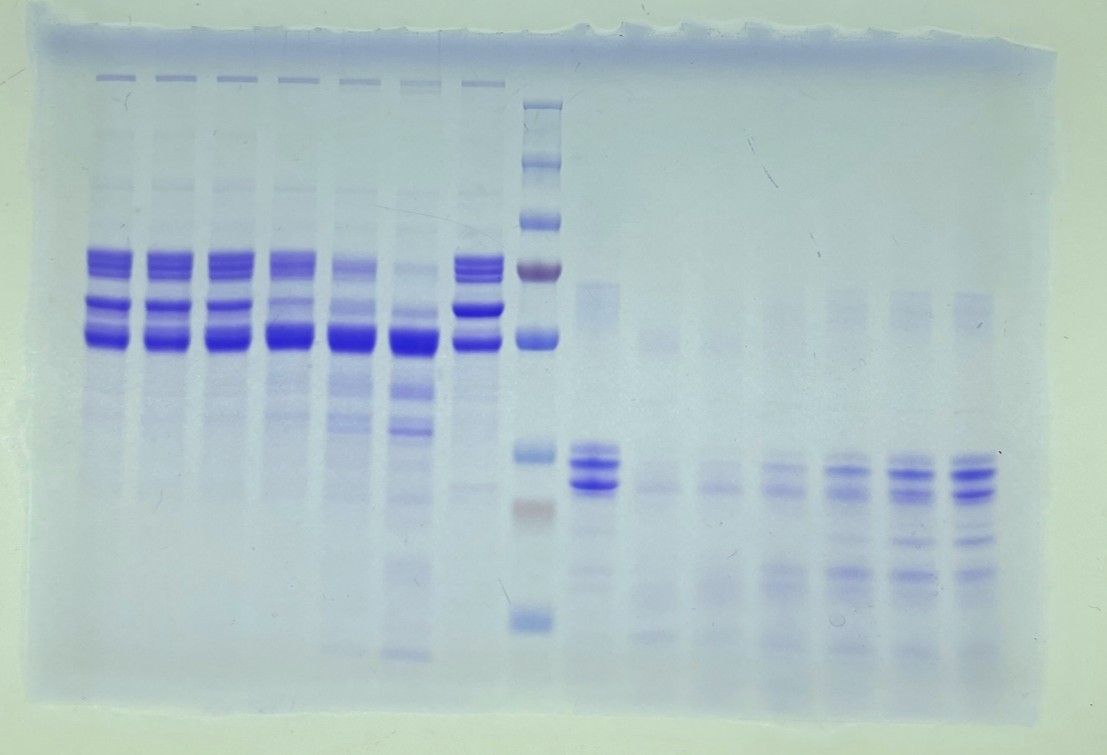

Supplement: Figure 4—source data 1. [file elife-109112-fig4-data1.zip › Figure 4 - source data 1/Figure 4e - source data 1.jpg]

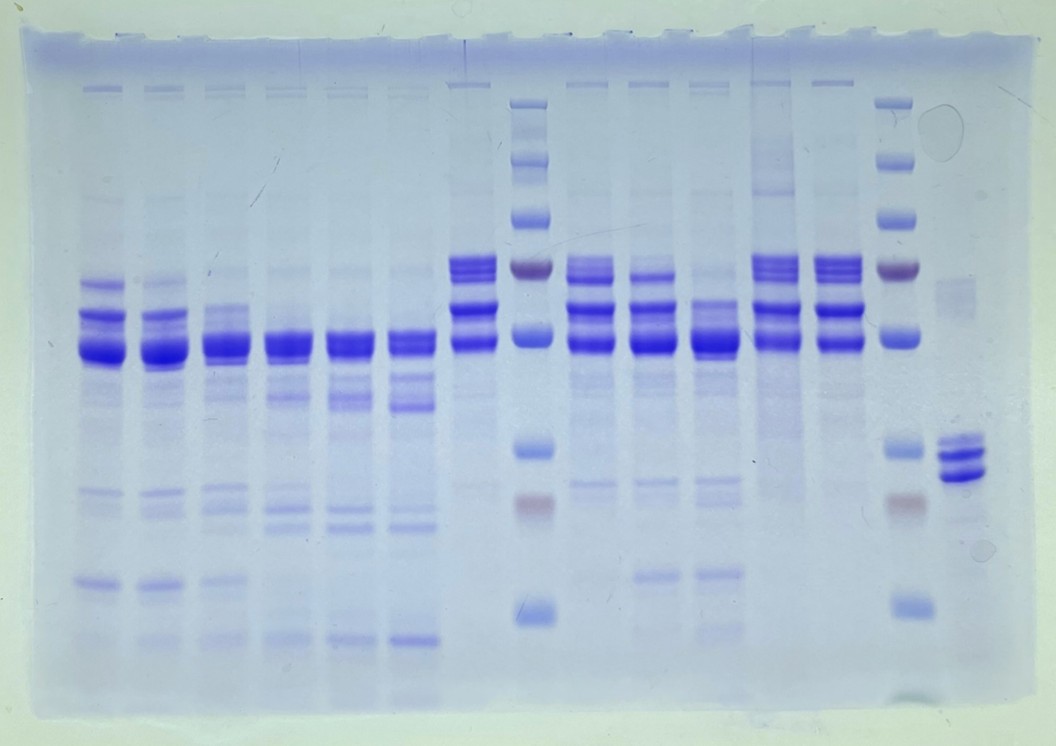

Supplement: Figure 4—source data 1. [file elife-109112-fig4-data1.zip › Figure 4 - source data 1/Figure 4f - source data 1.jpg]
