# Supplementary material for: Purified zymogens reveal mechanisms of snake venom metalloproteinase auto-activation
Source: eLife. 2026 Jun 10;15:RP109112. doi: 10.7554/eLife.109112 (PMC13252954; doi:10.7554/eLife.109112)

Figure S2a 1

Expression of mature SVMP PI

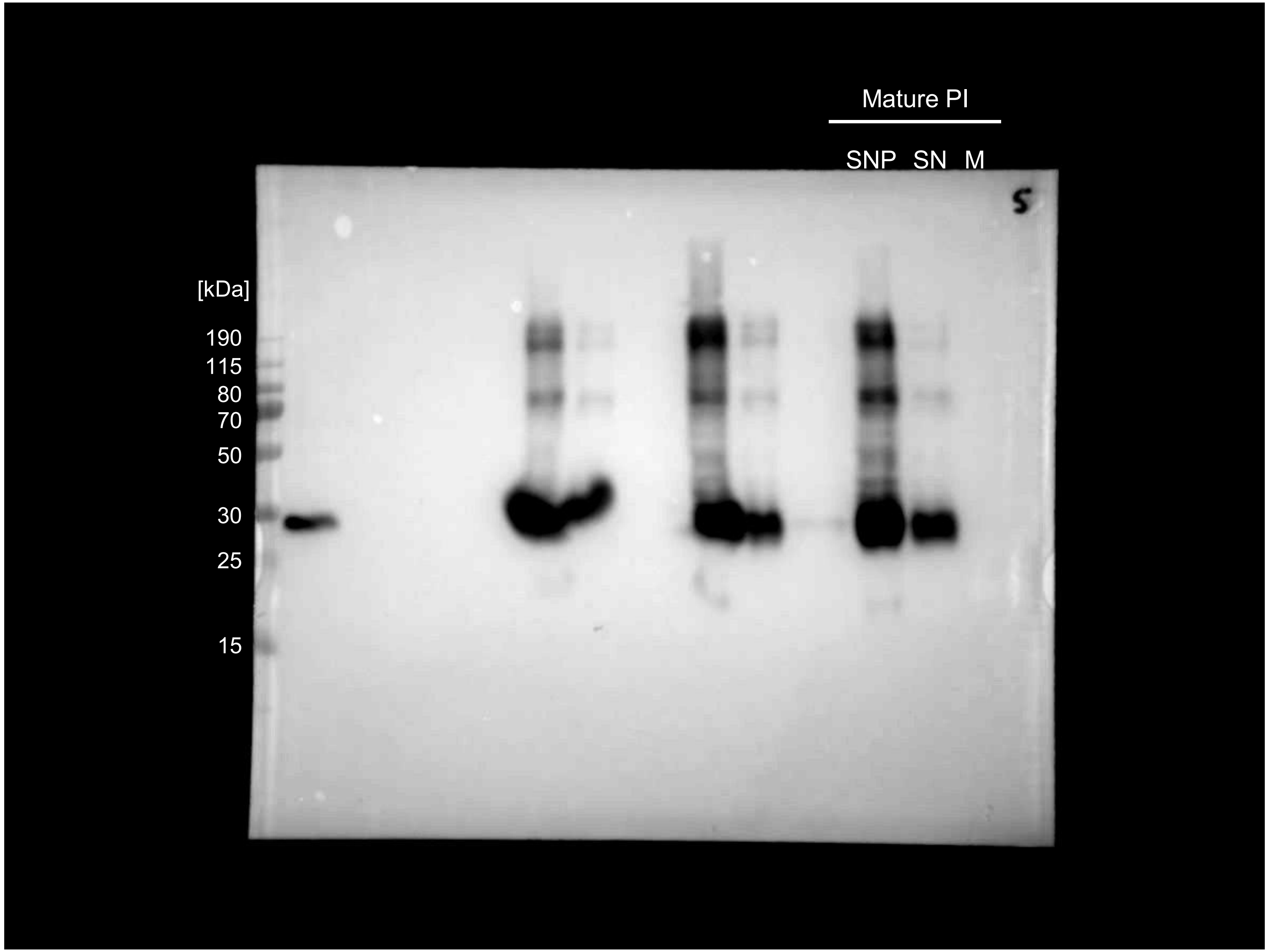

Figure S2a 2

Expression of mature SVMP PIII

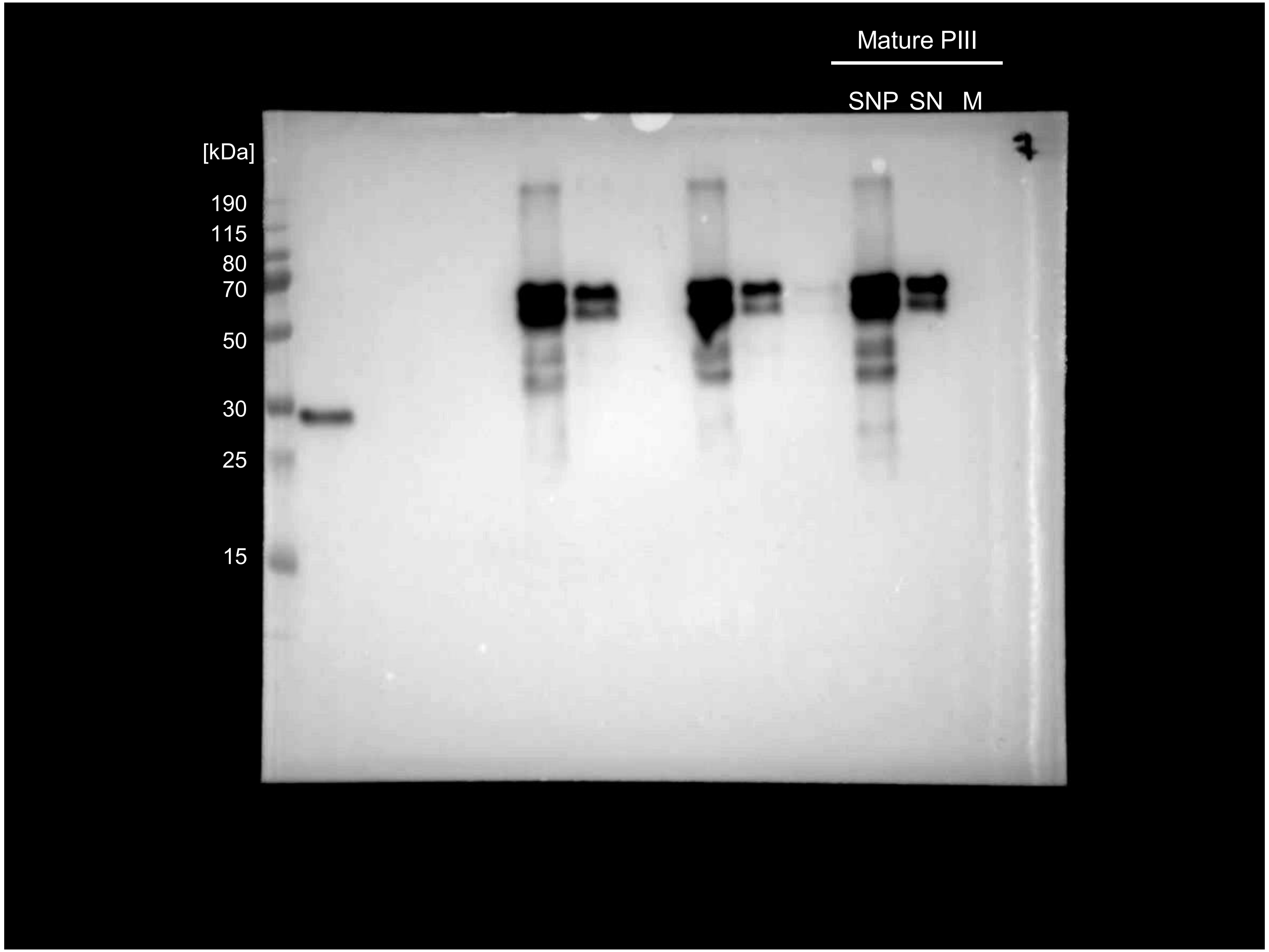

Figure S2b

Expression of SVMP7 in the presence of Marimastat

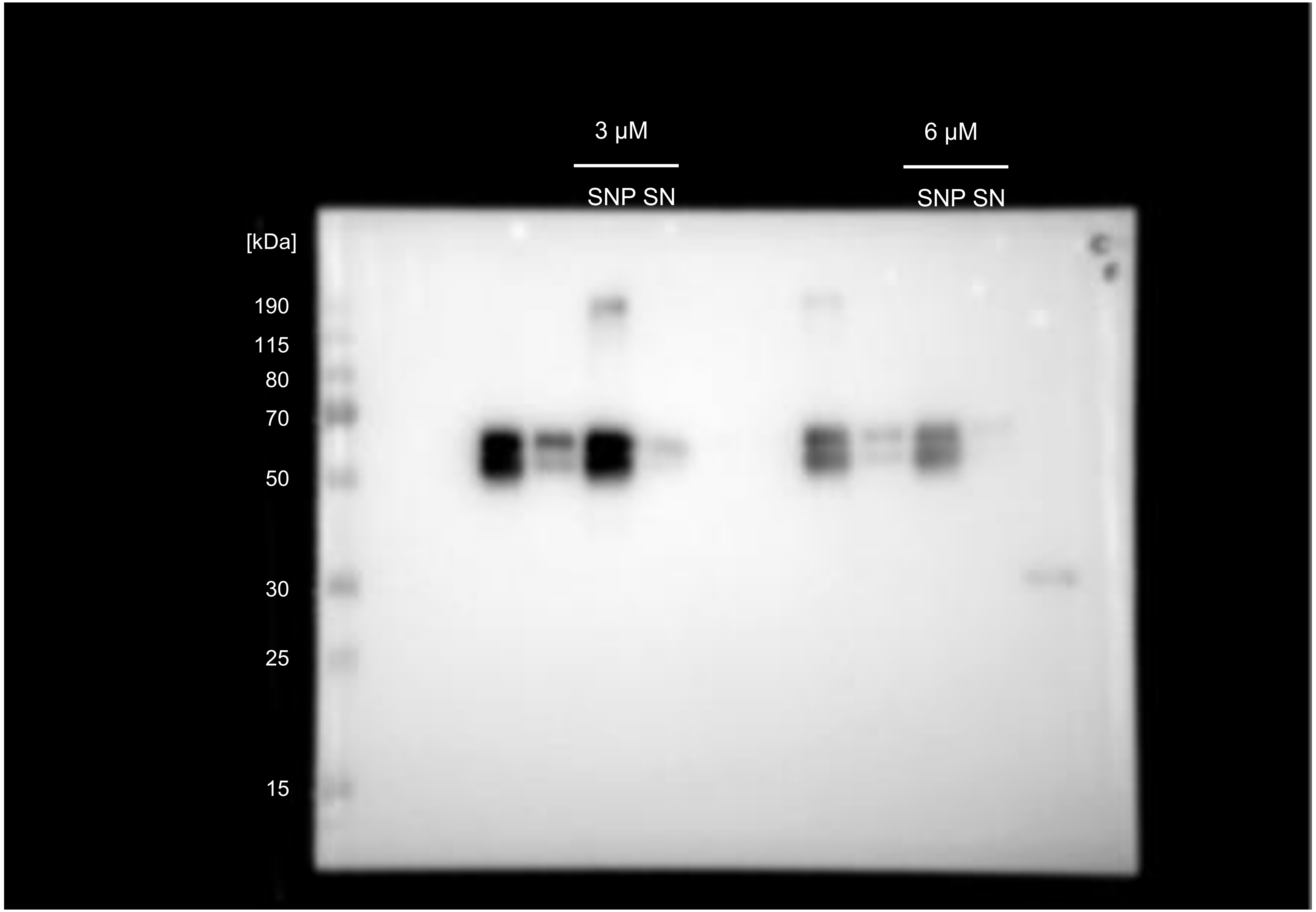

Figure S2c

Expression of SVMP active site mutants

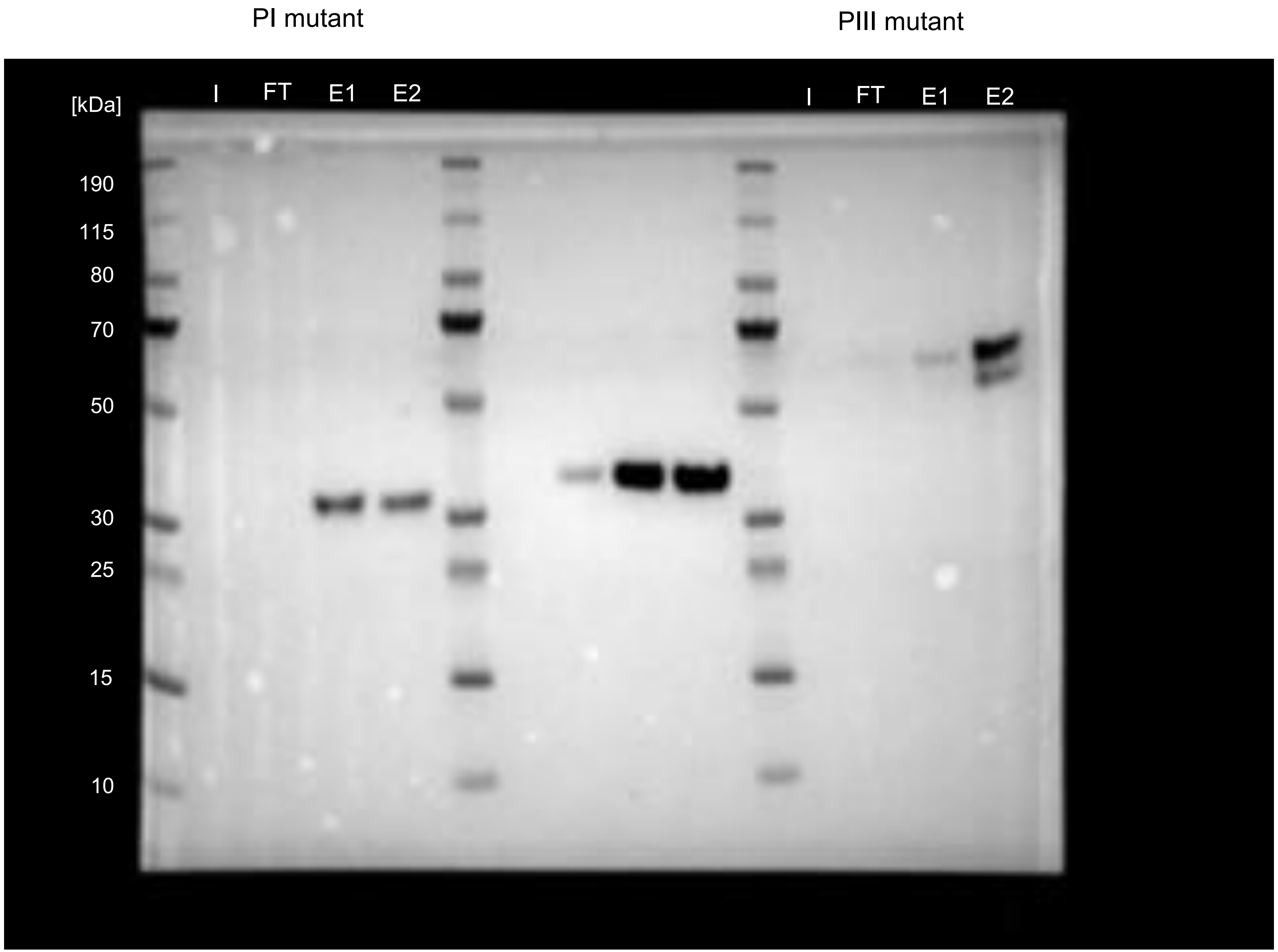

Figure S2d

Expression of propeptide-SVMP fusion protein

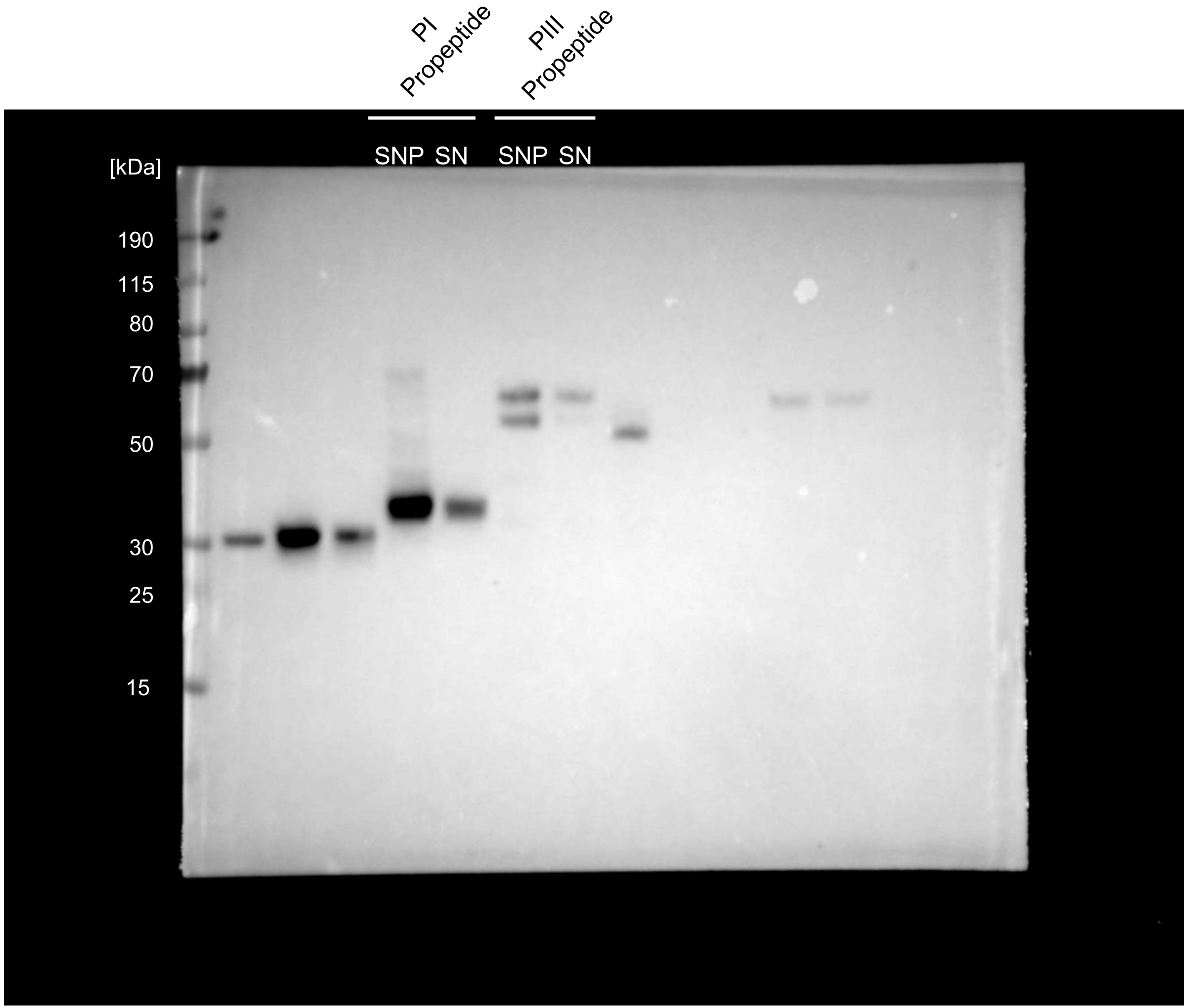

Supplement: Figure 2—figure supplement 1—source data 2. [file elife-109112-fig2-figsupp1-data2.zip › Figure 2 supplement 1 - source data 2/Figure 2 supplement 1 - source data 2.pdf]
