# Supplementary material for: Purified zymogens reveal mechanisms of snake venom metalloproteinase auto-activation
Source: eLife. 2026 Jun 10;15:RP109112. doi: 10.7554/eLife.109112 (PMC13252954; doi:10.7554/eLife.109112)

Figure 3a

SVMP PI zymogen SEC – SDS-PAGE gel

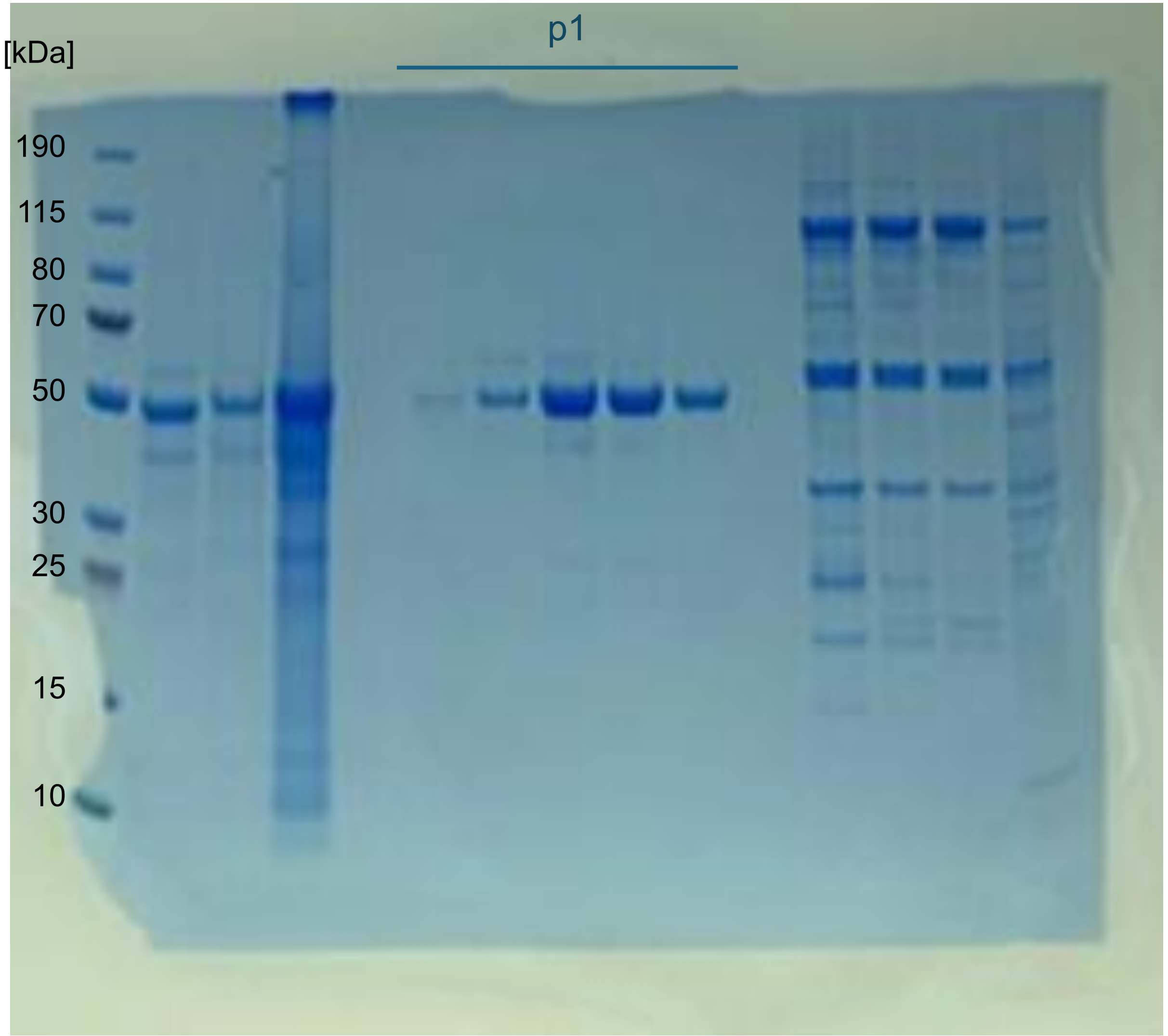

Figure 3b

SVMP PII zymogen SEC – SDS-PAGE

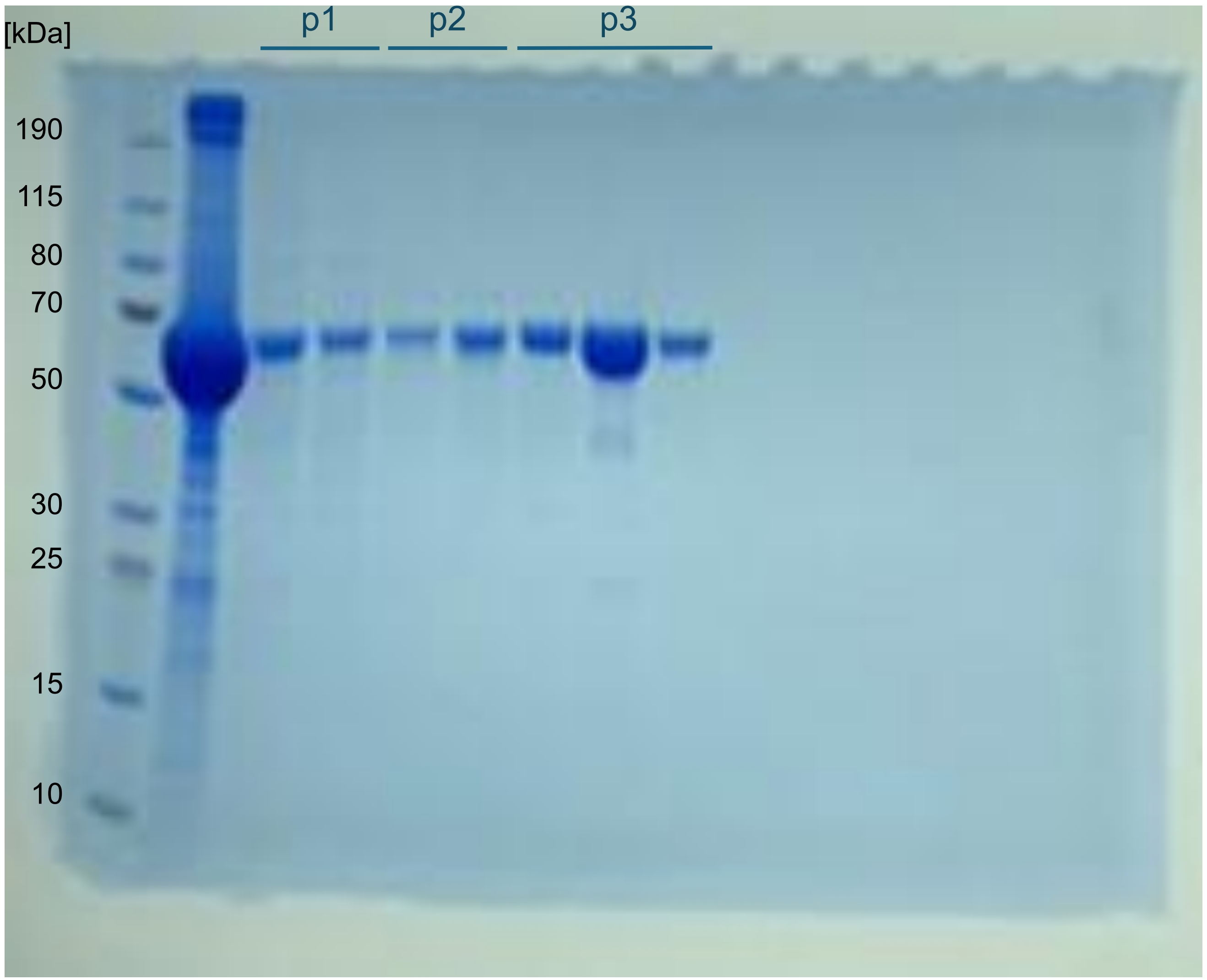

Figure 3c

SVMP PIII zymogen SEC – SDS-PAGE

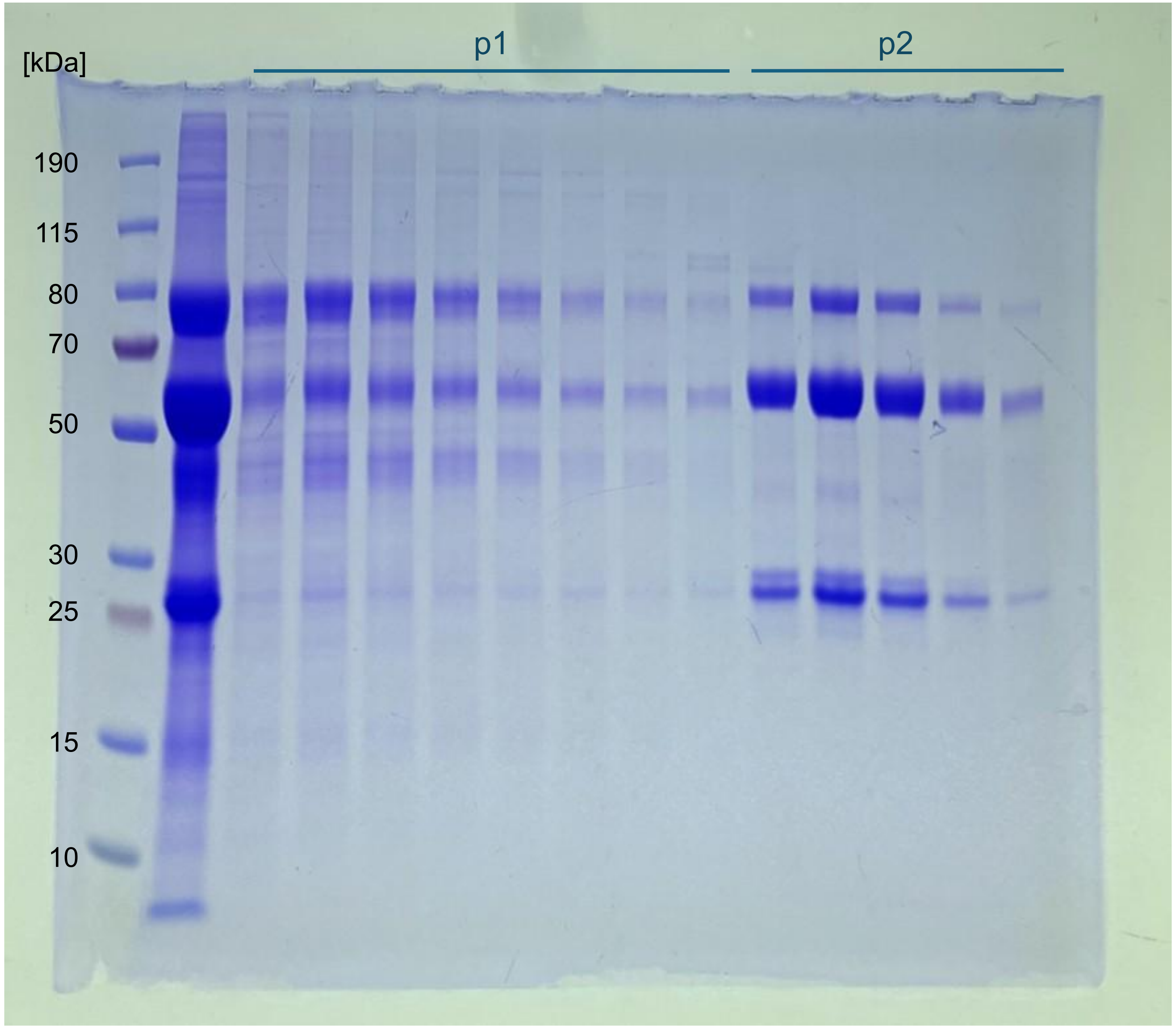

Figure 3d

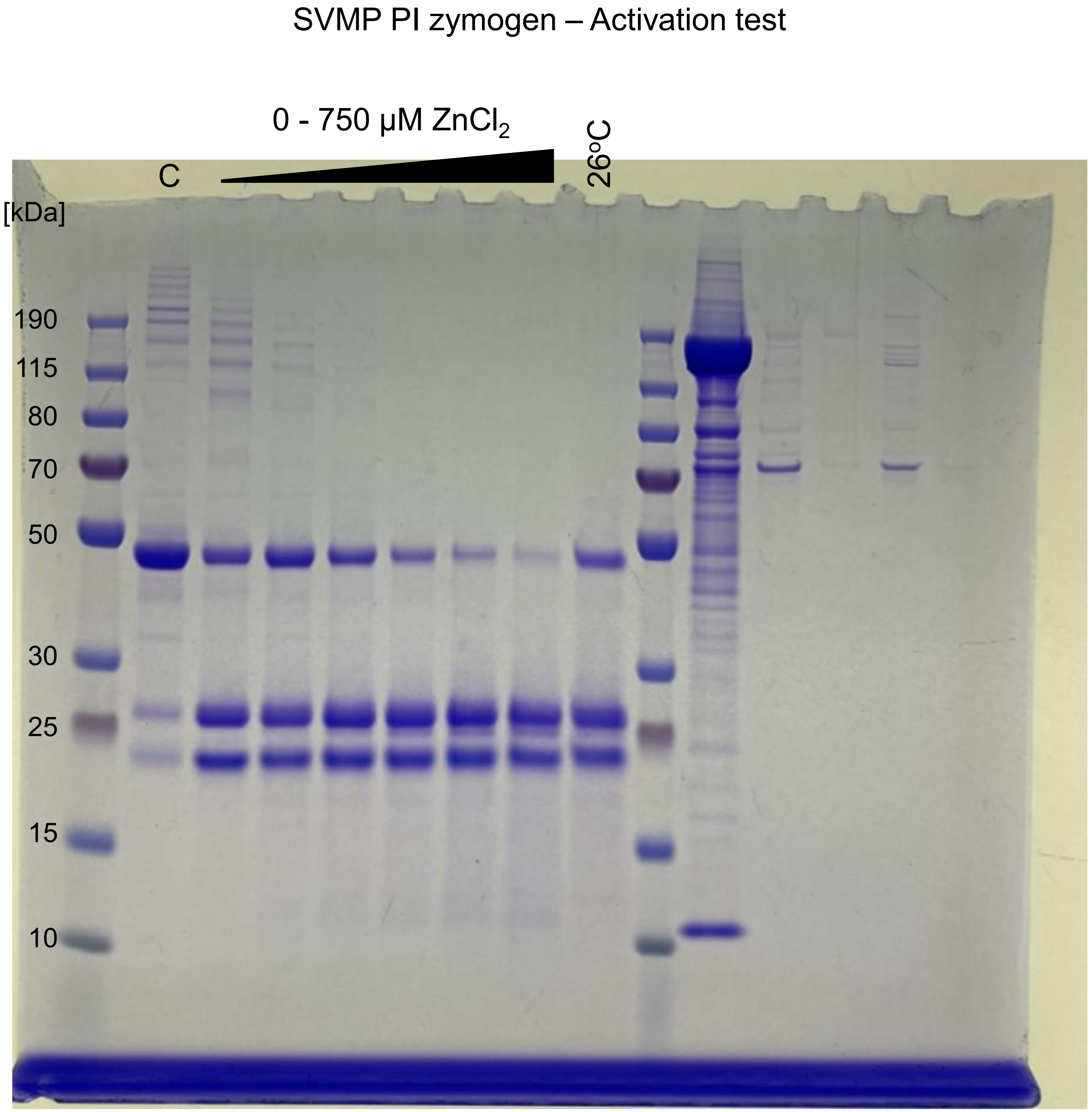

SVMP PI zymogen – Activation test
0 - 750 µM ZnCl2
C
26°C
[kDa]
190
115
80
70
50
30
25
15
10

Figure 3e

SVMP PII zymogen – Activation test

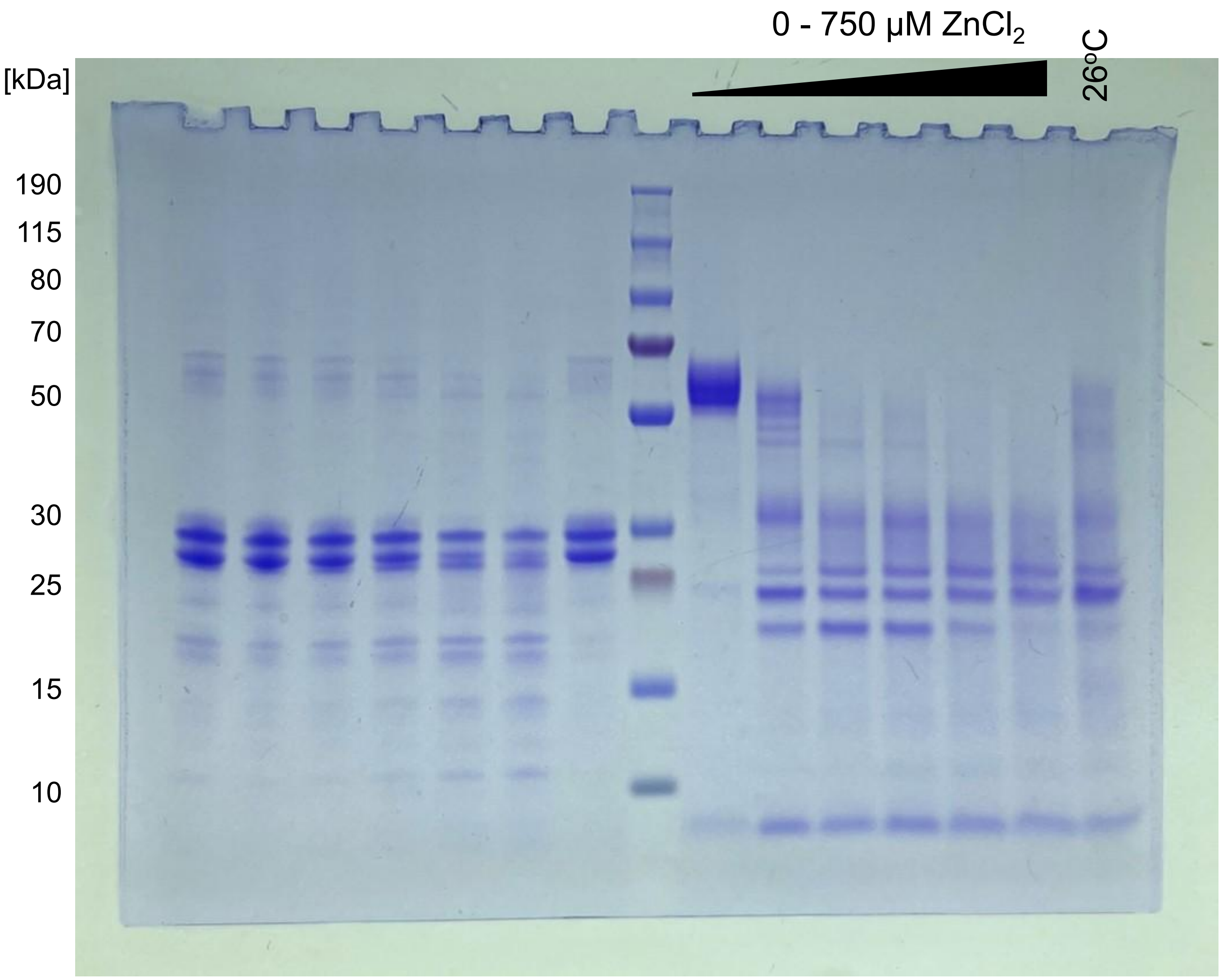

Figure 3f

SVMP PIII zymogen – Activation test

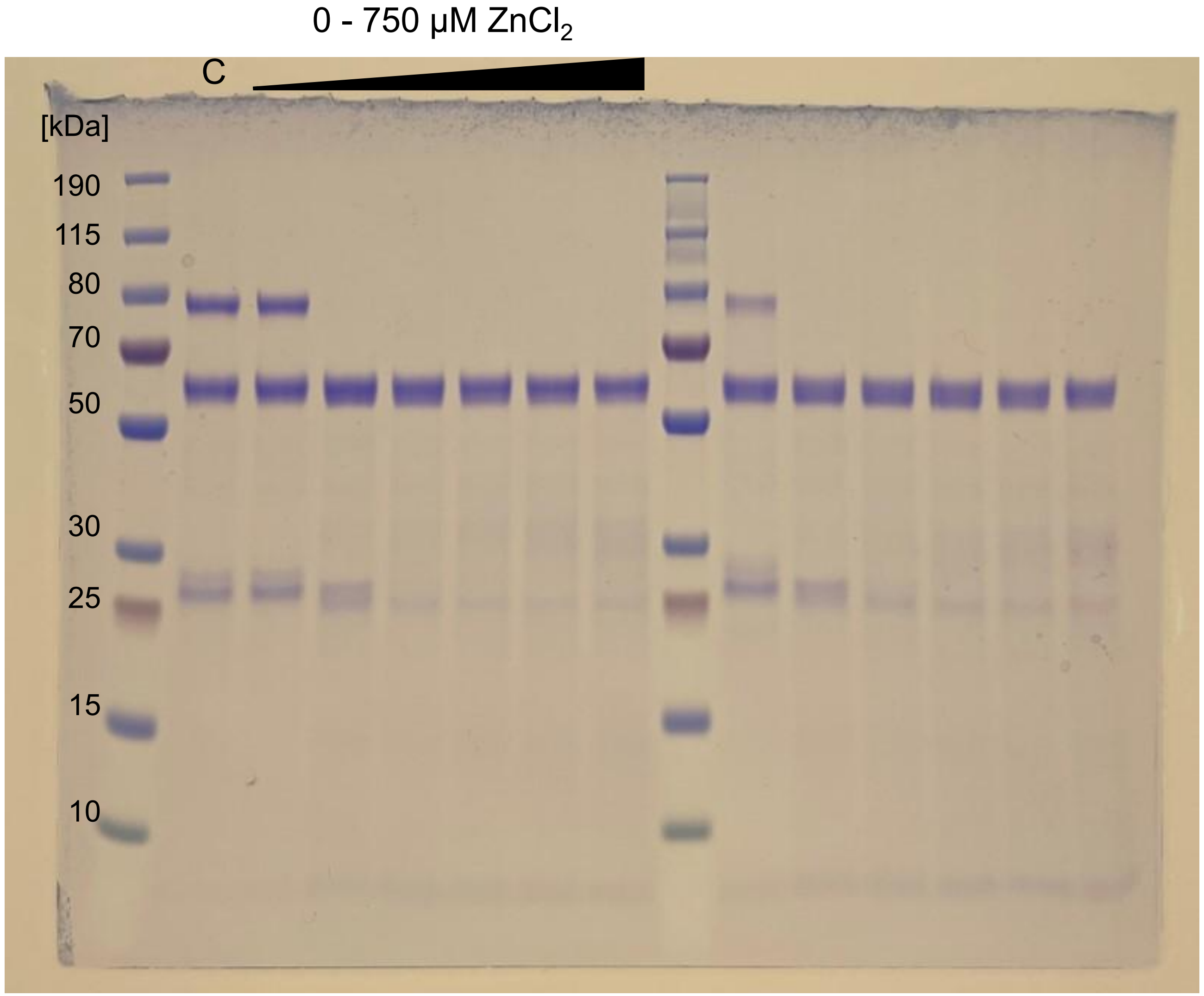

Supplement: Figure 3—source data 2. [file elife-109112-fig3-data2.zip › Figure 3 - source data 2.pdf]
