# Supplementary material for: Purified zymogens reveal mechanisms of snake venom metalloproteinase auto-activation
Source: eLife. 2026 Jun 10;15:RP109112. doi: 10.7554/eLife.109112 (PMC13252954; doi:10.7554/eLife.109112)

Figure S4a

PI SVMP C-terminal auto-cleavage before and after dialysis, following IMAC purification - SDS-PAGE

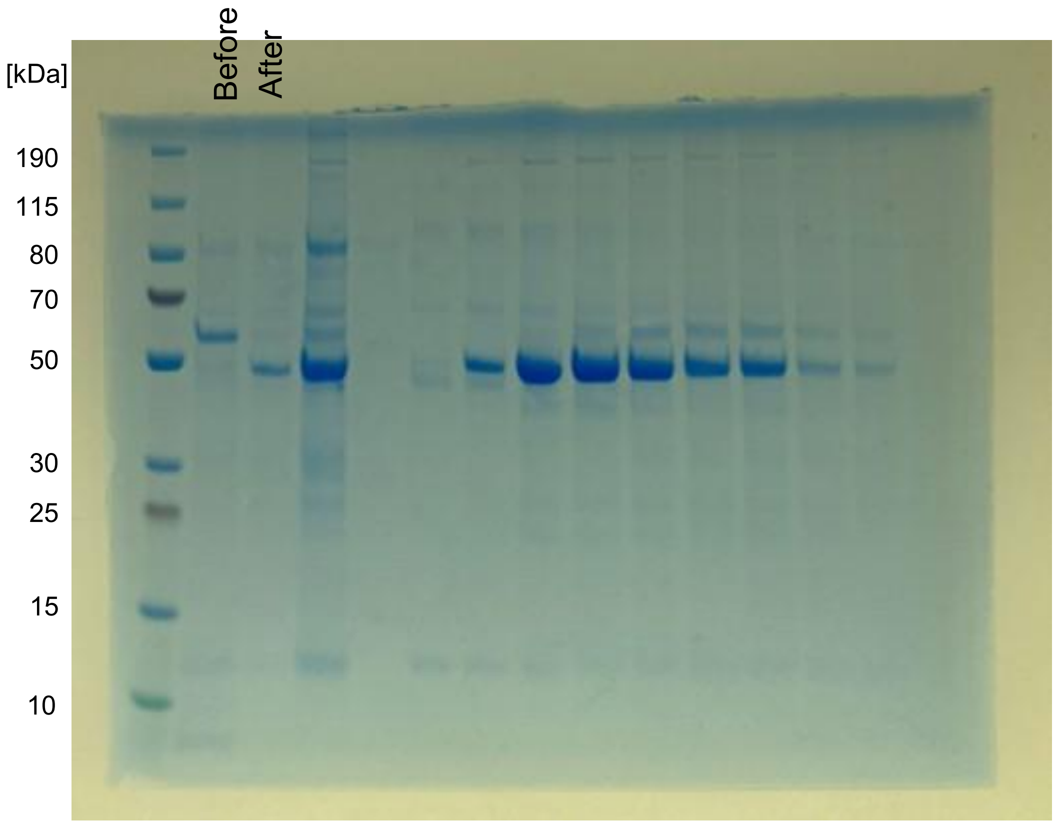

Figure S4b

PI SVMP C-terminal auto-cleavage before and after dialysis, following IMAC purification - WB

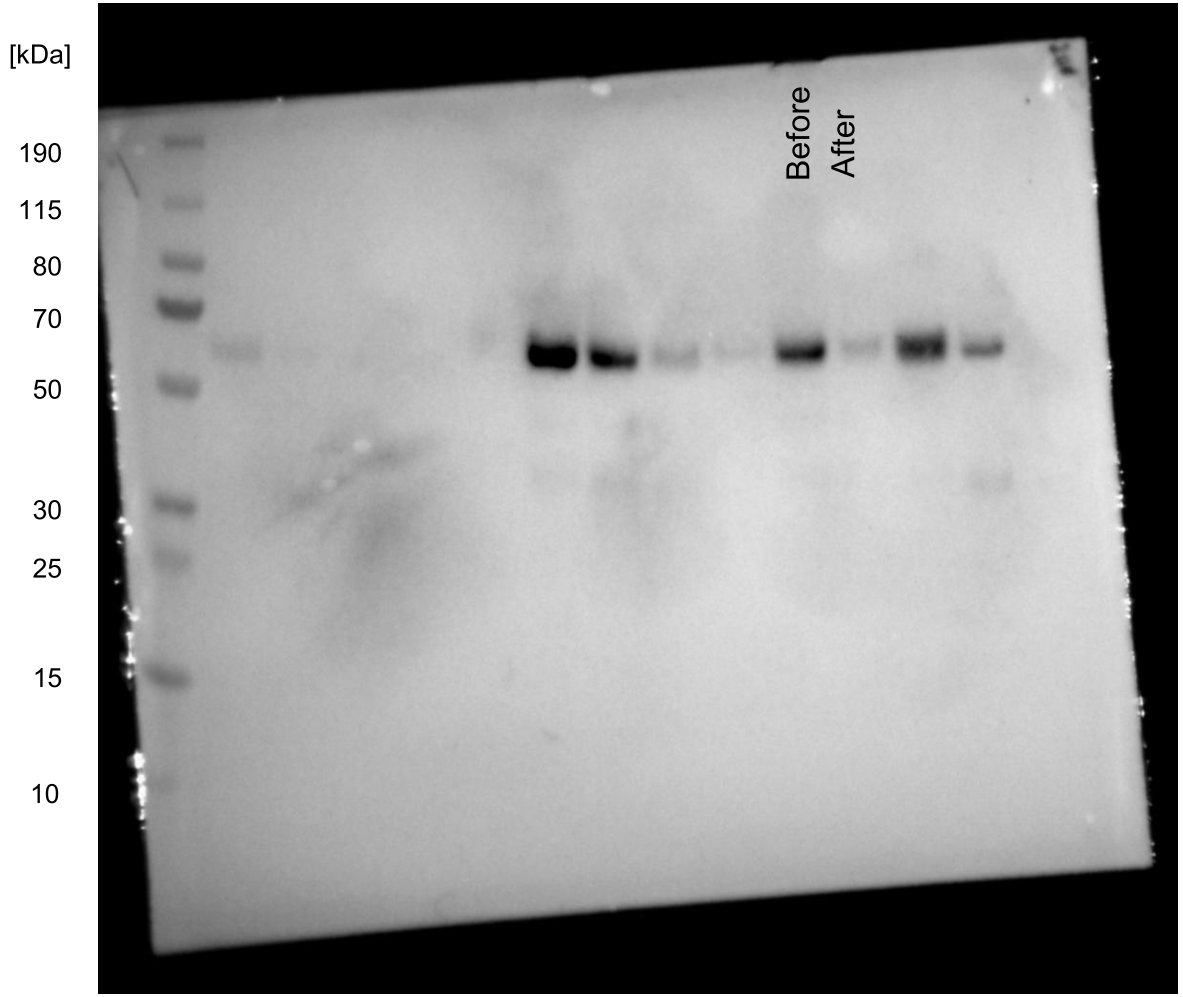

Supplement: Figure 3—figure supplement 1—source data 2. [file elife-109112-fig3-figsupp1-data2.zip › Figure 3 supplement 1 - source data 2/Figure 3 supplment 1 - source data 2.pdf]
