# Supplementary material for: Purified zymogens reveal mechanisms of snake venom metalloproteinase auto-activation
Source: eLife. 2026 Jun 10;15:RP109112. doi: 10.7554/eLife.109112 (PMC13252954; doi:10.7554/eLife.109112)

Figure S5a

Reducing SDS-PAGE of IMAC purification of PIΔC SVMP

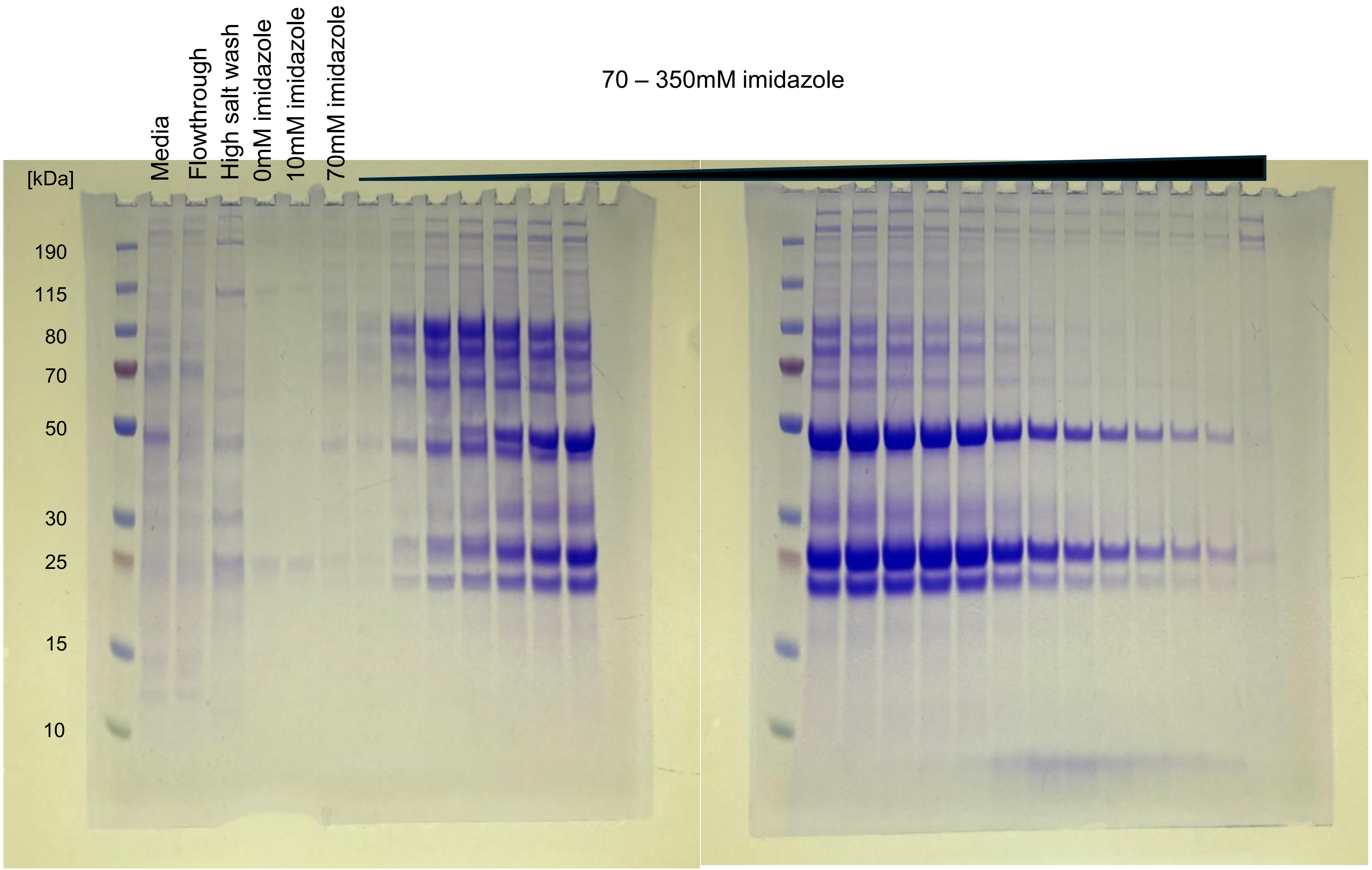

Figure S5b

Reducing SDS-PAGE of IMAC purification of PIII SVMP

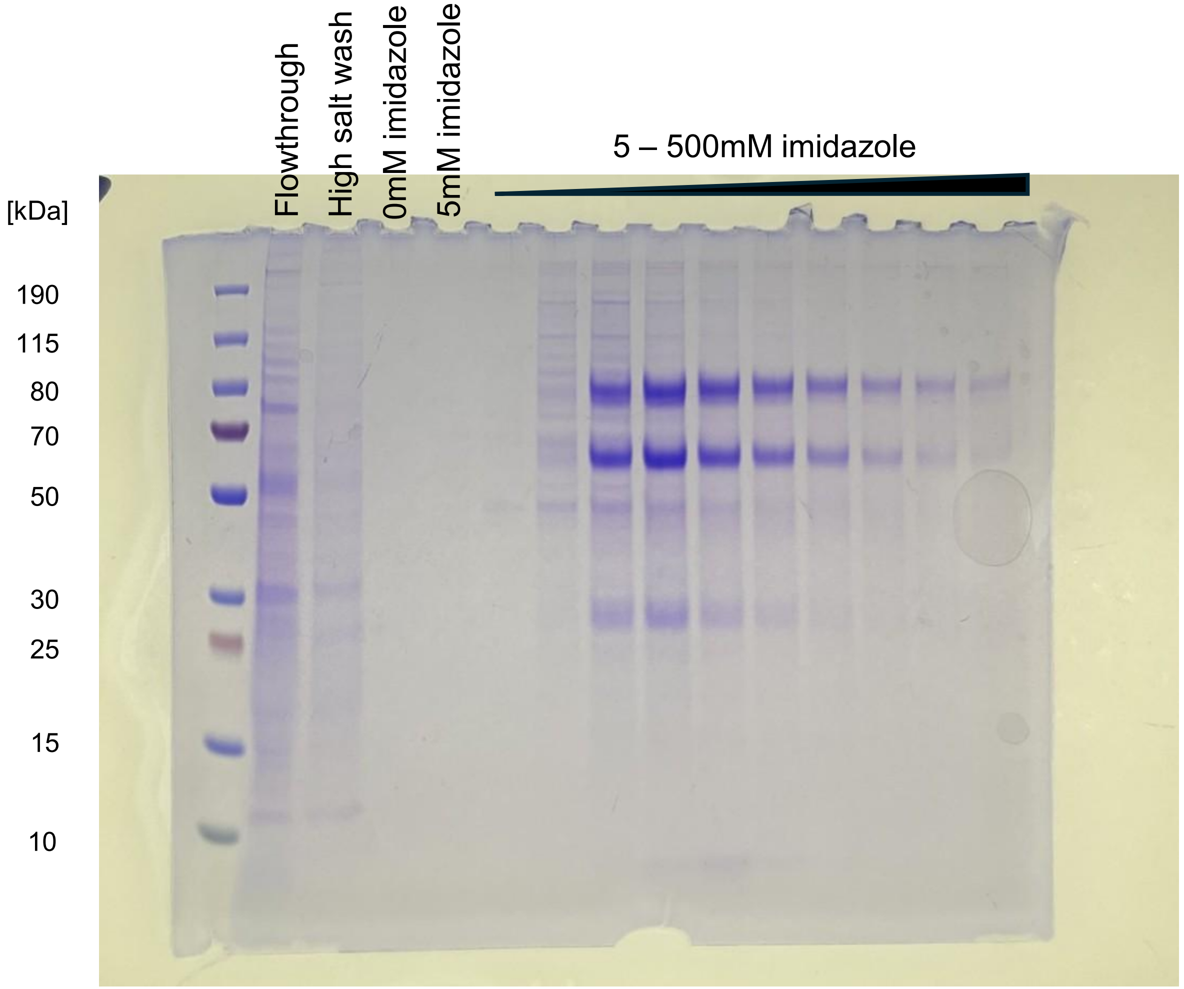

Supplement: Figure 3—figure supplement 2—source data 2. [file elife-109112-fig3-figsupp2-data2.zip › Figure 3 supplement 2- source data 2/Figure 3 supplement 2- source data 2.pdf]
