# Supplementary material for: Purified zymogens reveal mechanisms of snake venom metalloproteinase auto-activation
Source: eLife. 2026 Jun 10;15:RP109112. doi: 10.7554/eLife.109112 (PMC13252954; doi:10.7554/eLife.109112)

Figure S6a

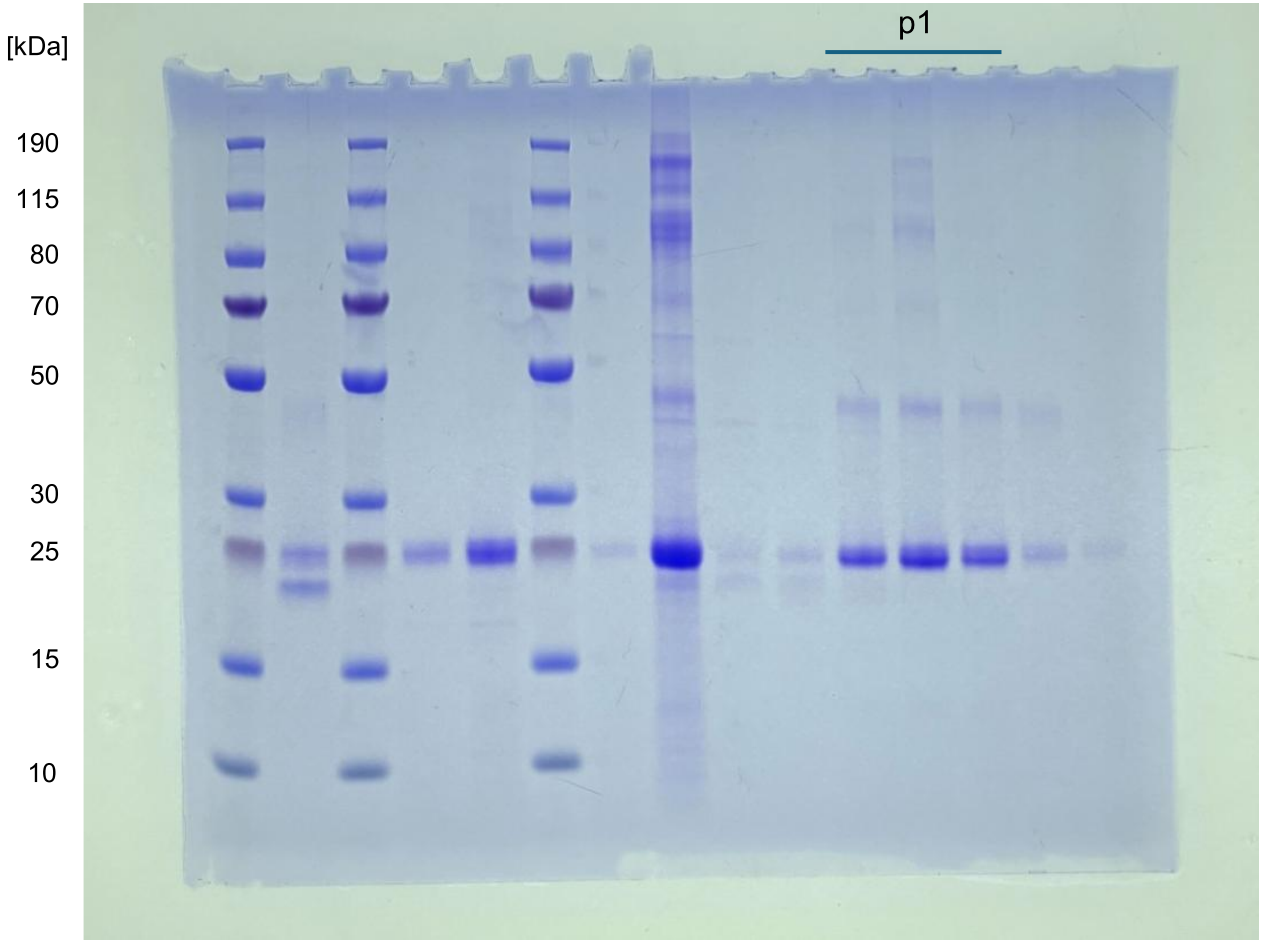

Figure S6b

SDS-PAGE of metalloproteinase domain of PIΔC

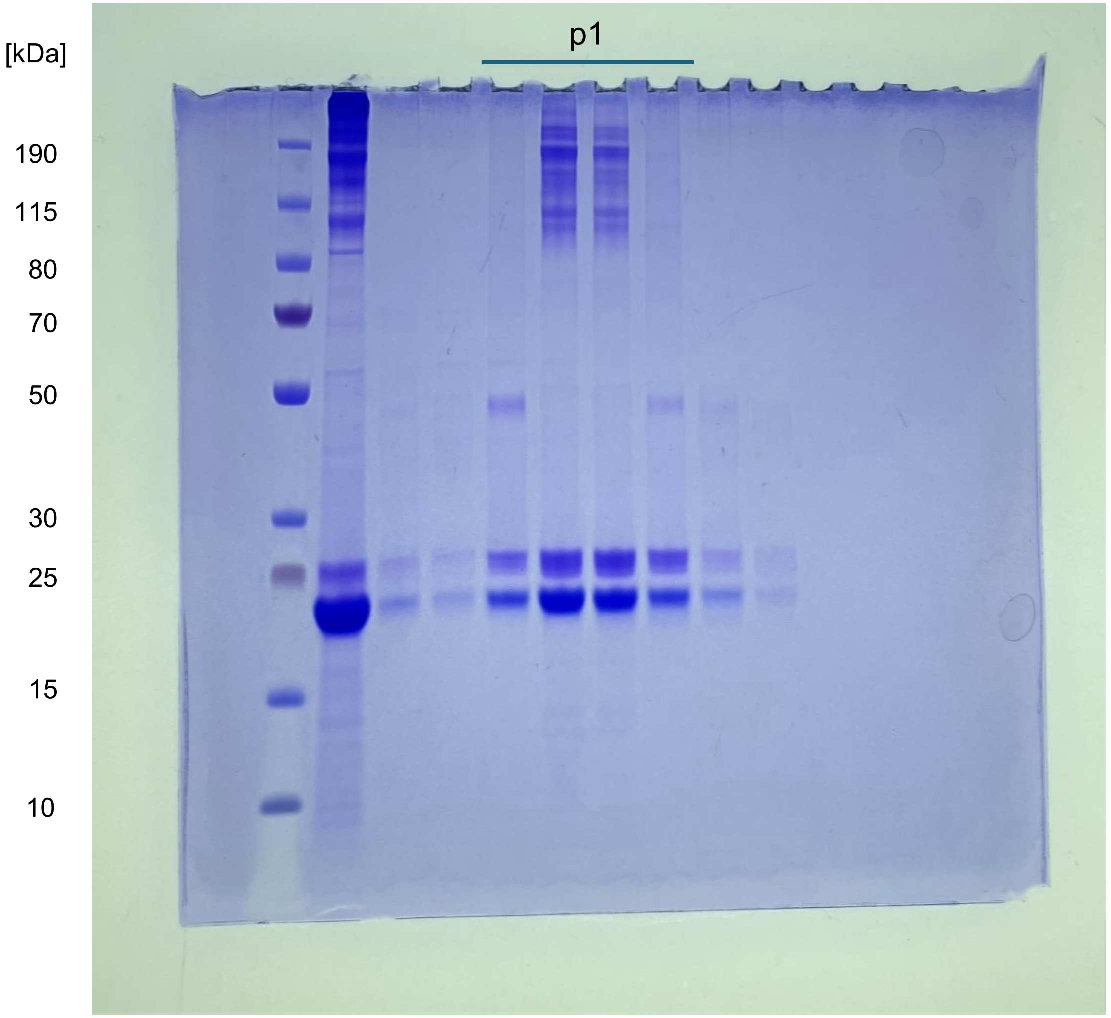

Figure S6c

PIΔC activity against casein with no prior addition of $Zn2+$ ions (left) and with prior incubation with 450 µM of $Zn2+$(right).

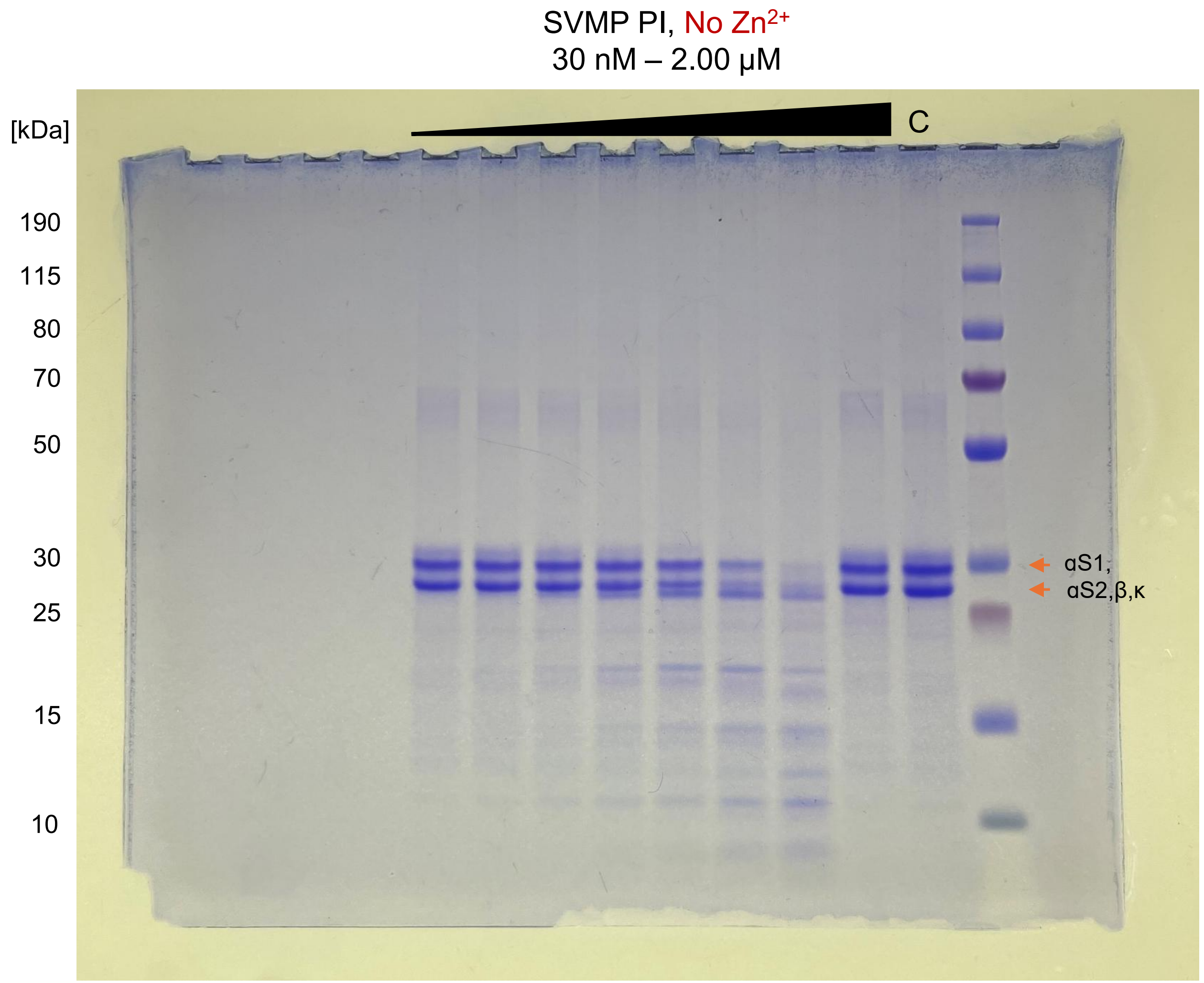

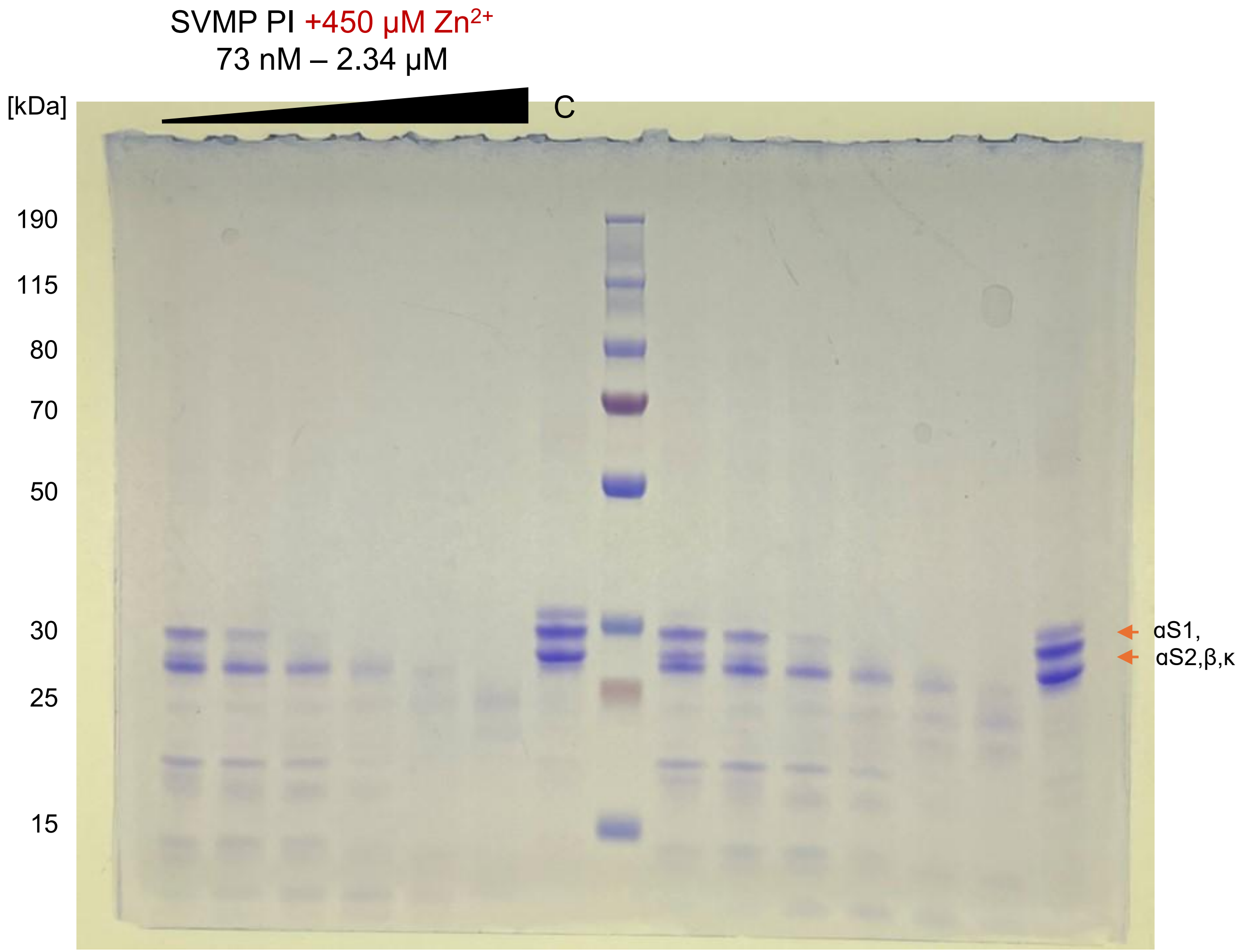

Supplement: Figure 3—figure supplement 3—source data 2. [file elife-109112-fig3-figsupp3-data2.zip › Figure 3 supplement 3 - source data 2/Figure 3 supplement 3 - source data 2.pdf]
