# Supplementary material for: Purified zymogens reveal mechanisms of snake venom metalloproteinase auto-activation
Source: eLife. 2026 Jun 10;15:RP109112. doi: 10.7554/eLife.109112 (PMC13252954; doi:10.7554/eLife.109112)

Figure S7b

Reducing and non-reducing SDS-PAGE of SEC peaks 2 and 3 of PII SVMP zymogen

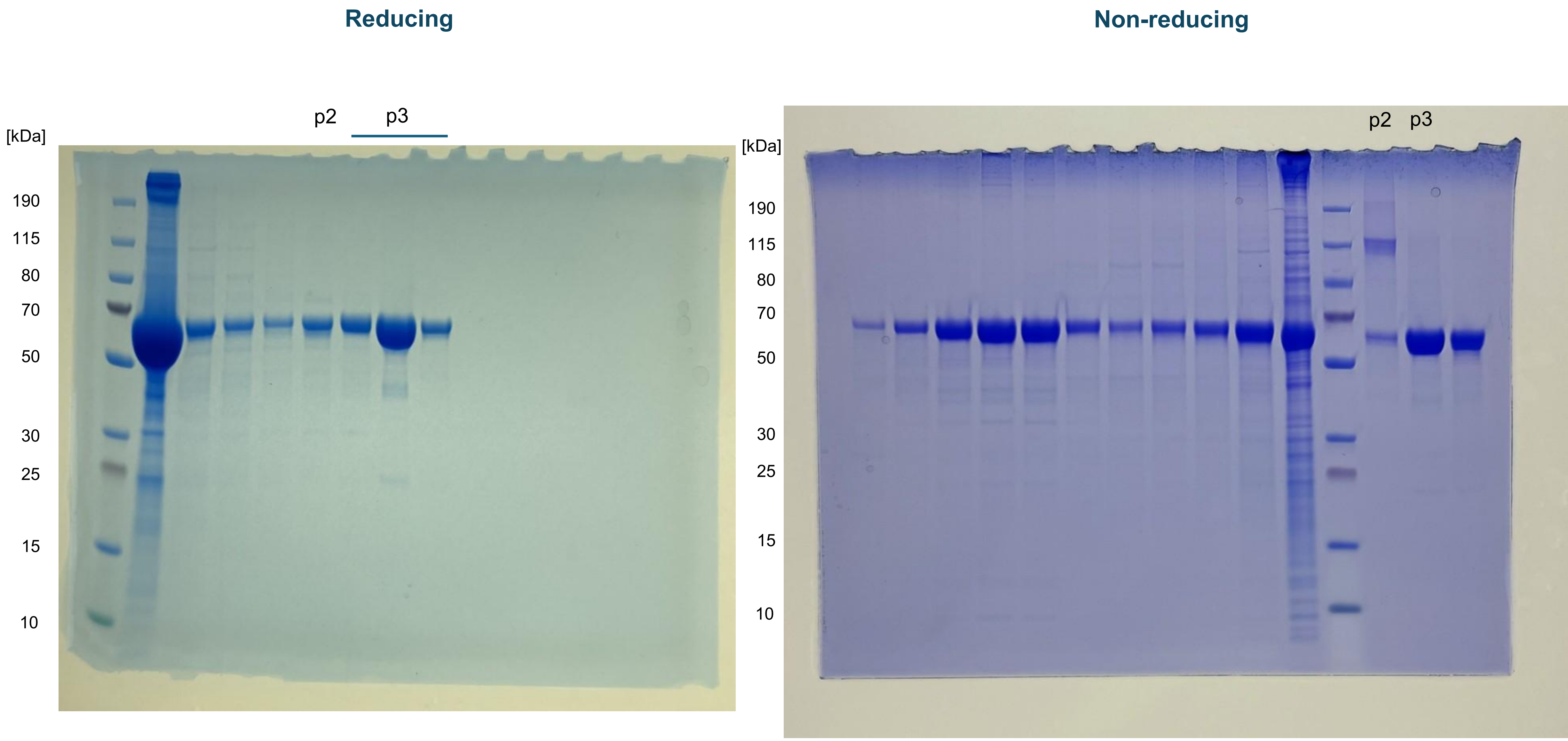

Figure S7c

Western blot analysis of sample before and after activation of PII SVMP.

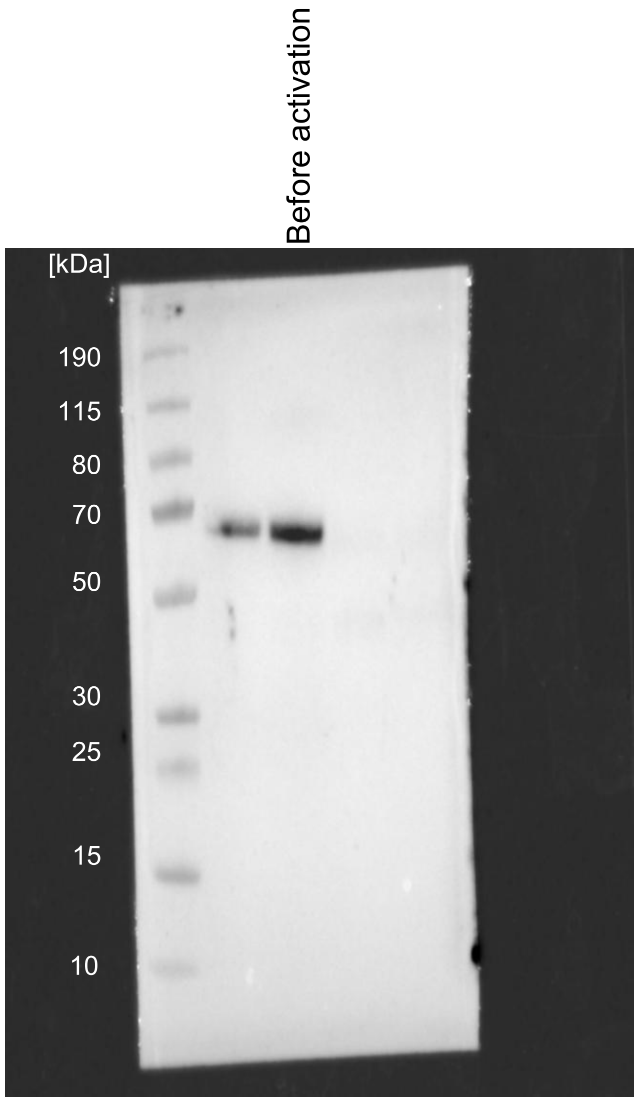

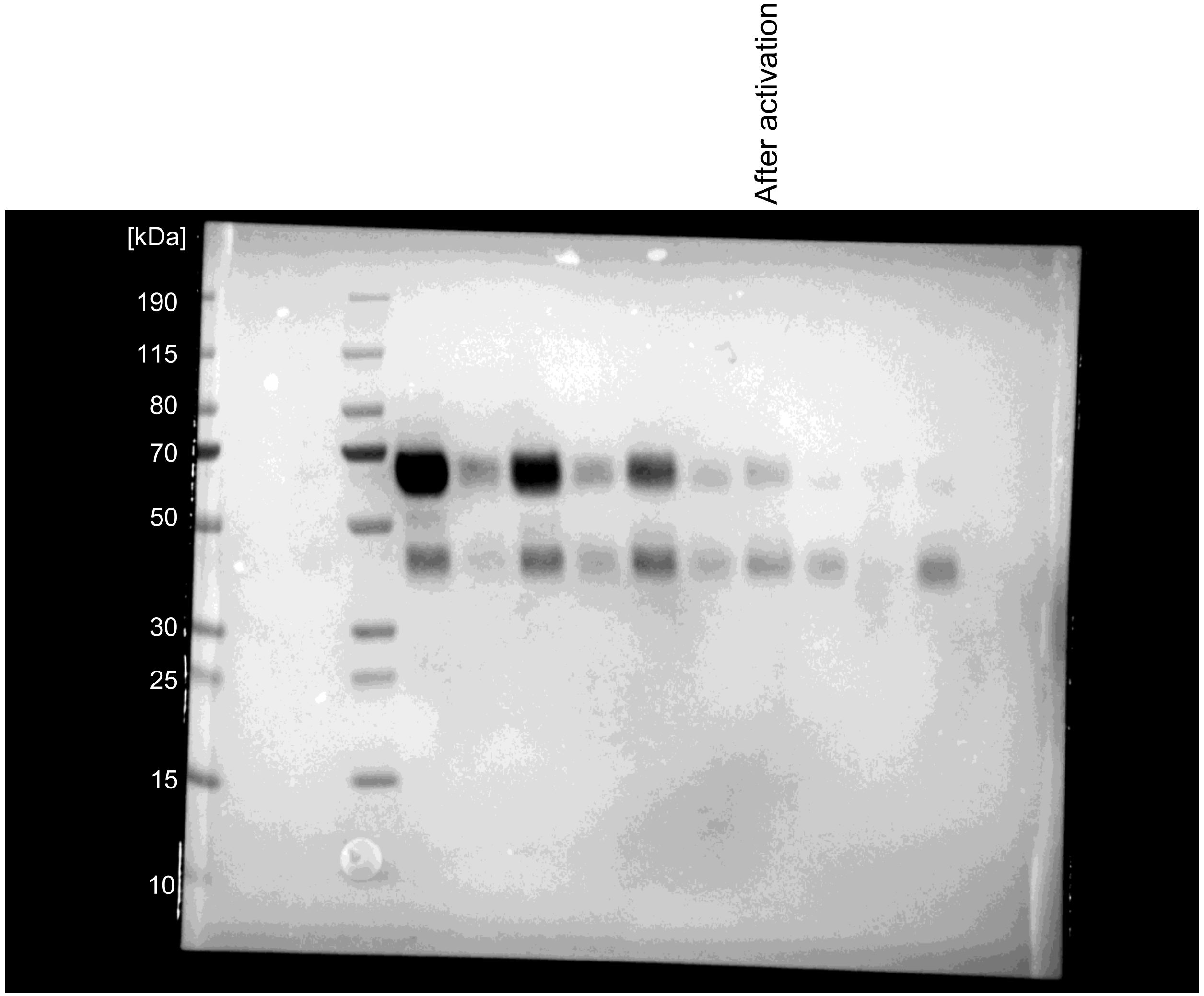

Supplement: Figure 3—figure supplement 4—source data 2. [file elife-109112-fig3-figsupp4-data2.zip › Figure 3 supplement 4 - source data 2/Figure 3 supplement 4 - source data 2.pdf]
