# Supplementary material for: Purified zymogens reveal mechanisms of snake venom metalloproteinase auto-activation
Source: eLife. 2026 Jun 10;15:RP109112. doi: 10.7554/eLife.109112 (PMC13252954; doi:10.7554/eLife.109112)

Figure S9b

PII SVMP zymogen – SDS-PAGE of SEC

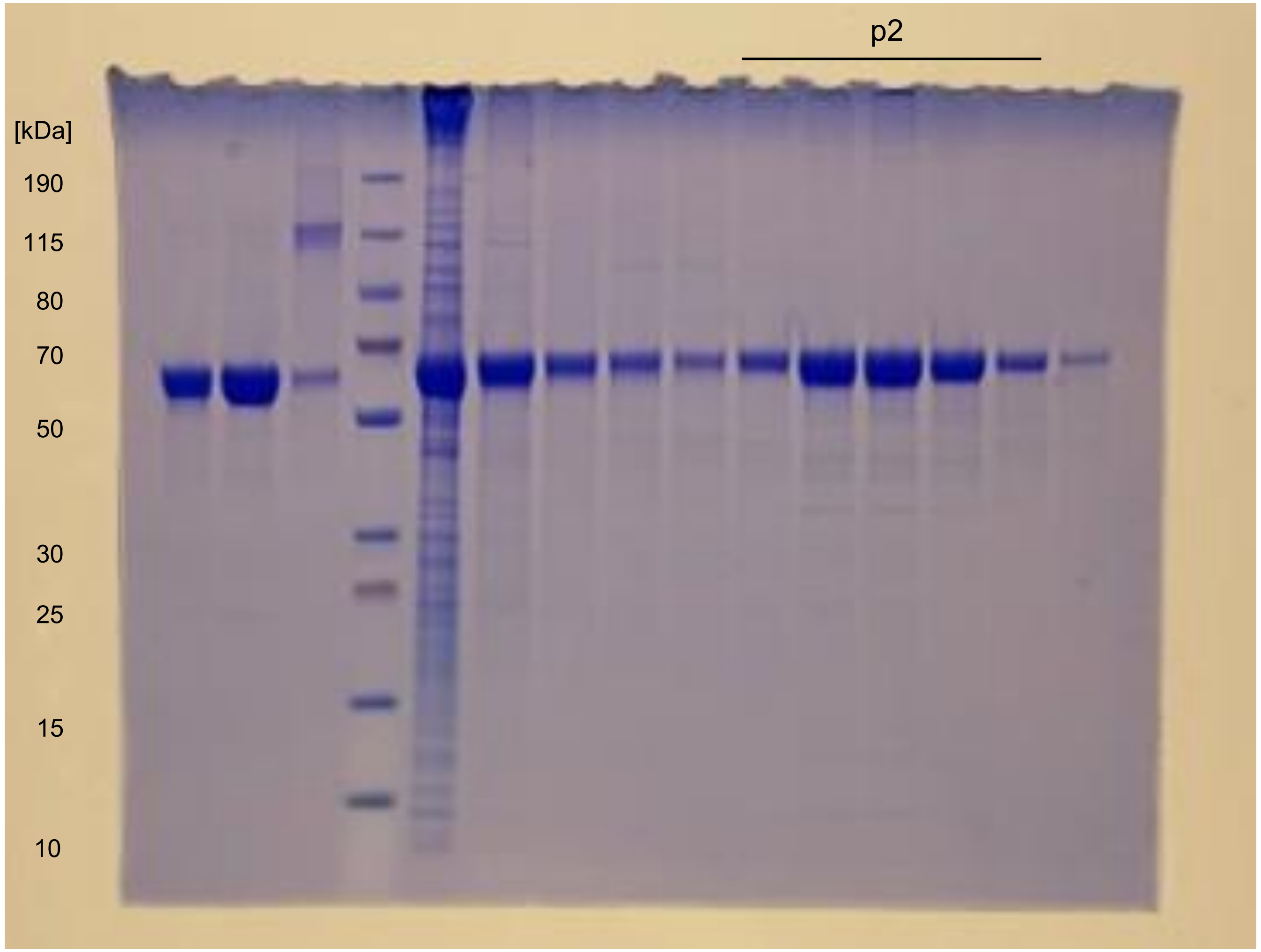

Figure S9c

PIII SVMP zymogen – SDS-PAGE of SEC

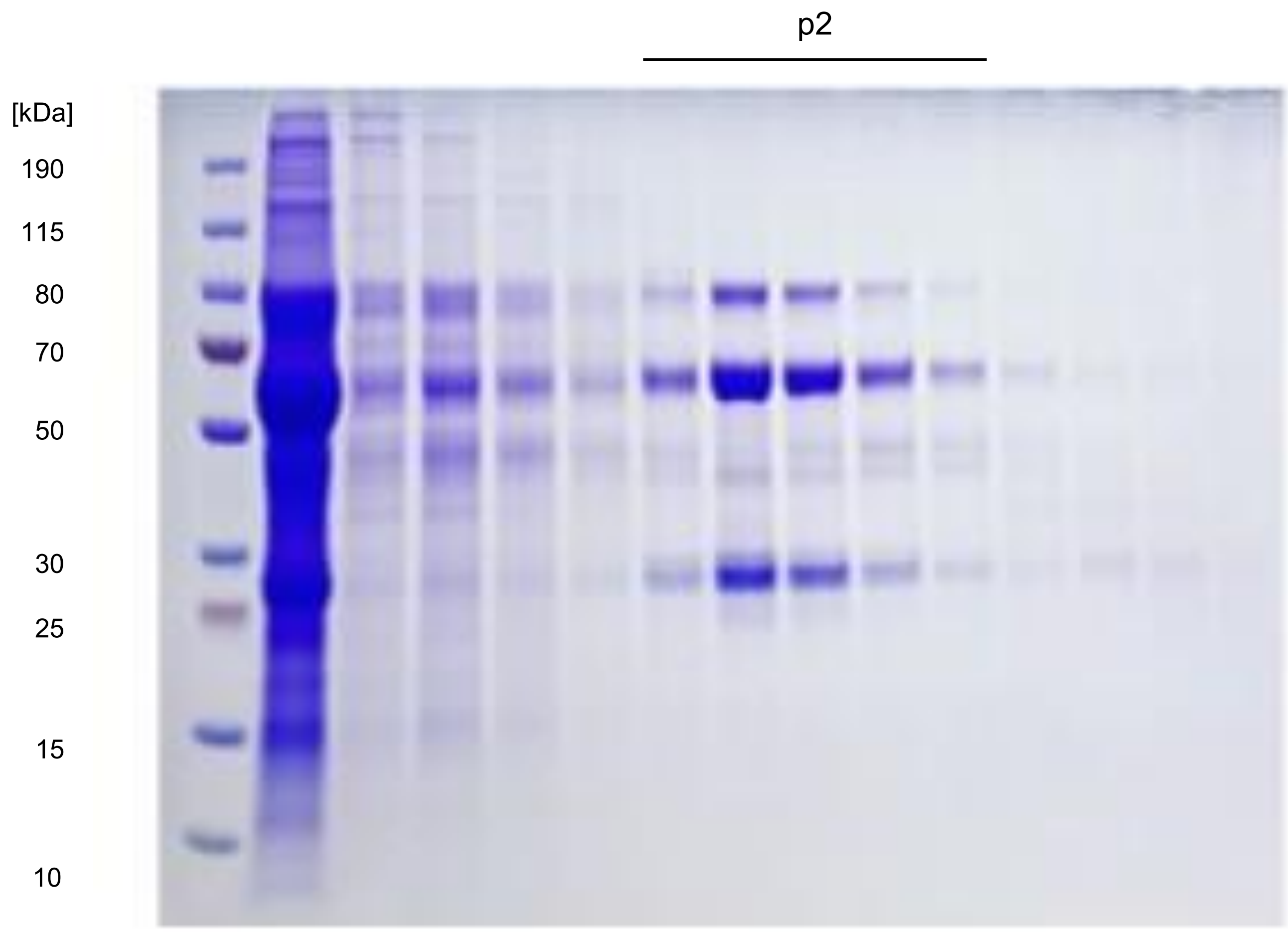

Figure S9d

Fibrinogen degradation assays in the presence of Zn2+ and increasing amounts of SVMPs PII, PII + hPDI (human PDI) and PII +s PDI (snake PDI)

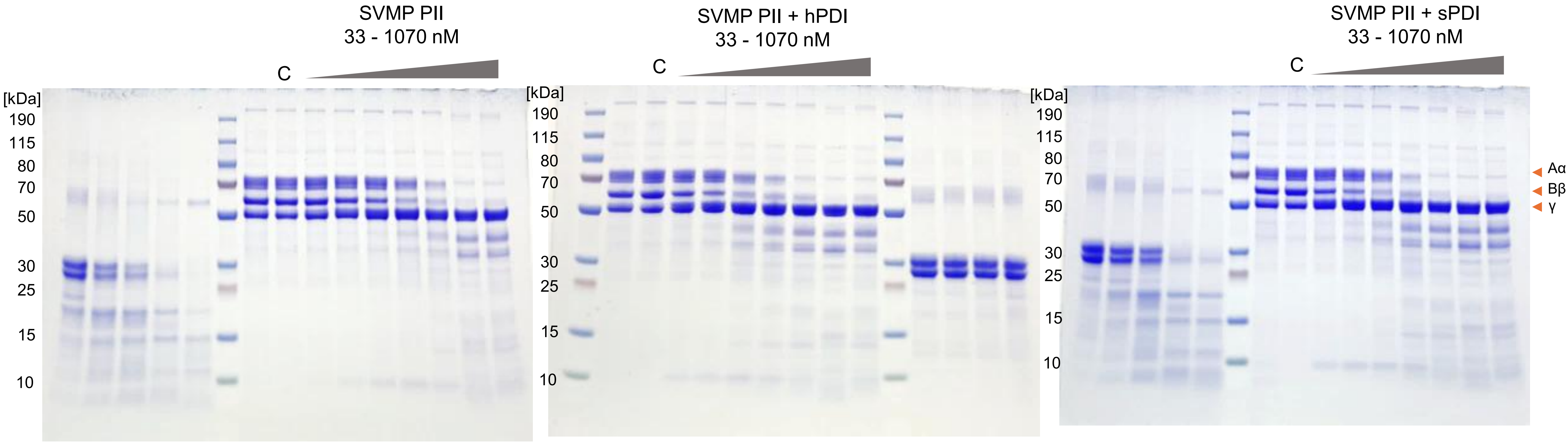

Figure S9e

Fibrinogen degradation assays in the presence of Zn2+ and increasing amounts of SVMPs PIII, PIII + hPDI and PIII + sPDI

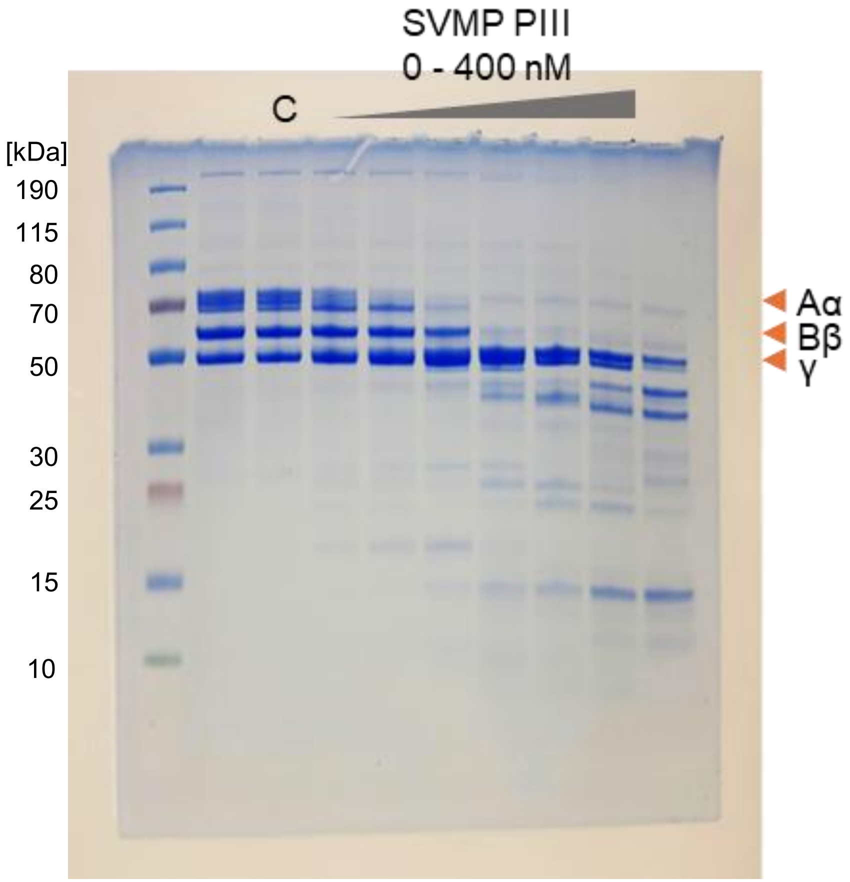

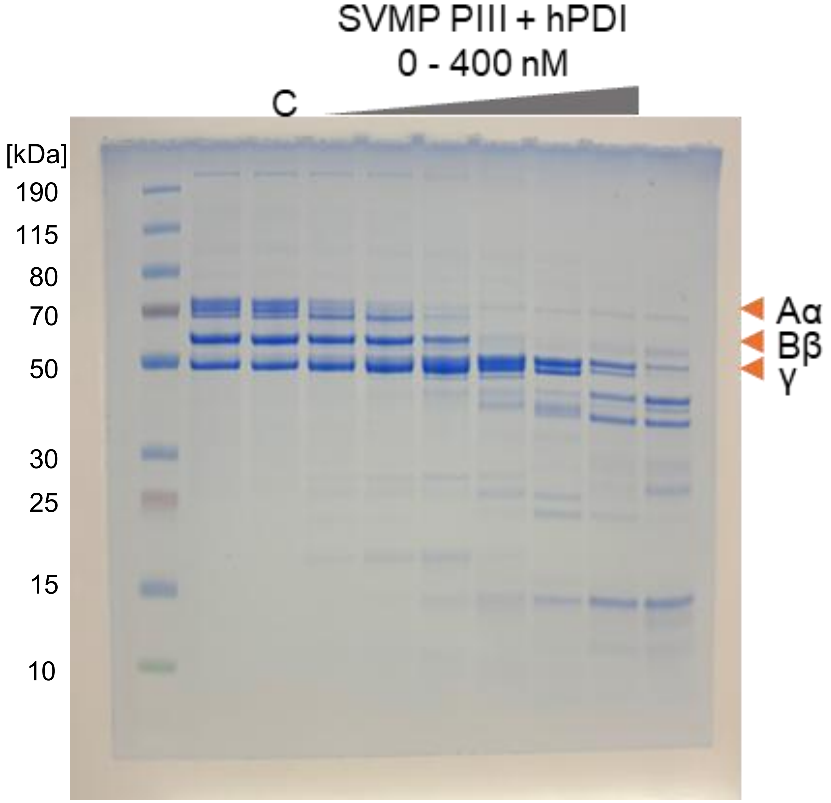

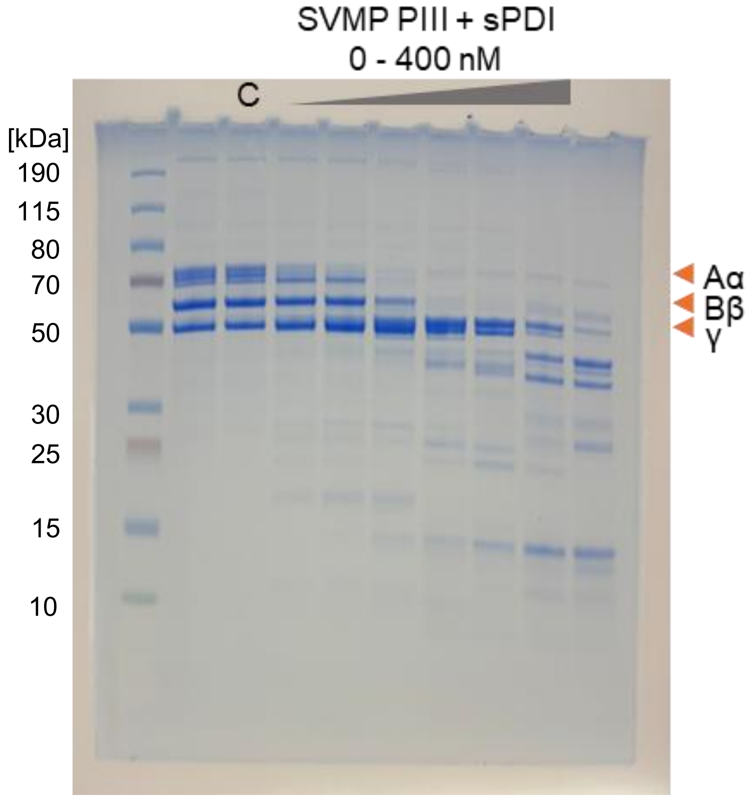

Supplement: Figure 3—figure supplement 6—source data 2. [file elife-109112-fig3-figsupp6-data2.zip › Figure 3 supplement 6 - source data 2/Figure 3 supplement 6 - source data 2.pdf]
