# Supplementary material for: Purified zymogens reveal mechanisms of snake venom metalloproteinase auto-activation
Source: eLife. 2026 Jun 10;15:RP109112. doi: 10.7554/eLife.109112 (PMC13252954; doi:10.7554/eLife.109112)

Figure 4a

SVMP PI – Casein degradation

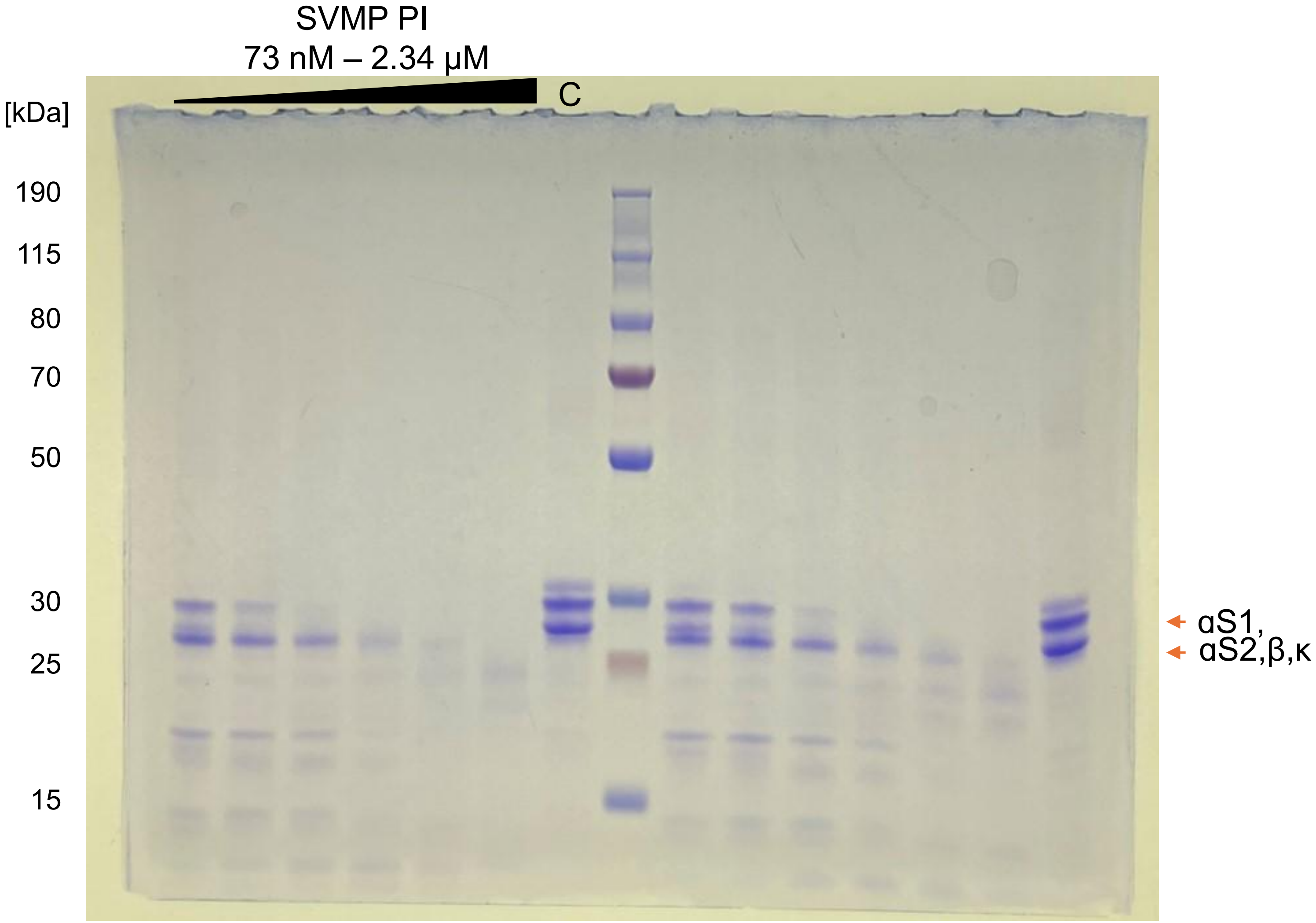

Figure 4b

SVMP PII – Casein degradation

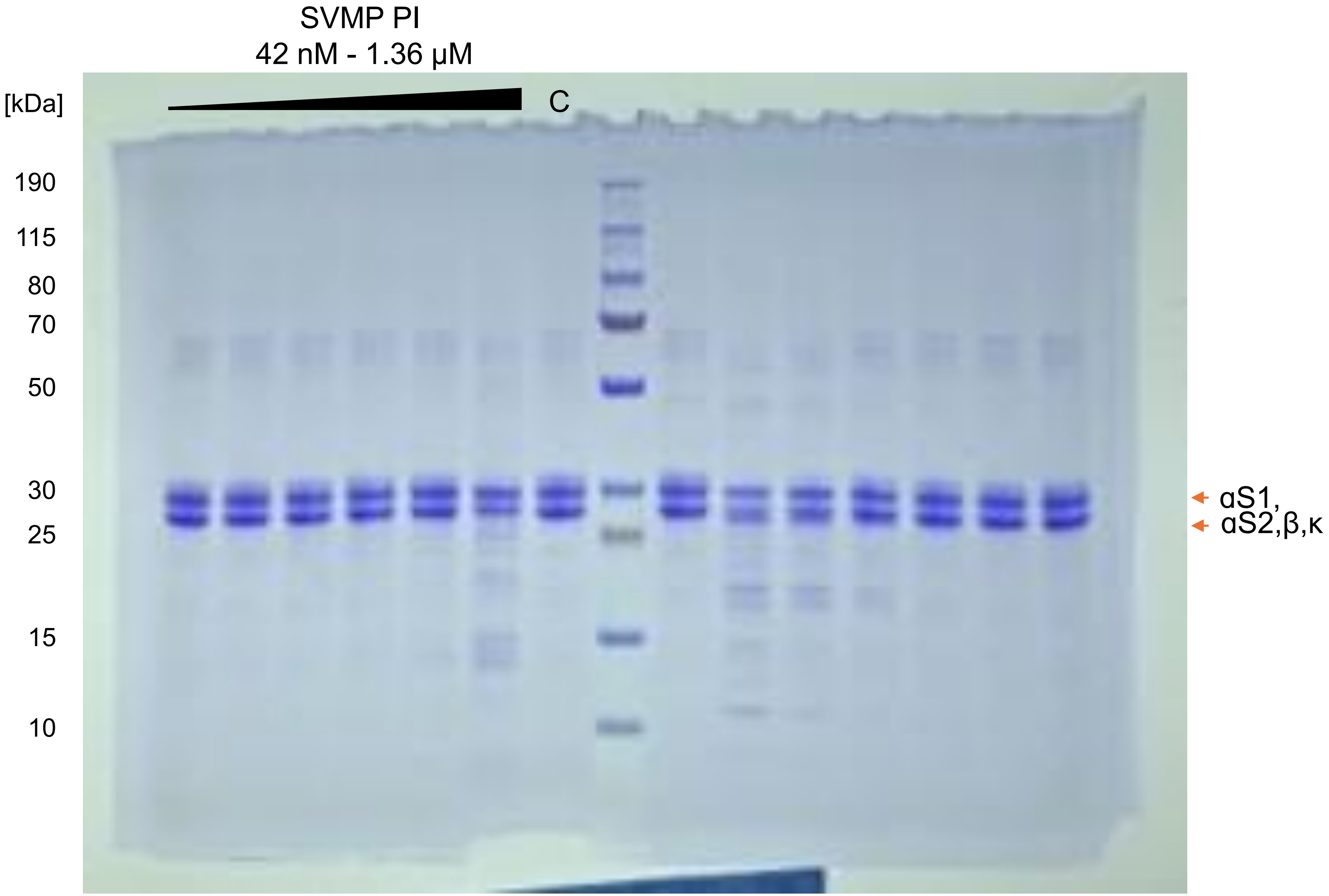

Figure 4c

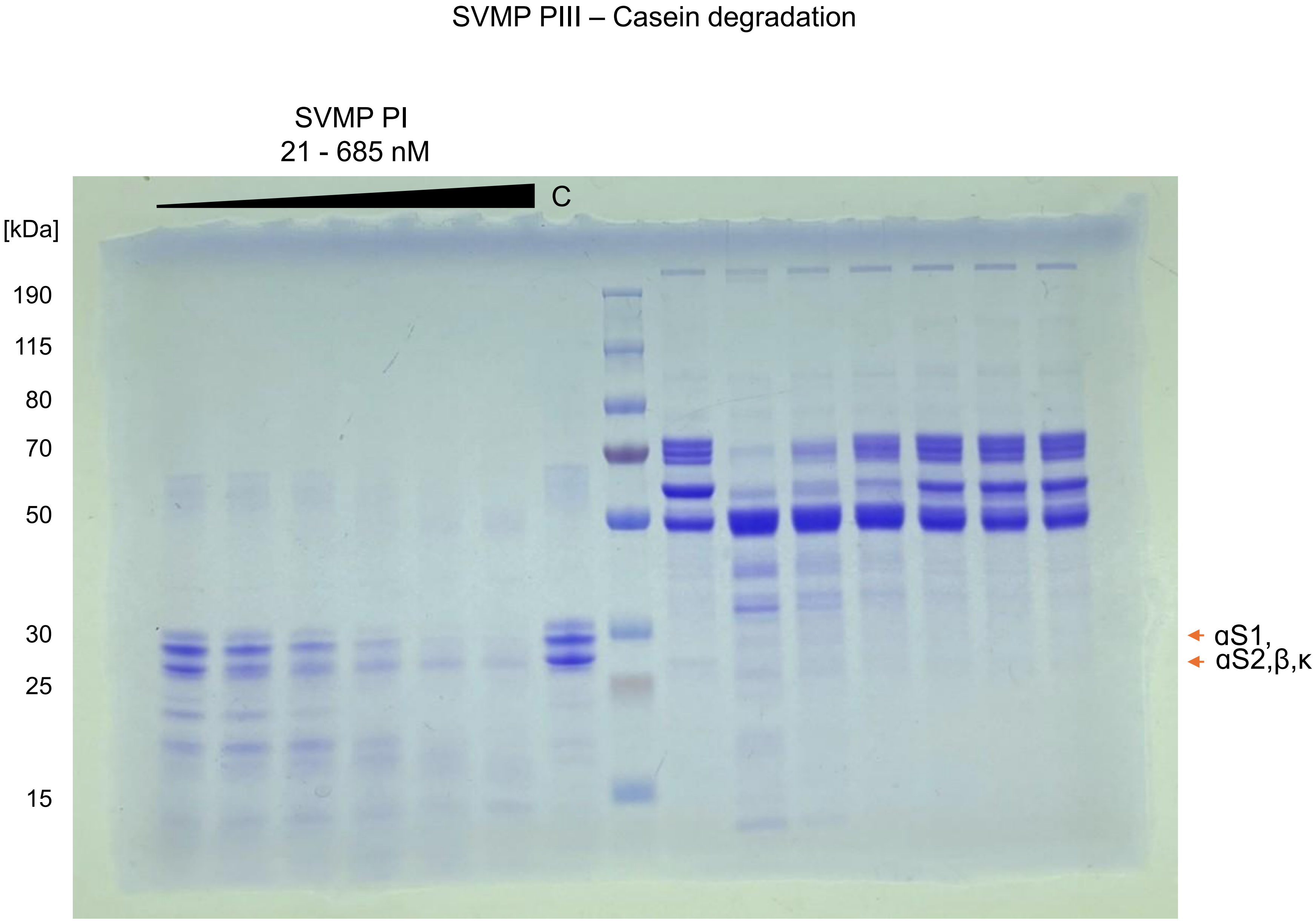

SVMP PIII – Casein degradation
SVMP PI
21 - 685 nM
C
[kDa]
190
115
80
70
50
30
25
15
αS1,
αS2,β,κ

Figure 4d

SVMP PI – Fibrinogen degradation

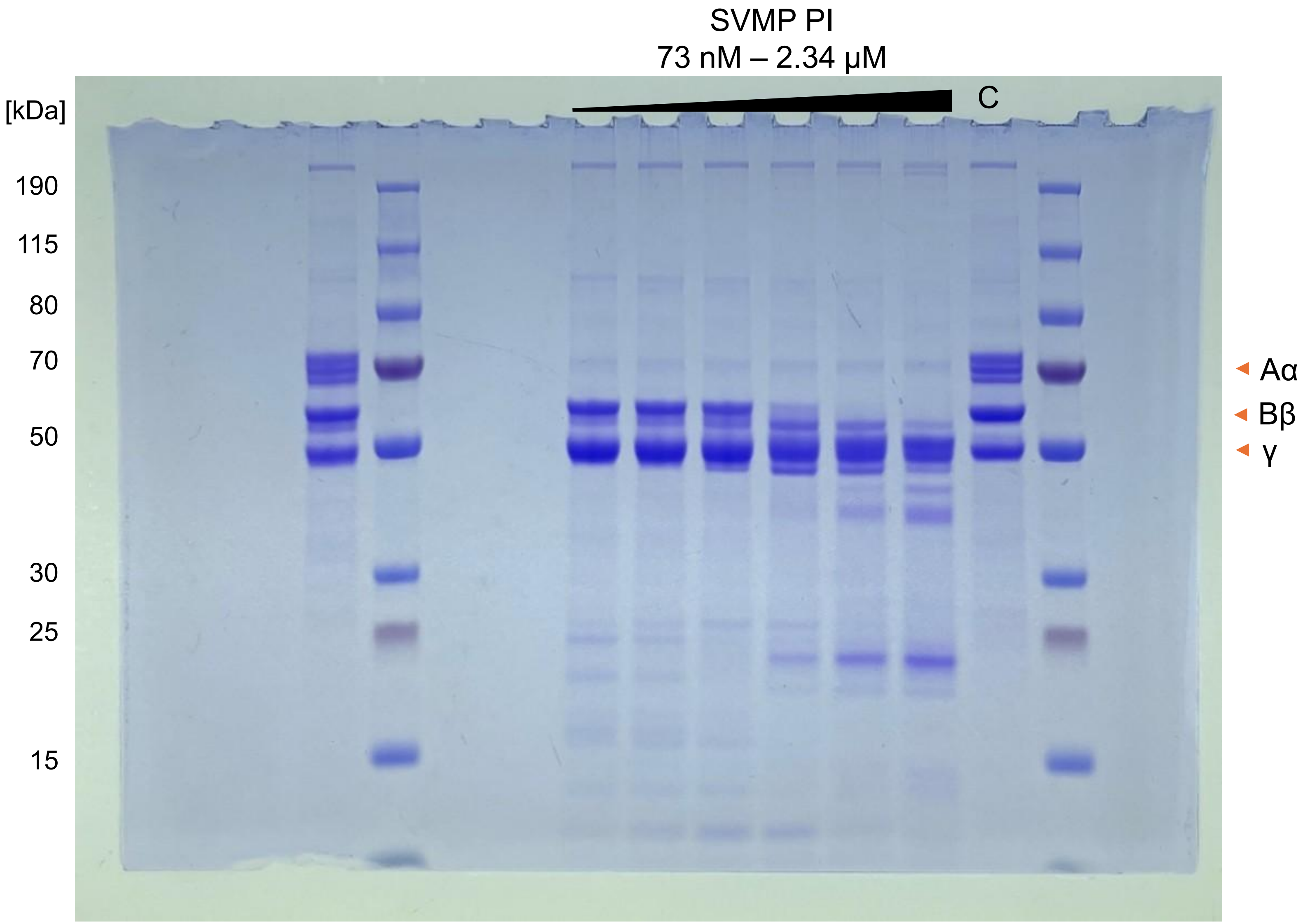

Figure 4e

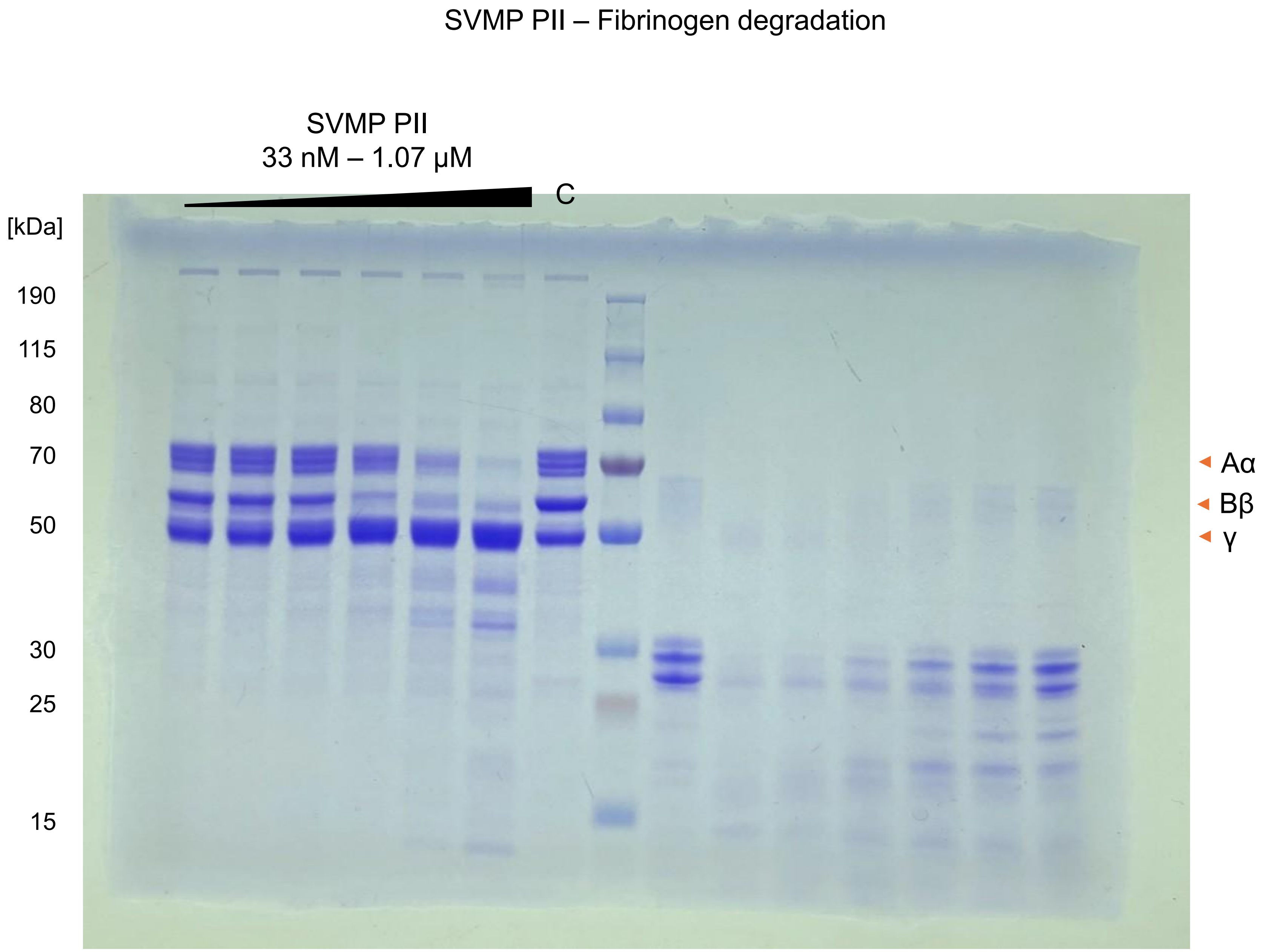
SVMP PII – Fibrinogen degradation
SVMP PII
33 nM – 1.07 µM
C
[kDa]
190
115
80
70
50
30
25
15
Aα
Bβ
γ

Figure 4f

SVMP PIII – Fibrinogen degradation

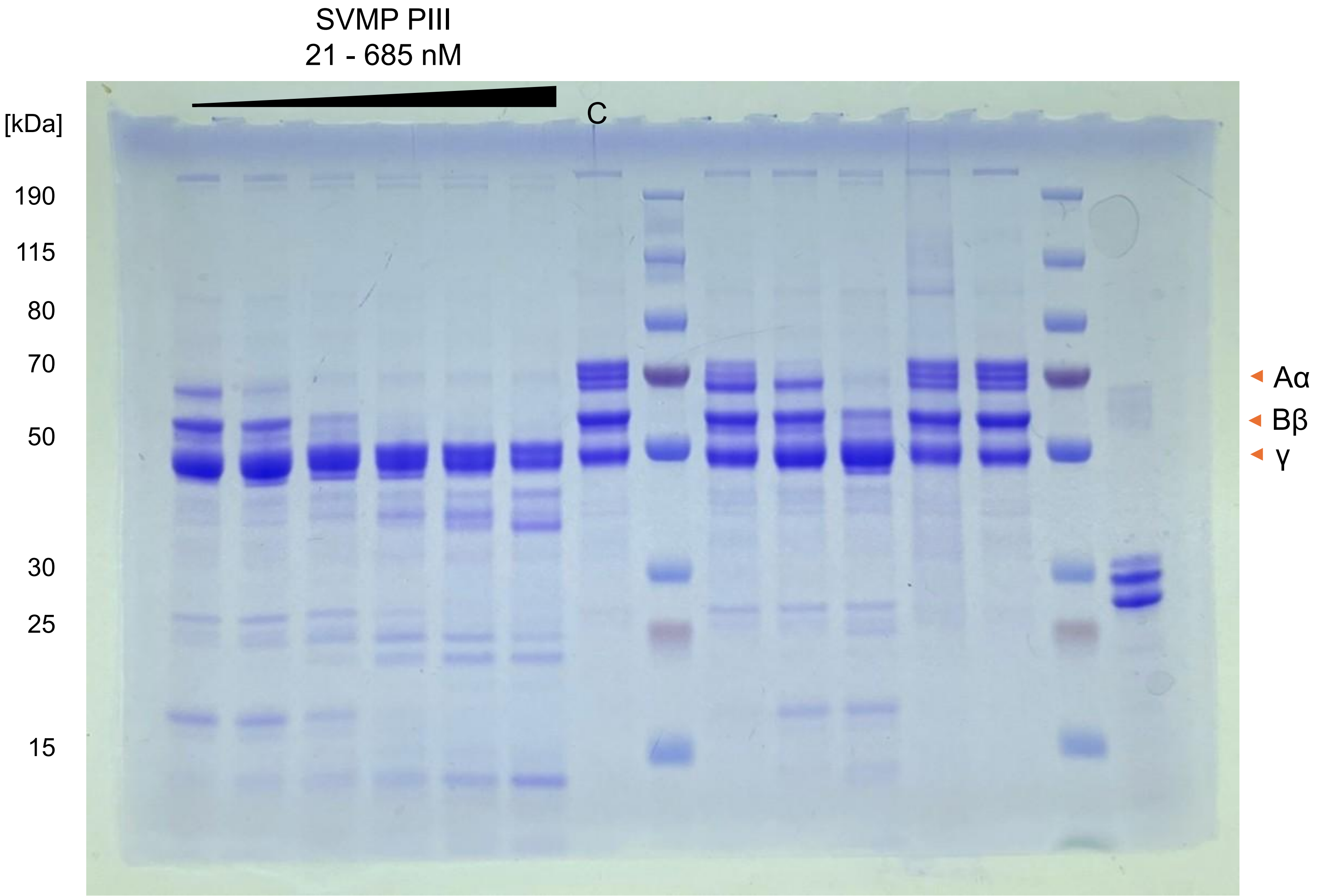

Supplement: Figure 4—source data 2. [file elife-109112-fig4-data2.zip › Figure 4 - source data 2.pdf]
