# Supplementary material for: Purified zymogens reveal mechanisms of snake venom metalloproteinase auto-activation
Source: eLife. 2026 Jun 10;15:RP109112. doi: 10.7554/eLife.109112 (PMC13252954; doi:10.7554/eLife.109112)
Supplement: Peer review history

# Editor report: eLife Assessment

**DOI:** 10.7554/eLife.109112.3.sa0

**Authors:** Koyeli Mapa

**Affiliations:** Department of Life Sciences, School of Natural Sciences, Shiv Nadar University India

**Keywords:** Solid, Useful

In this **useful** paper, the authors present a comprehensive method for the purification of recombinant Snake Venom Metalloproteinases (SVMPs) using the MultiBac expression system, explain the self-activation of the enzymes by Zn2+ incubation, and establish high-throughput screening (HTS) techniques. The authors addressed a key problem: producing a substantial amount of pure and enzymatically active SVMPs required for structural and functional studies. Altogether, this work builds a **solid** foundation for the large-scale production of active SVMPs for future biochemical and structural characterization as well as for drug discovery, albeit leaving certain caveats about the universal applicability of the described methodology for the production of any recombinant SVMPs.

# Referee report: Reviewer #1 (Public review)

**DOI:** 10.7554/eLife.109112.3.sa1

Summary:

The authors Hall et al. establish a purification method for snake venom metalloproteinases (SVMPs). By generating a generic approach to purify this divergent class of recombinant proteins, they enhance the field's accessibility to larger quantity SVMPs with confirmed activity and, for some, characterized kinetics. In some cases, the recombinant protein displayed comparable substrate specificity and substrate recognition compared to the native enzyme, providing convincing evidence of the authors' successful recombinant expression strategy. Beyond describing their route towards protein purification, they further provide evidence for self-activation upon Zn2+ incubation. They further provide initial insights on how to design high throughput screening (HTS) methods for drug discovery and outline future perspectives for the in-depth characterization of these enzyme classes to enable the development of novel biomedical applications.

Strengths:

The study is well presented and structured in a compelling way and the universal applicability of the approach is nicely presented.

The purification strategy results in highly pure protein products, well characterized by size exclusion chromatography, SDS page as well as confirmed by mass spectrometry analysis. Further, a significant portion of the manuscript focuses on enzyme activity, thereby validating function. Particularly convincing is the comparability between recombinant vs. native enzymes; this is successfully exemplified by insulin B digestion. By testing the fluorogenic substrate, the authors provide evidence that their production method of recombinant protein can open up possibilities in HTS. Since their purification method can be applied to three structurally variable SVMP classes, this demonstrates the robust nature of the approach.

Weakness

The product obtained from the purification protocol appears to be a heterogenous mixture of self-activated and intact protein species. The protocol would benefit from improved control over the self-activation process. The authors explain well why they cannot deplete Zn2+ in cell culture or increase the pH to prevent autoactivation during the current purification steps. However, this leads me to the suggestion, if the His tag could be exchanged to a different tag that is less pH sensitive and not dependent on divalent ions (Strep-Tactin XT?) to allow for removal of divalent ions and low pH during purification steps. Another suggestion would be if they could replace the endogenous protease cleavage site in their expression construct design to a TEV protease recognition site, for example, to have more control over activation of the recombinant proteins.

The graphic to explain the universal applicability of the approach, Figure S1, has some mistakes, like duplication of text, an arrow without a meaning and should be revised.

Overall, the authors successfully purified active SVMP proteins of all three structurally diverse classes in high quality and provided convincing evidence throughout the manuscript to support their claims. The described method will be of use for a broader community working with self-activating and cytotoxic proteases.

Comment on the revised version:

I find that the clarity and overall structure of the manuscript have improved. However, the weakness I previously highlighted has neither been addressed experimentally nor convincingly explained. Therefore, the assessment stayed unchanged from my side.

# Referee report: Reviewer #2 (Public review)

**DOI:** 10.7554/eLife.109112.3.sa2

Summary:

The aim of the study by Hall et al. was to establish a generic method for production of Snake Venom Metalloproteases (SVMPs). These have been difficult to purify in the mg quantities required for mechanistic biochemical and structural studies.

Strengths:

The authors have successfully applied the MultiBac system and describe with a high level of details, the downstream purification methods applied to purify the SVMP PI, PII and PIII. The paper carefully presents the non-successful approaches taken (such as expression of mature proteins, the use of protease inhibitors, prodomain segments and co-expression of disulfide-isomerases) before establishing the construct and expression conditions required. The authors finally convincingly describe various activity assays to demonstrate the activity of the purified enzymes in a variety of established SVMP assays.

Weaknesses:

Some experiments are difficult to perform with relevant controls (i.e. native SVMP from the venome), but authors have explained this and provided the best possible assessment.

Overall, the data presented demonstrates a very credible path for production of active SVMP for further downstream characterization. The generality of the approach to all SVMP from different snakes remains to be demonstrated by the community, but if generally applicable, the method will enable numerous studies with the aim of either utilizing SVMPS as therapeutic agents or to enable generation of specific anti-venom reagents such as antibodies or small molecule inhibitors.

Comment on the revised version:

I think the manuscript has benefited from the review and the revised version provides more clarity, is more concise and reads significantly better with the preliminary data/experiments moved to the supplements. My overall assessment of the manuscript remains unchanged.

# Author comment: Author response

**DOI:** 10.7554/eLife.109112.3.sa3

**Authors:** Sophie Hall, Iara Aime Cardoso, Mark C Wilkinson, Maria Molina Carretero, Srikanth Lingappa, Bronwyn Rand, Dakang Shen, Johara Boldrini-França, Richard Stenner, Stefanie Kate Menzies, Georgia Balchin, Konrad Kamil Hus, Renaud Vincentelli, Andrew Mumford, Alastair Poole, Nicholas R Casewell, Imre Berger, Christiane Schaffitzel

**Affiliations:** University of Bristol Bristol United Kingdom; University of Bristol Bristol United Kingdom; Liverpool School of Tropical Medicine Liverpool United Kingdom; University of Bristol Bristol United Kingdom; University of Bristol Bristol United Kingdom; University of Bristol Bristol United Kingdom; University of Bristol Bristol United Kingdom; University of Bristol Bristol United Kingdom; University of Bristol Bristol United Kingdom; Liverpool School of Tropical Medicine Liverpool United Kingdom; University of Bristol Bristol United Kingdom; University of Bristol Bristol United Kingdom; https://ror.org/035xkbk20 Aix-Marseille Université Marseille France; University of Bristol Bristol United Kingdom; University of Bristol Bristol United Kingdom; Liverpool School of Tropical Medicine Liverpool United Kingdom; University of Bristol Bristol United Kingdom; University of Bristol Bristol United Kingdom

The following is the authors’ response to the original reviews.

**Reviewer #1 (Public review):**

Summary:

The authors Hall et al. establish a purification method for snake venom metalloproteinases (SVMPs). By generating a generic approach to purify this divergent class of recombinant proteins, they enhance the field's accessibility to larger quantities of SVMPs with confirmed activity and, for some, characterized kinetics. In some cases, the recombinant protein displayed comparable substrate specificity and substrate recognition compared to the native enzyme, providing convincing evidence of the authors' successful recombinant expression strategy. Beyond describing their route towards protein purification, they further provide evidence for self-activation upon Zn2+ incubation. They further provide insights on how to design high-throughput screening (HTS) methods for drug discovery and outline future perspectives for the in-depth characterization of these enzyme classes to enable the development of novel biomedical applications.

Strengths:

The study is well-presented and structured in a compelling way. The purification strategy results in highly pure protein products, well characterized by size exclusion chromatography, SDS page as well as confirmed by mass spectrometry analysis. Further, a significant portion of the manuscript focuses on enzyme activity, thereby validating function. Particularly convincing is the comparability between recombinant vs. native enzymes; this is successfully exemplified by insulin B digestion. By testing the fluorogenic substrate, the authors provide evidence that their production method of recombinant protein can open up possibilities in HTS. Since their purification method can be applied to three structurally variable SVMP classes, this demonstrates the robust nature of the approach.

We thank the reviewer for their positive assessment of our work.

Weaknesses:

The universal applicability of the approach could be emphasized more clearly. The potential for this generic protocol for recombinant SVMP zymogen production to be adapted to other SVMPs is somewhat obscured by the detailed optimization steps. A general schematic overview would strengthen the manuscript, presented as a final model, to illustrate how this strategy can be extended to other targets with similar features. Such a schematic might, for example, outline the propeptide fusion design, including its tags, relevant optimizations during expression, lysis, purification (e.g., strategies for metal ion removal and maintenance of protease inactivity), as well as the controllable auto-activation.

In the revised version of the manuscript, we moved the detailed description of the optimisation of SVMP expression, including mature SVMP expression, Marimastat addition, active site mutations and fusion of propeptides, into the supplement as supplementary text. We hope this improves the clarity and flow. As suggested, we now include a new figure outlining the SVMP production strategy and optimisation steps in the revised manuscript (new Figure S1).

The product obtained from the purification protocol appears to be a heterogeneous mixture of selfactivated and intact protein species. The protocol would benefit from improved control over the selfactivation process. The Methods section does not indicate whether residual metal ions were attempted to be removed during the purification, which could influence premature activation.

We agree that improved control of self-activation would be desirable. However, there is an issue: Previous studies reported that (1) SVMP zymogens are processed within secretory cells of the venom gland (Portes-Junior et al., 2014), and (2) mature SVMPs accumulate in secretory vesicles during venom production (Carneiro et al., 2002). Accordingly, preventing the auto-processing of SVMP zymogens is difficult to achieve because this would require Zn^2+^ depletion within the insect cells during production which would result in cytotoxicity. We have included this information in the updated Discussion section of the revised manuscript.

Additionally, it has not been discussed whether the shift to pH 8 in the purification process is necessary from the initial steps onwards, given that a lower pH would be expected to maintain enzyme latency.

The shift to pH 8 is required for the affinity purification of the SVMP zymogens from the medium, involving the poly-histidine-tag and immobilized metal affinity chromatography (IMAC). At lower pH, the histidines would become protonated, preventing binding of the His-tag to the column. Thus, with the His-tag the shift to pH 7.5 or pH 8 is necessary.

The characterization of PIII activity using the fluorogenic peptide effectively links the project to its broader implications for drug design. However, the absence of comparable solutions for PI and PII classes limits the overall scope and impact of the finding.

We agree that such assays would be extremely useful. However, the development of fluorescence based high-throughput assays to test for PI and PII SVMP activity is beyond the scope of this study. Here, our overarching objective is to report a broadly applicable production method for PI, PII and PIII SVMPs.

Overall, the authors successfully purified active SVMP proteins of all three structurally diverse classes in high quality and provided convincing evidence throughout the manuscript to support their claims. The described method will be of use for a broader community working with self-activating and cytotoxic proteases.

Thank you.

**Reviewer #2 (Public review):**

Summary:

The aim of the study by Hall et al. was to establish a generic method for the production of Snake Venom Metalloproteases (SVMPs). These have been difficult to purify in the mg quantities required for mechanistic, biochemical, and structural studies.

Strengths:

The authors have successfully applied the MultiBac system and describe with a high level of detail the downstream purification methods applied to purify the SVMP PI, PII, and PIII. The paper carefully presents the non-successful approaches taken (such as expression of mature proteins, the use of protease inhibitors, prodomain segments, and co-expression of disulfide-isomerases) before establishing the construct and expression conditions required. The authors finally convincingly describe various activity assays to demonstrate the activity of the purified enzymes in a variety of established SVMP assays.

We thank the reviewer for their positive assessment of our work.

Weaknesses:

The manuscript suffers from a lack of bottoming out and stringent scientific procedures in the methodology and the characterization of the generated enzymes.

As an example, a further characterization of the generated protein fragments in Figure 3 by intact mass spectroscopy would have aided in accurate mass determination rather than relying on SEC elution volumes against a standard. Protein shape and charge can affect migration in SEC.

We agree that intact MS would be useful to determine the mass of the produced SVMPs. In this manuscript, we performed SEC as a purification step, removing aggregates. Furthermore, SEC allowed determining if the SVMPs form monomers or dimers. MS characterisation of intact SVMPs (and their PTMs) is not trivial and beyond the scope of this manuscript (see below).

Also, the analysis of N-linked glycosylation demonstrates some reactivity of PIII to PNGase F, but fails to conclude whether one or more sites are occupied, or whether other types of glycosylation is present. Again, intact mass experiments would have resolved such issues.

We concur that glycosylation of SVMPs is an important question. However, analysing the glycosylation of the SVMPs is beyond the scope of this manuscript; it is actually a project on its own: Intact MS can indeed provide information on glycosylation but is not very precise. Unambiguous assignment of the number and occupancy of glycosylation sites is more challenging, especially for large, glycosylated proteins such as our PIII SVMP zymogen. In practice, confident mapping of glycosylation sites would require peptide-level mass spectrometry following enzymatic digestion (Trypsin and Multi-Enzymatic Limited Digestion, ideally). Sample preparation, method optimization, MS acquisition, and data analysis together would require a significant investment. Moreover, we do not have access to the native PIII SVMP from *Echis carinatus sochureki* venom - this is the main point of our manuscript: we describe a protocol to produce SVMPs which could not be purified from venom. Therefore, a comparison of the glycosylation of the recombinant SVMP and the native SVMP cannot be performed unfortunately (see below).

The activity assays in Figure 4 are not performed consistently with kinetic assays and degradation assays performed for some, but not all, enzymes, and there is no Echis ocellatus comparison in Figure 4h.

This is correct. The suggested control experiment is not possible for the PII SVMP and PIII SVMP because we cannot purify the native PII and PIII SVMPs from *Echis* venom. We have highlighted this information in the revised manuscript in the insulin B degradation section.

Overall, whilst not affecting the main conclusion, this leaves the reader with an impression of preliminary data being presented. For consistency, application of the same assays to all enzymes (high-grade purified) would have provided the reader with a fuller picture.

In the revised manuscript, we included new data showing the requested characterisations of all three SVMPs.

We have included the respective assays in Figure 5 and Supplementary Figure S11. In the original manuscript, we had omitted these assays as the data show no enzymatic activity in the respective assays. Specifically, we show that (1) PII does not cause insulin B degradation (Fig. S11b), (2) that the PI and PII SVMPs do not degrade the fluorogenic peptide which is prototypic for PIII SVMPs and MMPs (Fig. S11a), (3) PI and PIII do not cause platelet aggregation because they lack the entire disintegrin domain (PI) or the RGD motif (PIII) (Fig. 5a), and (4) that the PI and PII SVMPs, like the PIII SVMP, are not pro-coagulant and do not cause blood clotting (Fig. 5d,5e and Fig. S11c). We also included this new information in the main text of our revised manuscript.

Overall, the data presented demonstrates a very credible path for the production of active SVMP for further downstream characterization. The generality of the approach to all SVMP from different snakes remains to be demonstrated by the community, but if generally applicable, the method will enable numerous studies with the aim of either utilizing SVMPS as therapeutic agents or to enable the generation of specific anti-venom reagents, such as antibodies or small molecule inhibitors.

Thank you.

**Reviewer #3 (Public review):**

Summary:

The presented study describes the long journey towards the expression of members' SVMP toxins from snake venom, which are toxins of major importance in a snakebite scenario. As in the past, their functional analysis relied on challenging isolation; the toxins' heterologous expression offers a potential solution to some major obstacles hindering a better understanding of toxin pathophysiology. Through a series of laborious and elegantly crafted experiments, including the reporting of various failed attempts, the authors establish the expression of all three SVMP subtypes and prove their activity in bioassays. The expression is carried out as naturally occurring zymogens that autocleave upon exposure to zinc, which is a novel modus operandi for yielding fusion proteins and sheds also some new light on the potential mechanism that snakes use to activate enzymatic toxins from zymogenic preforms.

Strengths:

The manuscript draws from an extensive portfolio of well-reasoned and hypothesis-driven experiments that lead to a stepwise solution. The wetlands data generated is outstanding, although not all experiments along this rocky road to victory were successful. A major strength of the paper is that, translationally speaking, it opens up novel routes for biodiscovery since a first reliable platform for expression of an understudied, yet potent toxin class is established. The discovered strategy to pursue expression as zymogens could see broad application in venom biotechnology, where several toxin types are pending successful expression. The work further provides better insights into how snake toxins are processed.

We thank the reviewer for their positive assessment of our work.

Weaknesses:

The manuscript contains several chapters reporting failed experiments, which makes it difficult to follow in places.

Based on a similar comment of Reviewer 1, we now moved the ‘failed’ experiments reporting on SVMP expression optimisation to the supplement as new supplementary text. We hope that the revisions have improved the clarity and overall readability of our manuscript.

The reporting of experimental details, especially sample sizes and replicates, could be optimised.

The number of replicates has now been added to the figure legends in the revised manuscript. Detailed experimental information is found in the revised Methods part.

At the time of writing, it remains unclear whether the glycosilations detected at a pIII SVMP could have an impact on the bioactivities measured, which is a major aspect, and future follow-ups should clarify this.

A detailed analysis of glycosylation of the PIII SVMP is beyond the scope of our manuscript (see above, response to Reviewer 2). Our manuscript describes a generic protocol to produce active SVMPs. Importantly, we cannot purify the native PIII SVMP from *Echis carinatus sochureki* venom. Therefore, it is not possible to compare our PIII SVMP with the native PIII SVMP.

We agree that this is an important question, and we will aim in the future to perform such a comparison of a different insect cell-produced PIII with a native PIII SVMP that can be readily purified from venom.

Finally, the work, albeit of critical importance, would benefit from a more down-to-earth evaluation of its findings, as still various persistent obstacles that need to be overcome.

We consider cytotoxicity to be the principal bottleneck in SVMP production. In this study, we present a strategy to overcome this bottleneck.

Major comments to the manuscript:

(1) Lines 148-149: "indicating that expressing inactivated SVMPs could be a viable, although inefficient, approach". I think this text serves a good purpose to express some thoughts on the nature of how the current draft is set up. It is quite established that various proteases cause extreme viability losses to their expression host (whether due to toxicity, but surely also because of metabolic burden), which is why their expression as inactive fusion proteins is the default strategy in all cases I have thus far seen. I believe that, especially in venom studies, this is of importance given the increased toxicity often targeting cellular integrity, and especially here, because Echis are known to feed on arthropods at younger life history stages, making it very likely that some venom components are especially active against insects and other invertebrates. With that in mind, I would argue that exploring their production in inactive form is the obvious strategy one would come up with and not really the conclusion of a series of (well-conducted and scientifically sound!) experiments. For me, the insight of inactive expression is largely confirmatory of what is established, unless I miss something in the authors' rationale. If yes, it would be important to clarify that in the online version.

We agree that producing zymogens represents a straightforward strategy and now, in hindsight, would have wished we had tested this first thing, it would have saved us and apparently many others significant effort. However, realising this, and implementing this approach took us considerable time and insight as we described in this manuscript. The alternative strategies we describe in the manuscript, in particular the use of inhibitors and active-site mutation, have been successfully applied for recombinant production of diverse enzymes before, including enzymes that are toxic to host cells.

We have revised the manuscript as requested and moved the optimisation of SVMP expression to the Supplement. We hope this improved the clarity, overall readability of the text and thus addressed the reviewer’s comment.

(2) Line 173: Here, Alphafold 3 was used, whereas in previous sections (e.g., line 153, line 210), it was Alphafold 2. I suggest using one release across the manuscript.

Thank you for bringing this to our attention. In the revised version of the manuscript, we clarified that all models were generated using AlphaFold 3.

(3) Line 252-254: I fully agree, the PIII SVMP is glycosylated. Glycosylation is an important mediator of snake venom activity, and several works have described their importance in the field. This raises the question, which glycosylations have been introduced here in the SVMP, and to verify that these are glycosylations that belong to those found in snakes. This is important as insects facilitate thousands of N- and O- O-glycosylations to modulate the activity of their proteome, of which many are specific to insects. If some of these were integrated into the SVMP, this could have an impact on downstream produced bioassays and also antigenicity (the surface would be somewhat different from natural toxins, causing different selection).

We agree that glycosylation is important and warrants a follow-up in the future.

However, most publications we found reported that de-glycosylation has a negative effect on stability and solubility of SVMPs, which is expected to have a knock-on effect on toxin activity (e.g. AndradeSilva et al., 2025; DOI: 10.1021/acs.jproteome.5c00249). It will be difficult to separate the two effects from each other. We found only a few examples where SVMP glycosylation (sialylation and Nglycosylation) modulated proteolytic and haemorrhagic functions, including interaction with substrates such as e.g. fibrinogen (Schluga et al., 2024; https://doi.org/10.3390/toxins16110486; Chen et al., 2008; 10.1111/j.1742-4658.2008.06540.x; Nikai et al., 2000; DOI: 10.1006/abbi.2000.1795. PMID: 10871038). In our manuscript, we show that our PIII SVMP is very cytotoxic and highly active in casein, fibrinogen and ESO10 degradation assays, with a K_M_ and k_cat_/K_M_ comparing favourably with other SVMPs and MMPs. We are not aware of a specific substrate for this particular PIII SVMP that depends on a distinct glycosylation pattern. Recombinant production of such SVMPs with specific glycosylation pattern requirement would be a challenge in all commonly used expression systems (yeast, plant, insect cells and mammalian cells). In fact, insect cell expression systems could be advantageous in this respect because the Sf21 and High Five (Hi5) lepidopteran cell lines we utilised are well-characterized for their ability to perform posttranslational modifications on complex secreted proteins:

(1) N-Glycan conservation: Both Sf21 and Hi5 cells typically produce N-glycans that are trimmed to a core 'paucimannose' structure (Man3GlcNAc2), often with an alpha1,6-fucosylation. While snakes can produce more complex, sialylated N-glycans, glycomic studies of native venoms (e.g., *Bothrops* venom) have demonstrated that high-mannose and paucimannose structures are also prevalent in native SVMPs. Therefore, the recombinant glycoforms produced in our system are not 'unnatural' in the snake venom context but rather represent a subset of the native glycan microheterogeneity.

(2) Occupancy vs structure: The critical function of glycosylation in PIII SVMPs is thought to be often structural, facilitating correct folding and protecting the large metalloprotease and disintegrin-like domains from proteolytic degradation. Because Sf21 and Hi5 cells recognize the same Nglycosylation sequon (Asn-X-Ser/Thr) as reptilian cells, the site-occupancy remains consistent with the native protein, preserving the overall topography of the toxin.

(3) Activity and authentic self-processing: We acknowledge that insect-specific alpha1,3-fucosylation can occur in Hi5 cells and is potentially antigenic. As the recombinant SVMPs will be used for binder selections and for testing *in silico* designed binders, useful binders will be selected based on neutralising activity against venom toxins. Here, our assays focused on auto-activation and proteolytic activity, which is primarily driven by the catalytic Zn^2+^-site and the protein backbone.

As stated above, analysis of glycosylation pattern of the PIII SVMP is a project on its own and beyond the scope of this manuscript.

We have incorporated some of the above information into the discussion section of the revised manuscript to clarify that insect cell glycosylation does not recapitulate the full diversity of SVMP glycosylation observed in native venoms.

(4) General comment for the bioassays: It would be good to specify the replicates again and report the data, including standard deviations.

We included this information in the figure legends.

Discussion:

I think the data generated in the study is very valuable and will be instrumental for pushing the frontiers in SVMP research, but still I would like to see a bit of modesty in their discussion. As I have pointed out above, it is unclear which effect the glycosilations may have (i.e., are the glycosilations found reminiscent of natural ones?), despite their being functionally important. Also, yes, isolation of SVMPs is challenging, but the reality is that their expression is equally challenging, as evidenced by the heaps of presented negative data (with which I have no problems, I think reporting such is actually important). So far, the "generic" protocol has been used to express one member per structural class of Echis SVMP, but no evidence is provided that it would work equally well on other members from taxonomically more distant snakes (e.g., the pIII known from Naja oxiana). It is very likely, but at the time of writing, purely speculative.

We have expressed additional PIII SVMPs from *Echis* and Daboia species and will report their production and characterisation in due course.

Lastly, the reality is also that the expression in insect cells can only be carried out by highly specialized labs (even in the expression world, as most laboratories work with bacterial or fungal hosts), whereas the isolation can be attempted in most venom labs. That said, production in insect cells also has economic repercussions as it will be very challenging to generate yields that are economically viable versus other systems, which is pivotal because the authors talk about bioprospecting and the toxins used in snakebite agent research.

We thank the reviewer for this perspective on the practicalities of protein expression. However, we respectfully disagree with the characterization of insect cell expression as an inaccessible or economically non-viable platform for toxin research. We offer the following points:

(1) Prevalence and accessibility: Contrary to the suggestion that insect cell expression is restricted to highly specialized labs, the Baculovirus Expression Vector System (BEVS) has become a cornerstone of modern biologics production, structural biology and biochemistry. For instance, our MultiBac system (which is but one of several systems currently widely in use) is utilised by over 1,000 laboratories and institutions, academic and pharma/biotech, worldwide. The maturation of commercially available kits, automated platforms, and standardized protocols has moved this technology into the mainstream, making it a standard tool for any lab requiring high-quality eukaryotic proteins.

(2) Biological necessity: Bacterial (*E. coli*) and fungal (*P. pastoris*) systems are widely accessible, however, they appear to be fundamentally incapable of producing functional SVMPs. SVMPs require complex disulfide-bond formation, intricate folding, and N-glycosylation for stability and solubility. Bacterial systems have been widely tried by us and others but typically result in very low expression or misfolded inclusion bodies. Of note, originally, we had invested significant effort to adapt *P. pastoris* to the production of eukaryotic proteins we are interested in, without success, before moving on to the MultiBac system. The SVMPs that we analysed here are highly cytotoxic, rendering the baculovirus/insect cell system in a way a logical choice given that the cells are no longer 'living' after infection with the baculovirus (but more akin membrane-enveloped bioreactors). Thus, one can make the argument that insect cells represent the most accessible middle ground that provides folding apparatus and necessary post-translational modifications (PTMs) required for biological relevance, and it is possible to produce mg amounts of SVMP proteins per litre cell culture as reported here in our manuscript.

(3) Economic viability and bioprospecting: Regarding the economic argument, we contend that viability in bioprospecting is defined by functional yield rather than simple volume. Producing large quantities of non-functional or misfolded protein in a cheaper system is economically inefficient. Furthermore, for snakebite research, the ability to produce specific, pure isoforms recombinantly without the contamination of other toxic venom components found in native isolations is essential for high-throughput screening and drug design.

(4) Scalability: Historically, insect cell production was seen as expensive, but current bioreactor technology and reduction in consumables and media costs allow for significant scaling. Many therapeutic reagents (vaccines, viral vectors, protein biologics) are produced routinely in baculovirus/insect cells. For the purposes of bioprospecting and lead identification, the yields provided by our Hi5/Sf21 system are sufficient for rigorous downstream bioassays and structural characterization.

Again, I believe the paper is highly important and excellently crafted, but I think especially the discussion should see some refinement to address the drawbacks and to evaluate the paper's findings with more modesty.

Thank you. We included the discussion about glycosylation patterns.

**Recommendations for the authors:**

**Reviewer #1 (Recommendations for the authors):**

(1) It is not entirely clear to me if the final constructs are indeed "fusion-proteins" (line 172, 974), in the sense of chimeric proteins. From the current description, it appears that the prodomain is encoded in the same gene rather than fused as a separate domain. Thus, referring to these constructs as fusion proteins may overstate the degree of protein engineering involved in the study.

This is correct. In the revised manuscript, ‘fusion protein’ is only used in the context of the propeptide SVMP fusion construct to avoid confusion.

(2) Figure 2J: It is difficult to assess how much protein is secreted relative to the intracellular amounts. The blot is surely misleading, as the effective protein dilution differs substantially between intracellularly vs. extracellularly. Providing an estimate of the relative dilution of extracellular protein would help clarify the extent of secretion.

We estimate that the SNP and SN fractions are at least 10-times more concentrated than the media fraction. The blot is analytical and not quantitative.

(3) The manuscript appears to use both alphafold 2 and alphafold 3 for structural predictions. Clarification on the choice of the version and its impact on results would improve consistency.

In the revised version of the manuscript, we clarify that all structural models were generated using AlphaFold 3.

(4) Figure S3b and others: a clear description of the antibodies used in the Western blots would be appreciated (including in the methods).

We included this information in the figure legends and a paragraph in the methods section for Western blots in the revised manuscript.

(5) MTT cytotoxicity testing would be more convincing if done in a concentration-dependent manner.

We repeated this assay using different concentrations of SVMPs and show the results as a new Figure 5f in the revised manuscript.

(6) Figure S3c: It could be interesting to show the sequence coverage to get an impression of what part of the protein is there.

We have included this information as Supplementary Figure S4d in the revised manuscript.

**Reviewer #2 (Recommendations for the authors):**

Overall, the study is presented in a step-by-step manner, and its conclusions are valid.

(1) As suggested in the public review, further characterization of the purified material would be good, for example, by intact mass-spectroscopy to characterize the enzymes in further detail.

Preliminary MALDI-MS analysis (performed in Loic Quinton’s laboratory) of our PIII SVMP revealed a broad and heterogeneous mass distribution, consistent with heterogeneity caused by the presence of multiple glycoforms (which is not unlike the microheterogeneity in native snake venom). However, owing to the inherent limitations of MALDI-MS for the analysis of glycoproteins, our data do not allow determination of the number of occupied N-glycosylation sites or the identification of additional types of glycosylation.

Moreover, the relatively large molecular mass of these proteins (zymogen 70.2 kDa protein only, mature PIII 50.6 kDa protein only) makes analysis by electrospray ionisation mass spectrometry technically challenging.

An MS-based deep analysis of the glycosylation patterns would therefore be a project on its own, and beyond the scope of the present manuscript.

(2) The studies involving PII appear challenging due to low yields and stability of the enzyme and the mentioned self-degradation. Some studies, such as the casein-degradation, would benefit from working with a well-characterized batch of enzymes to ensure, it is not auto-degrading during the experiment.

We believe that the finding that the PII SVMP degrades itself after incubation with Zn^2+^ is an important observation. It is novel to the best of our knowledge. Moreover, the key message of our manuscript is that we can produce and characterise novel SVMPs that cannot be readily purified from venom (and thus are not well characterised).

Besides, there are very few intact PII SVMPs in venom (e.g. Suntravat et al. BMC Molecular Biol 2016); the vast majority cleaves itself into a PI and a disintegrin.

(3) Figure 4h. Degradation of insulin is only shown for recombinant PIII, not the native enzyme, and therefore doesn't convey any information with respect to how well they compare.

We do not have available any native PII and PIII SVMPs for a comparison with the recombinant SVMPs (in our manuscript we show expression of new, uncharacterised SVMPs). We have included the PIII SVMP in the original manuscript to show that the enzyme is active and has a different specificity compared to PI SVMP. In the revised manuscript, we also included the PII SVMP insulin B degradation assay in Supplementary Figure S11b.

(4) Figure 5a. Inconsistent use of enzymes - data for PII is presented (both as mature protein and Zymogen) and compared to PIII, but not PI, as both zymogen and mature protein. The current data presentation is confusing and gives the idea of the manuscript assembled with figures produced during the exploratory phase of the study, and not from subsequent experiments systematically conducted for the purposes of clarity and completeness.

In the revised manuscript, we included the missing enzymatic characterisations in Figure 5 (panel a and e) and Supplementary Figure S11a-c. These data were initially not included because the respective enzymes are inactive in these assays.

(5) The manuscript would benefit from editing to make it more concise. For an early-career reader, it is of interest and utility to follow the thought and experimental processes that led to the successful solution, but there is a risk of losing the reader's interest along the way by going through expression experiments that did not "work" in the typical sense of the word. To this reviewer, there is no added value in a full paragraph around co-expression with disulfide isomerase, as it did not improve the protein yield. A single sentence, "co-expression with PDI did not improve yields," with a reference to a supplemental figure would convey that message.

We have moved the optimisation of SVMP expression to the Supplementary Information, which we hope has improved the clarity and flow of the main text.

We note that the hypothesis that co-expression of protein disulfide isomerases (PDIs) enhances yields of functional SVMPs, given the high expression of PDIs in snake venom gland cells, is well established in the field. While we consider PDIs (and other chaperones) likely to play an important role in SVMP expression, we were unable to demonstrate this effect using the baculovirus-insect cell expression system and hypothesize that efficient insect and/or baculoviral PDIs are already present.

(6) Similarly with N-linked glycosylation, the section needs a headline (line 241) and firming up of a sentence like "and possibly not all of the glycosylation..." which is vague and appears to state that it was not really of interest to pursue this further. My view is that either an experiment is done properly with a stated aim and purpose, interpreted, and then, based on whether the results are of interest to the main story or not, they are included. If N-linked glycosylation is to be included in the manuscript, it should be with a purpose (e.g., N-linked glycosylation affects enzyme activity). As it stands, the message is "there is some N-linked glycosylation" without further explanation, and this generates information without justifying the inclusion hereof.

Please see our reply above regarding an in-depth characterisation of insect cell glycosylation of the recombinant PIII SVMP without access to the native enzyme for comparison. In our revised manuscript, we confirm that the PIII SVMP is glycosylated and that this at least partly accounts for the apparent discrepancy in molecular weight observed in SEC and SDS PAGE. We have modified the text to clarify the purpose of the PNGase deglycosylation experiment.

(7) The manuscript, in its current form, appears to have been copied from a Thesis with very detailed step-by-step logic and description. While this is useful in a scholarly context, a scientific manuscript should be presented more compactly, assuming the readers know basic biochemistry.

We trust that this Reviewer finds the revised version of our manuscript more compact and concise.

**Reviewer #3 (Recommendations for the authors):**

(1) Material and Methods plus Figures:

Please report the number of replicates per experiment and how data is presented (means/ medians/ standard deviation/ others), and add error bars to the plots where needed.

In the revised manuscript we have included the number of repeats in the figure legends.

(2) Abstract

Line 4: I would not say that SVMPs are the most potent viper toxins. This place is probably taken by some of the highly neurotoxic PLA2, such as Crotoxin. Nevertheless, SVMPs are surely some of the most important toxins responsible for pathophysiological effects stemming from viper envenoming, but I would suggest rephrasing for accuracy.

In the revised manuscript, we have modified this sentence.

(3) Introduction

Lines 27-31: I would like to see a reference supporting the existence of all SVMP types across vipers.

We have included references supporting the existence of PI, PII and PIII SVMPs in viper venom. We also rewrote the sentence to state that “representatives of all three sub-classes are present in different viper venoms.” This clarifies that we do not say that all classes are present in all venoms.

Lines 59-60: I am not sure if this should be considered such an important impediment. Essentially, many vipers yield double- to triple-digit mg amounts of crude venom per specimen from only a single milking.

We have rewritten this text in the revised manuscript.

Currently, it is not possible to purify any given SVMP of interest from venom; in particular for *E. ocellatus* SVMP isoform mixtures are typically purified rather than individual enzymes (see also introduction section of our manuscript line 57ff). Also, many SVMPs are not present in sufficient amounts in the venom. Here, we provide an approach to recombinantly produce any SVMP of interest, independent of its abundance in the venom.

(4) Results

Line 102: The army-fallworms name is Spodoptera, not Spotoptera. Please correct the typo.

Done. Apologies for our oversight.

Line 311: Please provide the data at least as a supplement.

In the revised manuscript, we have included this experiment in Supplementary Figure S6c.

Line 432- 433: It would be useful to clarify whether the protein should have a pro-coagulant activity (or not).

We have changed this sentence as follows in the revised manuscript: This shows that our recombinantly produced SVMPs have no pro-coagulant activity, which was unknown before.
